# Risk factors affecting COVID-19 vaccine effectiveness identified from 290 cross-country observational studies until February 2022: a meta-analysis and meta-regression

**DOI:** 10.1186/s12916-022-02663-z

**Published:** 2022-11-25

**Authors:** Marek Petráš, Roman Máčalík, Daniela Janovská, Alexander M. Čelko, Jana Dáňová, Eliška Selinger, Jonáš Doleček, Sylva Neradová, Martina Franklová, Pavel Dlouhý, Jozef Rosina, Ivana Králová Lesná

**Affiliations:** 1 Department of Epidemiology and Biostatistics, Third Faculty of Medicine, Charles University, Ruská 87, 100 00 Prague, Czech Republic; 2 Centre for Public Health Promotion, National Institute of Public Health, 100 00 Prague, Czech Republic; 3 Department of Hygiene, Third Faculty of Medicine, Charles University, 100 00 Prague, Czech Republic; 4 Department of Medical Biophysics and Informatics, Third Faculty of Medicine, Charles University, 100 00 Prague, Czech Republic; 5 Department of Health Care and Population Protection, Faculty of Biomedical Engineering, Czech Technical University in Prague, 272 01 Kladno, Czech Republic; 6 Laboratory for Atherosclerosis Research, Centre for Experimental Medicine, Institute for Clinical and Experimental Medicine, 140 21 Prague, Czech Republic; 7 Department of Anesthesia and Intensive Medicine, First Faculty of Medicine, Charles University and University Military Hospital, 100 00 Prague, Czech Republic

**Keywords:** COVID-19, Vaccine effectiveness, Protection decline, mRNA vaccine, Adenoviral vector vaccines

## Abstract

**Background:**

Observational studies made it possible to assess the impact of risk factors on the long-term effectiveness of mRNA and adenoviral vector (AdV) vaccines against COVID-19.

**Methods:**

A computerized literature search was undertaken using the MEDLINE, EMBASE, and MedRxiv databases to identify eligible studies, with no language restrictions, published up to 28 February 2022. Eligible were observational studies assessing vaccine effectiveness (VE) by disease severity with reference groups of unvaccinated participants or participants immunized with one, two, or three vaccine doses. Our study was carried out in compliance with the PRISMA and MOOSE guidelines. The risk of study bias was identified using the Newcastle–Ottawa Quality Assessment Scale. The GRADE guidelines were applied to assess the strength of evidence for the primary outcome. The synthesis was conducted using a meta-analysis and meta-regression.

**Results:**

Out of a total of 14,155 publications, 290 studies were included. Early VE of full vaccination against COVID-19 of any symptomatology and severity decreased from 96% (95% CI, 95–96%) for mRNA and from 86% (95% CI, 83–89%) for AdV vaccines to 67% for both vaccine types in the last 2 months of 2021. A similar 1-year decline from 98 to 86% was found for severe COVID-19 after full immunization with mRNA, but not with AdV vaccines providing persistent 82–87% effectiveness. Variant-reduced VE was only associated with Omicron regardless of disease severity, vaccine type, or vaccination completeness. The level of protection was reduced in participants aged >65 years, with a comorbidity or those in long-term care or residential homes independently of the number of doses received. The booster effect of the third mRNA dose was unclear because incompletely restored effectiveness, regardless of disease severity, declined within a short-term interval of 4 months.

**Conclusions:**

Full vaccination provided an early high, yet waning level of protection against COVID-19 of any severity with a strong impact on the high-risk population. Moreover, the potential risk of new antigenically distinct variants should not be underestimated, and any future immunization strategy should include variant-updated vaccines.

**Supplementary Information:**

The online version contains supplementary material available at 10.1186/s12916-022-02663-z.

## Background

The global efforts to fight the COVID-19 pandemic led to the rapid development of various types of vaccines receiving emergency use authorization by regulators of the USA (Food and Drug Administration), the European Union (European Medicines Agency), and the World Health Organization at the turn of 2020 and 2021 [1–3]. Although early and short-term clinical trials suggested the high efficacy of these vaccines [4, 5], especially against severe COVID-19, their use aroused great interest of numerous research teams worldwide to establish real-world vaccine effectiveness (VE) [6–10].

Within the first 14 months of the start of the vaccination campaign, hundreds of studies either specifically focusing on or marginally reporting the effectiveness of various COVID-19 vaccine types were conducted in different populations around the world. Vaccine effectiveness was assessed by the number of received doses, post-vaccination time, participants’ age, sex, or health status as well as by new circulating SARS-CoV-2 variants, and was mostly related to the level of protection against any SARS-CoV-2 infection regardless of symptomatology or against COVID-19 of any severity, or against severe COVID-19 requiring hospitalization or resulting in death.

Although dozens of various reviews or meta-analyses have attempted to assess the effectiveness of vaccination against COVID-19 for different types of vaccines, [9, 11, 12], with regard to different coronavirus variants [9, 11, 13, 14], risk groups [12, 15–17], or the length of time after vaccination [15, 18], no comprehensive study focused on the association of multiple factors with the vaccine effectiveness achieved has yet been published. Therefore, we decided to conduct a large meta-analysis extended with meta-regression referred to as “meta-COVANESS” (META-analysis/regression of COvid VAccine effectiveNESS) and to quantitatively evaluate the outcomes published during the first 2.5 years of immunization.

Overall, we were able to identify 290 eligible studies published until the end of February 2022, which enabled us to perform the first quantitative analyses focused on VE assessment for the first year of global vaccination against COVID-19, that is, when pre-Omicron variants were still prevalent. As post-vaccination protection waned over time, the concept of full vaccination against COVID-19 was later expanded to include one booster dose. The worldwide booster immunization campaign was launched in the second half of 2021 [19, 20]. Therefore, we sought to primarily assess the effectiveness of full vaccination, while the effectiveness of partial or booster immunization was considered a secondary outcome of meta-COVANESS.

The aim of this analysis was to identify the risk factors negatively impacting the effectiveness of vaccination. For this purpose, we extracted VE records from observational studies (cohort, case–control, or cross-sectional ones) conducted in the general population or in specific ones according to sex, age stratification, comorbidities, profession, and communities. Other considerations included the type of vaccine or virus variant and the study outcome was evaluated with respect to symptomatology and/or severity of the disease.

## Methods

Quantitative analyses were conducted according to the Preferred Reporting Items for Systematic Review and Meta-Analyses (PRISMA) guidelines (Additional file 1: Table S1, Table S2) [21–23] and Meta-analysis Of Observational Studies in Epidemiology (MOOSE) guidelines (Additional file 1: Table S3) [24]. Meta-COVANESS was registered in the International prospective register of systematic reviews (CRD42022301503).

### Search strategy

A computerized search of the relevant literature was undertaken using the MEDLINE and Excerpta Medica dataBASE (EMBASE) databases and MedRxiv, a free online archive of complete preprints. Eligible studies, with no language restriction, had to be published between 1 December 2020 and 28 February 2022. Moreover, weekly searches of MEDLINE via PubMed were conducted over the same period of time and a recursive search of references in published reviews or meta-analyses was done manually to identify additional studies beyond the computerized search.

Relevant publications were selected using “immunization,” “COVID-19,” and “effectiveness” as keywords and their synonyms. The full search formulas are shown in Additional file 2: Table S4.

### Study selection

Eligible studies met the following inclusion criteria: (1) observational studies with a control group represented by unvaccinated participants, (2) exposure defined by type-specific or commercial vaccines including the number of doses, (3) outcome assessed by disease of any symptomatology and severity, and (4) stated VE including the confidence interval (CI). The primary outcome of our meta-analysis was to identify the risk factors of VE at least 7 days after full immunization (i.e., usually after two vaccine doses as defined by the manufacturer). The secondary outcomes of partial vaccination (with one vaccine dose) or booster immunization (after three doses) were supportive to the primary outcome. The first year of the worldwide vaccination campaign was dominated by four FDA- or EMA-approved vaccines, i.e., two mRNA vaccines: BNT162b2 (Comirnaty; Pfizer-BioNTech) and mRNA-1274 (Spikevax; Moderna) and two adenoviral vector (AdV) vaccines: ChAdOx1 (Vaxzevria, AstraZeneca) and Ad26.COV2.S (Jcovden, Janssen). Therefore, only studies reporting homologous immunization with these vaccines or with these vaccine types were eligible for our quantitative synthesis.

### Data extraction and quality assessment

Extracted data included characteristics of the study, participants, interventions, comparison, and outcome. Studies scoring ≥7 stars using the Newcastle–Ottawa Quality Assessment Scale (NOS) were defined as low risk of bias (RoB) (Additional file 3) [25–29]. To assess the quality of evidence resulting from this quantitative synthesis, the Grading of Recommendations Assessment, Development, and Evaluation (GRADE) [30] criteria were considered: number of VE records, study limitations, heterogeneity, and indirectness based on the difference between vaccinated and unvaccinated participants, imprecision, and publication bias of studies.

### Data analysis

The meta-analysis was conducted to obtain pooled VE data stratified according to risk factors. These risk factor categories included sex; age-stratified sex; age stratified as <18 years, 18–65 years, and >65 years; patients with specified comorbidities (i.e., type 2 diabetes mellitus, hypertension, obesity, cardiovascular, renal, respiratory, neurological, and liver diseases, cancer, immunosuppression or transplantation, etc.); and the specific population of healthcare workers (HCWs) or high-risk groups (HRGs) including residents of long-term care or residential homes. The key predictors were the post-vaccination interval and the SARS-CoV-2 variant. The very often missing post-vaccination interval was compensated for by the month of study termination stratified into 2-month intervals from January 2021 to February 2022. Variant-dependent VE records were assessed as untyped, with the α, β, and γ variants merged into one group and the δ or ο variants assessed separately.

Given the high heterogeneity of extracted VE records resulting from an inconsistency index >50%, quantitative synthesis was conducted using the random-effects model (DerSimonian–Laird method; D–L). The fixed-effect model (inverse variance method, I-V) was used to reveal a potential publication bias. The primary analysis was performed as a meta-regression where the dependence of log-transformed effect sizes derived from VE as 1–VE/100 on the categorical independent variables representing the risk factors was investigated. To obtain adjusted VE, the most robust reference subgroup of each factor was selected. Additional analyses were performed to assess (1) the effect of small studies determined using a meta-regression model with Egger’s test [31], (2) the summary effect of asymmetry with the identification of any unpublished or unfound studies estimated by the trim-and-fill method [28], and (3) the prediction interval estimated from the standard errors of studies heterogeneity and pooled VE [28]. Statistical analyses were performed using STATA version 17.0 (StataCorp. 2021. Stata Statistical Software. College Station, TX, USA) with two main modules of “meta summarize” and “meta regress” at a significance level of *α* = 0.05 with a two-tailed 95% CI.

## Results

**Fig. 1:**
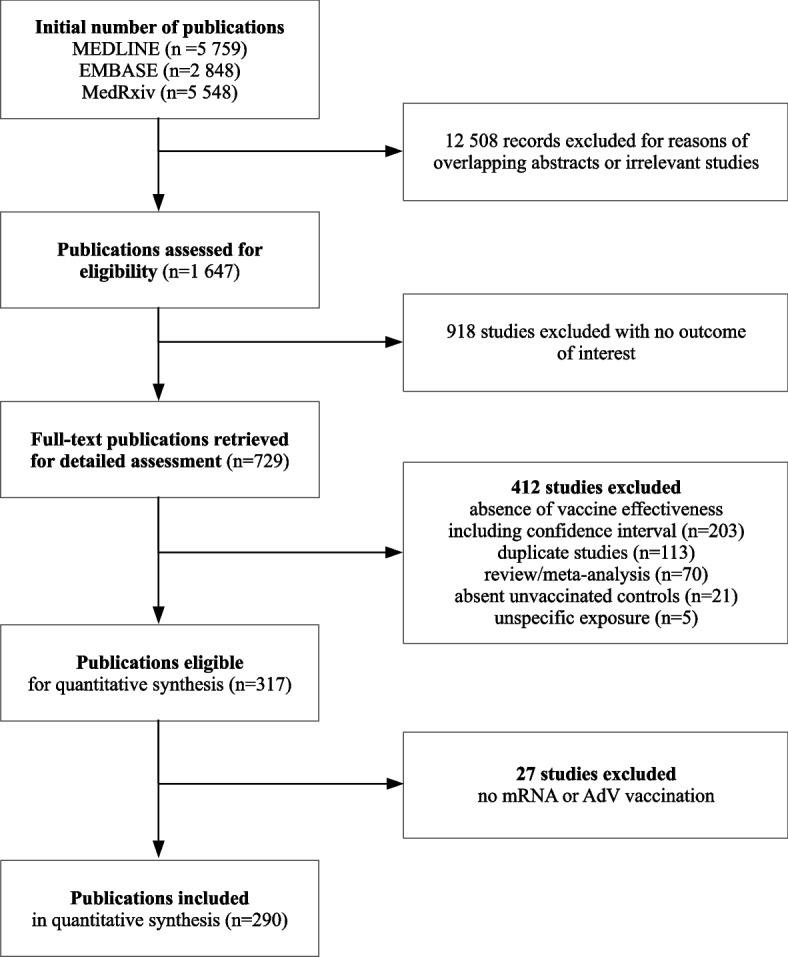
Flowchart

A total of 14,155 publications were identified, of which 13,837 were excluded for reasons of irrelevant or duplicate studies and absent inclusion criteria (Fig. 1). Although the remaining 317 studies did meet the inclusion criteria of meta-COVANESS, a total of 290 published studies with 4867 extracted VE records related to mRNA or AdV vaccination were included in the quantitative synthesis (Additional file 4: Table S5). The VE records were grouped according to vaccine type (mRNA or AdV), number of doses (i.e., partial vaccination and mRNA- or AdV-homologous full and booster immunization), and severity of disease, i.e., COVID-19 regardless of symptomatology or severity (“any COVID-19” hereinafter) or severe COVID-19 including hospitalization and death. Booster vaccination with AdV vaccines was omitted because of the small number of records, and one-dose immunization with the Ad26.COV2.S vaccine was considered full immunization. A total of 3862 VE records were extracted for mRNA vaccination and 1005 VE records for AdV vaccination (Additional file 5: Table S6). The number of extracted VE records including that of studies grouped into the investigated predictors is reported in Table 1.

**Table 1:** Number of extracted VE records of partial, full, and booster immunization for any or severe COVID-19 according to investigated predictors (including the number of studies in brackets)

Predictors	Vaccine	mRNA						AdV			
	Immunization against	Any COVID-19			Severe COVID-19			Any COVID-19		Severe COVID-19	
	Number of vaccine doses	1	2	3	1	2	3	1	2	1	2
**COVID-19**	**Infection**	423 (109)	1165 (160)	76 (14)				91 (29)	180 (42)		
	**Disease**	146 (48)	630 (69)	50 (6)				59 (20)	220 (26)		
	**Hospitalization**				234 (50)	791 (96)	85 (18)			78 (23)	220 (38)
	**Death**				51 (28)	199 (42)	12 (7)			37 (17)	104 (26)
**Study termination**	**Jan–Feb 2021**	97 (24)	61 (13)		39 (5)	20 (2)		7 (3)		13 (1)	
	**Mar–Apr 2021**	100 (39)	160 (52)		53 (17)	81 (20)		12 (4)	7 (3)	3 (3)	5 (2)
	**May–Jun 2021**	124 (35)	230 (57)		70 (18)	123 (26)		65 (22)	88 (24)	40 (15)	70 (16)
	**Jul–Aug 2021**	68 (12)	271 (32)		59 (9)	132 (20)		34 (7)	89 (15)	44 (9)	60 (13)
	**Sep–Oct 2021**	117 (19)	775 (37)		35 (11)	410 (26)		15 (5)	180 (20)	13 (6)	156 (17)
	**Nov–Dec 2021**	25 (8)	202 (25)	77 (13)	18 (5)	174 (18)	66 (14)	15 (2)	23 (8)	2 (1)	33 (5)
	**Jan–Feb 2021**	38 (3)	96 (8)	49 (7)	11 (2)	50 (9)	31 (7)	2 (1)	13 (2)		
**Variants**	**Untyped**	413 (111)	1262 (158)	56 (9)	221 (55)	770 (94)	71 (14)	122 (36)	272 (50)	97 (30)	256 (43)
	**α, β, γ**	58 (16)	78 (21)		33 (8)	49 (16)		14 (5)	10 (5)	7 (2)	8 (2)
	**δ**	77 (23)	380 (47)	29 (10)	30 (9)	150 (25)	12 (7)	13 (7)	109 (16)	11 (5)	60 (10)
	**ο**	21 (4)	75 (16)	41 (12)	1 (1)	21 (8)	14 (8)	1 (1)	9 (4)		
**Commercial vaccine**	**BNT162b2**	341 (91)	927 (137)	52 (12)	154 (36)	458 (70)	35 (8)				
	**mRNA-1273**	92 (29)	504 (57)	43 (9)	48 (14)	247 (39)	19 (6)				
	**ChAdOx1**							144 (39)	185 (40)	108 (29)	167 (28)
	**Ad26.COV2.S**								187 (20)		111 (18)
**Sex**	**Men**	22 (12)	60 (17)	4 (2)	4 (3)	15 (7)	2 (1)	1 (1)	3 (2)	1 (1)	5 (3)
	**Women**	25 (14)	51 (20)	4 (2)	4 (3)	14 (7)	2 (1)	1 (1)	3 (2)	1 (1)	5 (3)
**Age**	**<18 years**	45 (8)	98 (14)		2 (2)	12 (6)	1 (1)		14 (1)		
	**18–65 years**	110 (29)	430 (50)	17 (5)	76 (19)	232 (40)	20 (7)	30 (10)	108 (16)	25 (9)	77 (19)
	**>65 years**	105 (30)	251 (51)	8 (5)	72 (22)	221 (43)	11 (7)	31 (12)	68 (17)	35 (11)	98 (22)
**Comorbidities**	**Undetermined**	510 (123)	1 680 (179)	110 (19)	241 (57)	881 (104)	81 (20)	138 (40)	385 (56)	94 (32)	296 (46)
	**Specified**	59 (17)	115 (31)	16 (5)	44 (16)	109 (30)	16 (6)	12 (2)	15 (8)	21 (3)	28 (8)
**Population**	**General**	426 (69)	1 520 (117)	115 (17)	254 (46)	893 (88)	87 (18)	126 (28)	372 (45)	109 (29)	309 (40)
	**HCW**	101 (44)	185 (51)		8 (4)	17 (2)		18 (11)	14 (10)	6 (4)	8 (5)
	**HRG**	42 (21)	90 (30)	11 (3)	23 (15)	80 (25)	10 (2)	6 (2)	14 (6)		7 (4)

*AdV* adenoviral vector vaccine, *HCWs* healthcare workers, *HRG* high-risk group (individuals living in long-term care or residential homes)

### Full vaccination

The level of protection against any COVID-19 within 14 months was documented by the pooled 81% VE (95% CI, 81–82%) for mRNA vaccines and 64% VE (95% CI, 63–65%) for AdV vaccines. Both pooled VE values against severe COVID-19 rose to 92% (95% CI, 91–92%) and 85% (95% CI, 84–86%) after mRNA and AdV vaccination, respectively.

**Fig. 2:**
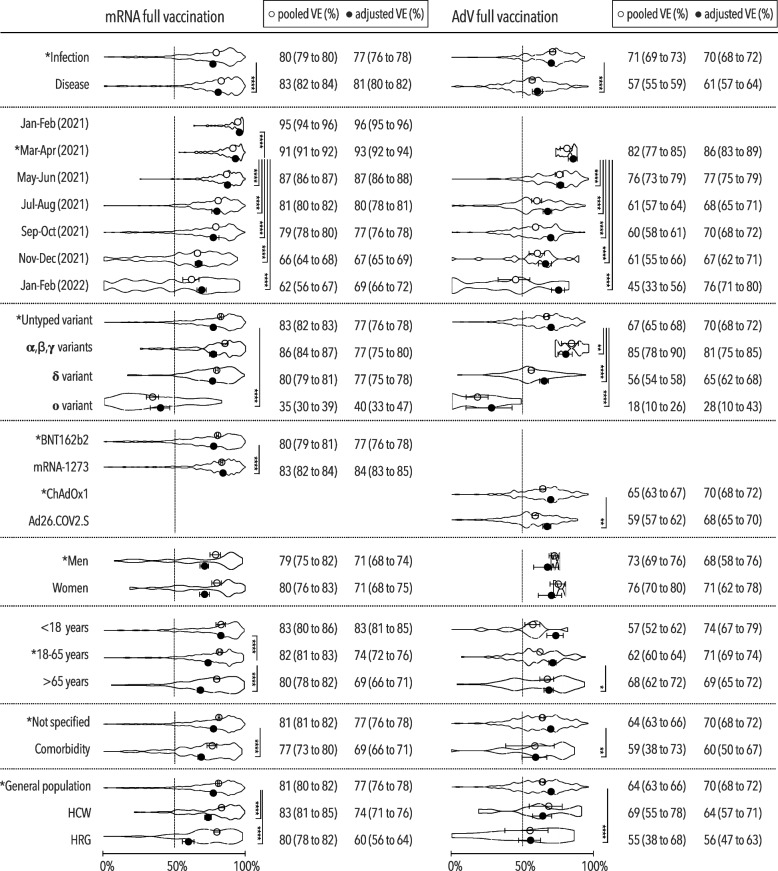
Vaccine effectiveness against any COVID-19 after full immunization. Pooled and adjusted vaccine effectiveness against any COVID-19 for investigated predictors with VE density displayed in truncated violin plots after full mRNA and AdV immunization, including the minimum 50% VE threshold set by WHO [32]; AdV adenoviral vector vaccine, VE vaccine effectiveness, HCW healthcare worker, HRG high-risk group (individuals living in long-term care or residential homes); *p<0.05; **p<0.01; ***p<0.001; ****p<0.0001

**Fig. 3:**
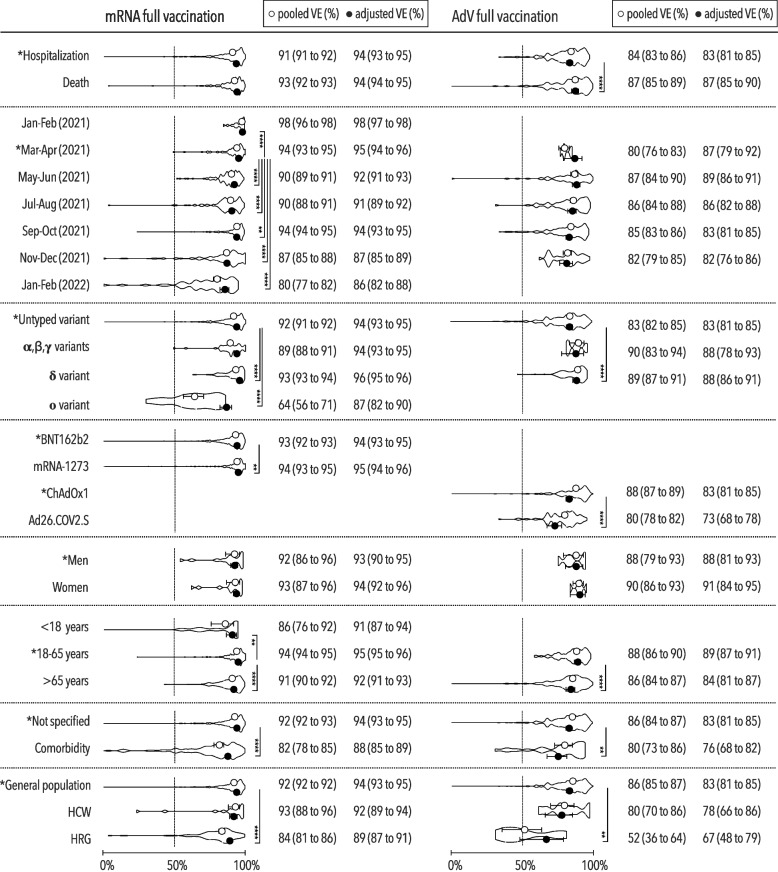
Vaccine effectiveness against severe COVID-19 after full immunization. Pooled and adjusted vaccine effectiveness against severe COVID-19 for investigated predictors with VE density displayed in truncated violin plots after full mRNA and AdV immunization, including the minimum 50% VE threshold set by WHO [32]; AdV adenoviral vector vaccine, VE vaccine effectiveness, HCW healthcare worker, HRG high-risk group (individuals living in long-term care or residential homes); *p<0.05; **p<0.01; ***p<0.001; ****p<0.0001

However, adjusted VE against any COVID-19 depended on study termination regardless of vaccine type. The early 96% VE (95% CI, 95–96%) observed after mRNA vaccination in January–February of 2021 decreased significantly to 67% (95% CI, 65–69%) by November–December of 2021 (Fig. 2). The waning protection against severe COVID-19 was also demonstrated by the significantly decreasing effectiveness specific only for mRNA vaccines, i.e., from 98% (95% CI, 97–98%) to 86% (95% CI, 82–88%) a year later (Fig. 3). A similar decline occurred after AdV vaccination, i.e., the 86% VE (95% CI, 83–89%) in March–April of 2021 dropped to 67% (95% CI, 62–71%) in November–December of 2021. The waning protection against severe COVID-19 was also demonstrated by the significantly decreasing effectiveness specific only for mRNA vaccines, i.e., from 98% (95% CI, 97–98%) to 86% (95% CI, 82–88%) a year later. However, this decline was slower compared with that for any COVID-19.

Protection against any COVID-19 was not negatively affected by the pre-Omicron variants as demonstrated by the non-inferior VE value associated with specific variants versus variant-untyped VE.

Nevertheless, the Omicron variant significantly decreased the VE against any COVID-19 to 40% (95% CI, 33–47%) and that against severe COVID-19 to 87% (95% CI, 82–90%) after mRNA vaccination. A decrease in protection against any COVID-19 associated with Omicron, i.e., 28% VE (95% CI, 10–43%), was also apparent in participants immunized with AdV vaccines.

Other risk factors decreasing protection against any COVID-19 included age >65 years, comorbidities, and living in long-term care or residential homes. It was just these specific populations, which exhibited decreases by 14–17% and 5–16% in VE against any and severe COVID-19, respectively, independently of vaccine type. Higher levels of effectiveness irrespective of disease severity were observed in participants vaccinated with either the mRNA-1273 or ChAdOx1 vaccine versus those vaccinated with the same type of vaccine, i.e., BNT162b2 or Ad26.COV2.S. Full immunization with mRNA vaccines conferred better protection against disease than against infection whereas protection against infection and disease was just the opposite after AdV vaccination. The VE against death was not inferior to that against hospitalization regardless of vaccine type.

### Booster vaccination

**Fig. 4:**
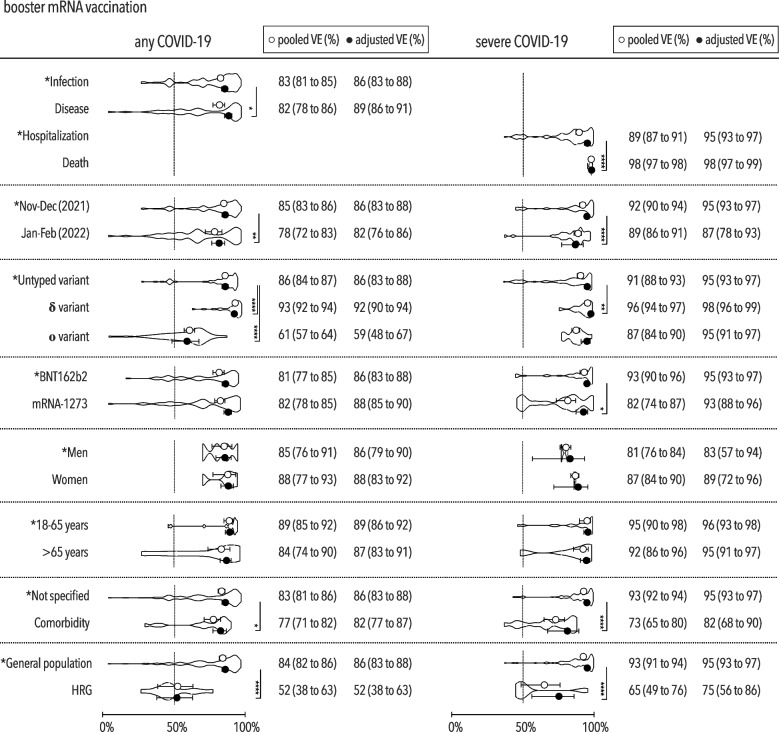
Vaccine effectiveness against any and severe COVID-19 after booster immunization. Pooled and adjusted vaccine effectiveness against any and severe COVID-19 for investigated predictors with VE density displayed in truncated violin plots after booster mRNA immunization, including the minimum 50% VE threshold set by WHO [32]; VE vaccine effectiveness, HRG high-risk group (individuals living in long-term care or residential homes); *p<0.05; **p<0.01; ***p<0.001; ****p<0.0001

The overall pooled VE values of booster immunization with mRNA vaccines were 83% (95% CI, 80–85%) against any COVID-19 and 91% (95% CI, 90–93%) against severe COVID-19. The level of protection depended on the month of study termination as demonstrated by the significant decline in January or February of 2022 for any or severe COVID-19 (Fig. 4).

The booster VE against any COVID-19 caused by the Omicron variant decreased to 59% (95% CI, 48–67%) while remaining unchanged against severe COVID-19.

An increased risk of any COVID-19 was found in patients with a comorbidity or those living in long-term care or residential homes showing lower protection than the general population after booster vaccination.

Higher levels of protection were achieved against disease compared with infection and death versus hospitalization. Although no different adjusted VE against any COVID-19 was found between both commercial mRNA vaccines, a 2% higher level of protection against severe COVID-19 was observed with the BNT162b2 versus the mRNA-1273 vaccine.

### Partial vaccination

The effectiveness of partial vaccination was influenced by risk factors identical to those of full immunization (Additional file 6: Fig. S1; Additional file 7: Fig. S2). Incomplete immunization with any mRNA vaccine provided pooled VE values of 58% (95 CI% 55–60%) and 73% (95% CI, 71–75%) against any and severe COVID-19, respectively, which were significantly decreased in the last two 2-month intervals of 2021 regardless of COVID-19 severity. The pooled VE values against any and severe COVID-19 were 47% (95% CI, 45–49%) and 70% (95% CI, 68–73%), respectively, after one-dose immunization with the ChAdOx1 vaccine. Despite the waning levels of protection against any COVID-19 observed between May and December of 2021, these levels against severe disease did not change significantly within 12 months.

Protection by partial immunization with an mRNA or AdV vaccine either against any or severe COVID-19 was significantly decreased by the Omicron variant and in specific risk populations. Among the mRNA vaccines, better protection was shown with one mRNA-1273 dose regardless of COVID-19 severity.

### Quality of evidence

A sufficient number of VE records was obtained for the predefined predictors except for sex and age <18 years. Over 70% of records assessing partial or full vaccination against COVID-19 of any symptomatology came from studies at low RoB; hence, no serious limitation was anticipated. Conversely, the quality of evidence was lower for outcomes of severe COVID-19 and booster immunization since the analyses were more often performed using records from studies with an unclear limitation, i.e., 46–69% of records came from low RoB studies. The random-effect model had to be applied because of the high heterogeneity of studies.

Partial and full immunization regardless of vaccine type and disease severity were investigated using ≥80% records of studies with no differences among participants; therefore, no serious indirectness of evidence was assumed. The indirectness of evidence assessing booster immunization was unclear since 54–64% records achieved acceptable comparability of vaccinated and unvaccinated participants. No serious imprecision of outcomes was found as demonstrated by a pooled standard error <2%.

Publication bias established by the difference of outcomes of both random-effect and fixed-effect models was confirmed regardless of the number of doses, COVID-19 severity, or vaccine type. However, as the difference between results of both models was <0.1, publication bias was considered acceptable. Quantitative analyses were burdened by the effect of small studies increasing pooled VE with missing studies decreasing pooled VE. Moreover, the prediction intervals of full vaccination confirmed that the VE against any COVID-19 ranged between 44 and 94% for mRNA and between 24 and 83% for AdV vaccines and against severe COVID-19 between 72 and 97% for mRNA and between 50 and 96% for AdV vaccines. The same intervals for the booster dose of an mRNA vaccine ranged between 10 and 97% for any COVID-19 and between 43 and 99% for severe COVID-19. A summary of acceptable/no serious and unclear strengths of evidence for studies grouped according to investigated predictors is available in Additional file 8: Table S7.

## Discussion

Studies published during the first 14 months of COVID-19 immunization indicated an overall VE of 81% for mRNA and 64% for AdV vaccines regardless of disease symptomatology or severity, i.e., a level of protection slightly lower than that established by early clinical trials with an about 2-month follow-up, i.e., 95% for BNT162b2 [33] and 94% for mRNA-1274 [34], and 70% for ChAdOx1 [35] and 67% for Ad26.COV2.S [36]. Nevertheless, protection against severe COVID-19 of 92% for mRNA and 85% for AdV vaccines approached the outcomes of short-term clinical trials. One can thus conclude that the effectiveness against hospitalization or death lasted longer after full immunization regardless of vaccine type.

Meta-COVANESS identified the factors negatively impacting the level of protection of COVID-19 vaccination. One of the strong risk factors was the post-vaccination period compensated for, in this analysis, by study termination. The VE values of partial, full, and booster immunization tended to decline over 2-month intervals according to study termination, with the decline being marked for disease of any severity or symptomatology. Similar outcomes were reported by other studies assessing time-dependent effectiveness [37–44]. The 20–32% decline in VE against infection or disease within 6 months after full vaccination with mRNA or AdV vaccines was found in a previous meta-regression [18]. Our analysis did not document the level of protection waning so quickly because the VE against infection or disease declined by about 19–29% within 12 months. This discrepancy could be explained by the proxy post-vaccination period in our study.

Although, among both vaccine types, full AdV vaccination conferred less protection against severe COVID-19, its durability ranging between 82 and 87% was confirmed independently of study termination. Unfortunately, current data did not make it possible to establish if this effect depends specifically on a particular vaccine or can occur with any. Some studies suggested very good protection persistence following Ad26.COV2.S immunization [45, 46].

Another important factor influencing post-vaccination protection was the new Omicron variant antigenically absolutely distinct from pre-Omicron variants [47, 48]. Its impact was demonstrated by the robust decline in effectiveness against disease of any severity independently of the number of doses. Whether or not and how AdV vaccines could fail in conferring protection against severe disease has not been established because of absent studies. Although the mRNA booster dose increased the VE to 59% and 95% against any and severe COVID-19, respectively, it was unclear how long it would persist.

Pre-Omicron variants did not have any negative effect on VE against infection as indicated by the non-inferior protection after partial or full immunization irrespective of vaccine type. One can thus only speculate about the early outcomes of reviews or meta-analyses assessing the impact of particular variants that suggested reduced VE of full immunization by about 10–30% for the beta variant, [9, 11, 49], 0–30% for the gamma variant [9, 49], or 10–45% for the delta variant [9, 11, 13, 49] compared with the alfa variant. The decline associated with pre-Omicron variants may have been confounded by the length of the post-vaccination period. Furthermore, lower VE was observed in participants aged >65 years, in those with a comorbidity, and in those living in long-term care or residential homes. Although the present analysis did not determine which of the above factors is decisive and crucial for the decreased effectiveness, it is likely that a common feature of these participants could be an immunodeficiency disorder as shown by studies in these patients [50, 51].

Our analyses confirmed that the second vaccine dose contributed significantly to increased protection against COVID-19 whatever its severity. However, the insufficient number of studies made it impossible to determine the potential importance of the contribution of the second dose of the Ad26.COV2.S vaccine. While two studies suggested a >82% effectiveness against any COVID-19 independently of the variant [52, 53], another one showed only a 6% VE against infection caused by the Omicron variant [54].

The booster mRNA vaccine dose resulted in the incomplete restoration of the 86% level of protection against any COVID-19 versus 96% after a 2-dose mRNA vaccination. The same effect of the booster dose was found for severe disease, i.e., 95% after the third dose versus 98% after the second dose. Moreover, protection durability elicited by the booster dose exhibited a significant decline in studies terminated 2 months later.

A certain limitation of our study consisted in predetermined approaches assuming the same or similar impact of vaccine types (independently of the brand name) and similar effect of composite COVID-19 (any and severe). Although the effectiveness of all commercial vaccines assessed by us was different and the vaccine-elicited levels of protection were not identical for infections of any symptomatology and disease of any severity, the adopted approaches were acceptable with no serious influence on overall outcomes.

The quality of evidence was sufficient for outcomes of full vaccination although publication bias as well as the effect of small studies and imputed studies was found by additional analyses. Unfortunately, the evidence of outcomes for booster immunization was restricted by the smaller number of records including higher proportion of studies with unclear limitation or indirectness. Therefore, the outcomes of booster immunization should be interpreted with caution.

## Conclusions

This study conclusively confirmed early VE, higher for mRNA versus AdV vaccines, becoming similar with both within 14 months regardless of COVID-19 severity.

While the pre-Omicron variants were not associated with a negative impact on post-vaccination protection, the Omicron variants critically influenced the effectiveness irrespective of vaccine type or disease severity. A higher risk of protection failure was found in persons with likely immune system disorders as a potentially serious issue. The unclear booster effect of the third mRNA dose was demonstrated by incompletely restored short-term protection with a gradual decline. Therefore, further observations are warranted to develop a future strategy for multiple booster vaccination or revaccination with updated vaccines.

## Supplementary Information

**Additional file 1. **PRISMA-S Checklist (**Table S1**), PRISMA Checklist (**Table S2**), MOOSE Checklist (**Table S3**)

**Additional file 2. **Search strategy (**Table S4**).

**Additional file 3.** Data extraction, assessment of evidence quality, assessment of RoB.

**Additional file 4. **Studies included in quantitative synthesis (**Table S5**).

**Additional file 5. **Descriptive review of eligible studies, including RoB (NOS stars) (**Table S6**).

**Additional file 6. **Pooled and adjusted vaccine effectiveness against any COVID-19 after partial mRNA and AdV immunization (**Figure S1**).

**Additional file 7. **Pooled and adjusted vaccine effectiveness against severe COVID-19 after partial mRNA and AdV immunization (**Figure S2**).

**Additional file 8. **The quality of evidence for factor-stratified outcomes (**Table S7**).

## Data Availability

The data that support the findings of this study are available from the corresponding author upon reasonable request.

## Abbreviations

AdV: Adenoviral vector vaccine
GRADE: Grading of Recommendations Assessment, Development, and Evaluation
HCWs: Healthcare workers
HRGs: High-risk groups
MOOSE: Meta-analysis Of Observational Studies in Epidemiology
NOS: Newcastle–Ottawa Quality Assessment Scale
PRISMA: Preferred Reporting Items for Systematic Review and Meta-Analyses
RoB: Risk of bias
VE: Vaccine effectiveness
