# Supplementary material for: Risk factors affecting COVID-19 vaccine effectiveness identified from 290 cross-country observational studies until February 2022: a meta-analysis and meta-regression
Source: BMC Med. 2022 Nov 25;20:461. doi: 10.1186/s12916-022-02663-z (PMC9701077; doi:10.1186/s12916-022-02663-z)
Supplement: Supplementary file 1 — Additional file 1. PRISMA-S Checklist (Table S1), PRISMA Checklist (Table S2), MOOSE Checklist (Table S3) [file 12916_2022_2663_MOESM1_ESM.docx]

**Additional file 1**

**Table S1. PRISMA-S Checklist**

| **Section/topic** | | **#** | | **Checklist item** | | **Location(s) Reported** |
| --- | --- | --- | --- | --- | --- | --- |
| **INFORMATION SOURCES AND METHODS** | | | | | | |
| Database name | 1 | | Name each individual database searched, stating the platform for each. | | See “Material and methods” section; MEDLINE (platform PubMed, STN International), EMBASE (STN International) and MedRxiv (free online archive) databases were searched. | |
| Multi-database searching | 2 | | If databases were searched simultaneously on a single platform, state the name of the platform, listing all of the databases searched. | | STN International: MEDLINE, EMBASE; PubMed: MEDLINE; free online archive of complete manuscript preprints deposited in server: MedRxiv | |
| Study registries | 3 | | List any study registries searched. | | Not applicable | |
| Online resources and browsing | 4 | | Describe any online or print source purposefully searched or browsed (e.g., tables of contents, print conference proceedings, web sites), and how this was done. | | To identify unpublished studies, we searched Embase for conference proceedings. | |
| Citation searching | 5 | | Indicate whether cited references or citing references were examined, and describe any methods used for locating cited/citing references (e.g., browsing reference lists, using a citation index, setting up email alerts for references citing included studies). | | Recursive search of references in published review or meta-analysis articles were manually screened to identify additional studies. | |
| Contacts | 6 | | Indicate whether additional studies or data were sought by contacting authors, experts, manufacturers, or other. | | Not done | |
| Other methods | 7 | | Describe any additional information sources or search methods used. | | Not done | |
| **SEARCH STRATEGIES** | | | | | | |
| Full search strategies | | 8 | | Include the search strategies for each database and information source, copied and pasted exactly as run. | | See “Material and methods” section; Table S4 – search strategy. |
| Limits and restrictions | | 9 | | Specify that no limits were used, or describe any limits or restrictions applied to a search (e.g., date or time period, language, study design) and provide justification for their use. | | We imposed no language or other restrictions on any of the searches. The inclusion criteria are defined in the “Material and Methods” section. Clinical trials were excluded. |
| Search filters | | 10 | | Indicate whether published search filters were used (as originally designed or modified), and if so, cite the filter(s) used. | | For our MEDLINE search, we created RSS feeds in PubMed with keyword "immunization" and "vaccine". |
| Prior work | | 11 | | Indicate when search strategies from other literature reviews were adapted or reused for a substantive part or all of the search, citing the previous review(s). | | Not applicable |
| Updates | | 12 | | Report the methods used to update the search(es) (e.g., rerunning searches, email alerts). | | PubMed RSS feeds were set up to provide daily updates of new literature. |
| Dates of searches | | 13 | | For each search strategy, provide the date when the last search occurred. | | A comprehensive literature search was initially run on 5 February and then rerun on 28 February 2022. |
| **PEER REVIEW** | | | | | | |
| Peer review | | 14 | | Describe any search peer review process. | | The strategies were peer reviewed by senior information specialist prior to execution. |
| **MANAGING RECORDS** | | | | | | |
| Total Records | | 15 | | Document the total number of records identified from each database and other information sources. | | “Results” section. A total of 14,155 publications were identified and retrieved from three databases. Flow chart – Figure 1. |
| Deduplication | | 16 | | Describe the processes and any software used to deduplicate records from multiple database searches and other information sources. | | Duplicates were removed by an information specialist (MF), using internal strategy of platform and then manually. Duplicates were manually revised after merging with literature obtained from MedRxiv. |
|  | | | | | | |

**Table S2. PRISMA Checklist**

| **Section and Topic** | **Item #** | **Checklist item** | **Location where item is reported** |
| --- | --- | --- | --- |
| **TITLE** | | |  |
| Title | 1 | Identify the report as a systematic review. | Title |
| **ABSTRACT** | | |  |
| Abstract | 2 | See the PRISMA 2020 for Abstracts checklist. | Abstract |
| **INTRODUCTION** | | |  |
| Rationale | 3 | Describe the rationale for the review in the context of existing knowledge. | Introduction |
| Objectives | 4 | Provide an explicit statement of the objective(s) or question(s) the review addresses. | Methods |
| **METHODS** | | |  |
| Eligibility criteria | 5 | Specify the inclusion and exclusion criteria for the review and how studies were grouped for the syntheses. | Methods |
| Information sources | 6 | Specify all databases, registers, websites, organisations, reference lists and other sources searched or consulted to identify studies. Specify the date when each source was last searched or consulted. | Methods, Supplement |
| Search strategy | 7 | Present the full search strategies for all databases, registers and websites, including any filters and limits used. | Methods, Supplement |
| Selection process | 8 | Specify the methods used to decide whether a study met the inclusion criteria of the review, including how many reviewers screened each record and each report retrieved, whether they worked independently, and if applicable, details of automation tools used in the process. | Methods, Supplement |
| Data collection process | 9 | Specify the methods used to collect data from reports, including how many reviewers collected data from each report, whether they worked independently, any processes for obtaining or confirming data from study investigators, and if applicable, details of automation tools used in the process. | Methods, Supplement, Contributors |
| Data items | 10a | List and define all outcomes for which data were sought. Specify whether all results that were compatible with each outcome domain in each study were sought (e.g. for all measures, time points, analyses), and if not, the methods used to decide which results to collect. | Methods, Supplement |
|  | 10b | List and define all other variables for which data were sought (e.g. participant and intervention characteristics, funding sources). Describe any assumptions made about any missing or unclear information. | Methods, Supplement |
| Study risk of bias assessment | 11 | Specify the methods used to assess risk of bias in the included studies, including details of the tool(s) used, how many reviewers assessed each study and whether they worked independently, and if applicable, details of automation tools used in the process. | Methods, Supplement |
| Effect measures | 12 | Specify for each outcome the effect measure(s) (e.g. risk ratio, mean difference) used in the synthesis or presentation of results. | Methods |
| Synthesis methods | 13a | Describe the processes used to decide which studies were eligible for each synthesis (e.g. tabulating the study intervention characteristics and comparing against the planned groups for each synthesis (item #5)). | Methods |
|  | 13b | Describe any methods required to prepare the data for presentation or synthesis, such as handling of missing summary statistics, or data conversions. | Methods |
|  | 13c | Describe any methods used to tabulate or visually display results of individual studies and syntheses. | Methods |
|  | 13d | Describe any methods used to synthesize results and provide a rationale for the choice(s). If meta-analysis was performed, describe the model(s), method(s) to identify the presence and extent of statistical heterogeneity, and software package(s) used. | Methods |
|  | 13e | Describe any methods used to explore possible causes of heterogeneity among study results (e.g. subgroup analysis, meta-regression). | Methods |
|  | 13f | Describe any sensitivity analyses conducted to assess robustness of the synthesized results. | Methods, Supplement |
| Reporting bias assessment | 14 | Describe any methods used to assess risk of bias due to missing results in a synthesis (arising from reporting biases). | Methods, Supplement |
| Certainty assessment | 15 | Describe any methods used to assess certainty (or confidence) in the body of evidence for an outcome. | Methods, Supplement |
| **RESULTS** | | |  |
| Study selection | 16a | Describe the results of the search and selection process, from the number of records identified in the search to the number of studies included in the review, ideally using a flow diagram. | Flowchart |
|  | 16b | Cite studies that might appear to meet the inclusion criteria, but which were excluded, and explain why they were excluded. | Not applicable |
| Study characteristics | 17 | Cite each included study and present its characteristics. | Supplement |
| Risk of bias in studies | 18 | Present assessments of risk of bias for each included study. | Supplement |
| Results of individual studies | 19 | For all outcomes, present, for each study: (a) summary statistics for each group (where appropriate) and (b) an effect estimate and its precision (e.g. confidence/credible interval), ideally using structured tables or plots. | Figures |
| Results of syntheses | 20a | For each synthesis, briefly summarise the characteristics and risk of bias among contributing studies. | Results, Supplement |
|  | 20b | Present results of all statistical syntheses conducted. If meta-analysis was done, present for each the summary estimate and its precision (e.g. confidence/credible interval) and measures of statistical heterogeneity. If comparing groups, describe the direction of the effect. | Results, Supplement |
|  | 20c | Present results of all investigations of possible causes of heterogeneity among study results. | Not applicable |
|  | 20d | Present results of all sensitivity analyses conducted to assess the robustness of the synthesized results. | Replaced by prediction intervals |
| Reporting biases | 21 | Present assessments of risk of bias due to missing results (arising from reporting biases) for each synthesis assessed. | Results, Supplement |
| Certainty of evidence | 22 | Present assessments of certainty (or confidence) in the body of evidence for each outcome assessed. | Results |
| **DISCUSSION** | | |  |
| Discussion | 23a | Provide a general interpretation of the results in the context of other evidence. | Discussion |
|  | 23b | Discuss any limitations of the evidence included in the review. | Discussion |
|  | 23c | Discuss any limitations of the review processes used. | Discussion |
|  | 23d | Discuss implications of the results for practice, policy, and future research. | Discussion, Conclusion |
| **OTHER INFORMATION** | | |  |
| Registration and protocol | 24a | Provide registration information for the review, including register name and registration number, or state that the review was not registered. | PROSPERO |
|  | 24b | Indicate where the review protocol can be accessed, or state that a protocol was not prepared. | Authors |
|  | 24c | Describe and explain any amendments to information provided at registration or in the protocol. | None |
| Support | 25 | Describe sources of financial or non-financial support for the review, and the role of the funders or sponsors in the review. | Cooperatio 31 fund and Ministry of Health of the Czech Republic, grant No. NU22-A-125. |
| Competing interests | 26 | Declare any competing interests of review authors. | Declaration |
| Availability of data, code and other materials | 27 | Report which of the following are publicly available and where they can be found: template data collection forms; data extracted from included studies; data used for all analyses; analytic code; any other materials used in the review. | Authors |

**Table S3. MOOSE Checklist**

| **Criteria** | **Brief description** |
| --- | --- |
| **Introduction** |  |
| Problem definition | Risk factors of COVID-19 vaccine effectiveness |
| Hypothesis statement | Null hypothesis: vaccine effectiveness >50% |
| A statement of objectives that includes the study population, condition of interest, exposure or intervention, and the outcome(s) considered | Study outcome: vaccine effectiveness  Study exposure: vaccination  Study population: general population classified by sex, age groups, concomitant diseases or increased risk |
| **Sources** |  |
| Qualifications of literature searchers (e.g., librarians and investigators) | The credential of the only literature researcher (MF) is available in the author team. |
| Search strategy including the time period required for the synthesis and keywords | The search strategy is detailed in Supplement, S-Table 3. |
| Effort to include all available studies, including contact with authors | References of all retrieved articles and recent reviews were reviewed. |
| Databases and registries searched | Medical Literature Analysis and Retrieval System Online (MEDLINE), Excerpta Medica dataBASE (EMBASE), MedRxiv database |
| Search software used, name and version, including special features used (e.g., explosion) | No special search software was used. |
| Use of hand searching (e.g., reference lists of eligible articles) | References of all retrieved articles and recent reviews were reviewed. |
| List of citations located and those excluded, including justification | Details of the literature search process are outlined in a flow chart (Figure 1) |
| Method of addressing articles published in languages other than English | No language restrictions. |
| Method of handling abstracts and unpublished studies | The search process was not restricted to peer-reviewed studies. |
| Description of any contact with authors | Not done. |
| **Study selection** |  |
| Types of study designs considered | Only observational studies: case-control, cohort studies or cross-over studies |
| Relevance or appropriateness of studies gathered for assessing the hypothesis to be tested | (1) observational studies; (2) exposure to immunization with any mRNA or adenoviral vector vaccine; (3) unvaccinated control group; and (4) outcome of interest determined by the effect size (ES) transformed to vaccine effectiveness: 100×(1–ES), including the 95% confidence interval. |
| Rationale for the selection and coding of data (e.g., sound clinical principles or convenience) | Study characteristics were extracted independently by two investigators (MP and IKL). Effect sizes (incl. 95% CI) of vaccinated versus unvaccinated participants were assessed separately whenever possible. In cases where a study reported more than one record of effect size (vaccine effectiveness), each size was related to a particular group according to the investigated factor. |
| Documentation of how data were classified and coded (e.g., multiple raters, blinding, and interrater reliability) | Data were independently extracted and analyzed by two investigators (MP and JD) with the final decision reached by consensus. Extracted data were placed into a unique database for subsequent arrangement and clustered. |
| Assessment of confounding (e.g., comparability of cases and controls in studies where appropriate) | The adjusted or matched-groups outcomes were prioritized. Moreover, risk of bias was assessed in all studies with NOS stars. |
| Assessment of study quality, including blinding of quality assessors: stratification or regression on possible predictors of study results | The quality of each study was assessed by two assessors (RM, ES, DJ, JD, SN and PD) using the Newcastle-Ottawa Quality Assessment Scale (NOS). NOS-based assessment was different for cohort and case-control studies.  Full comparability of the cohorts or cases and controls was achieved if they matched in sex and age as well as in at least another arbitrary parameter. Adequacy of cohort follow-up as well as the same non-response rates of cases and controls were accepted if not exceeding a 20% difference. |
| Assessment of heterogeneity | The Q-statistic and I-squared statistic were used to assess the heterogeneity of studies. |
| Statistical methods (e.g., complete description of fixed- or random-effects models, justification of whether the chosen models account for predictors of study results, dose-response models, or cumulative meta-analysis) in sufficient detail to be replicated | Description of the methods of meta-analyses/meta-regression, subgroup analyses, and assessment of publication bias are detailed in the “Statistical methods” and “Results” sections. |
| **Results** |  |
| A graph summarizing individual study estimates and the overall estimate | A summary chart of subtotal results in groups of post-vaccination time is incorporated in the main body of text (Figures 2, 3 and 4). Conservative forest plots cannot be displayed because of the high number of effectiveness records. One main table (Table 1) is provided. |
| A table giving descriptive information for each study included | S-Table 5 |
| Results of sensitivity testing (e.g., subgroup analysis) | See “Results” section and Figures 2, 3 and 4, S-Figures 1 and 2. |
| Indication of statistical uncertainty of findings | 95% confidence intervals are provided with all summary effect estimates. |
| **Discussion** |  |
| Strengths and weaknesses | The strength of evidence; assessment of RoB; additional analyzes of effect of small and unpublished studies were performed. Publication bias was tested using outcomes of both models of random- and fixed-effects. |
| Potential biases in the review process (e.g., publication bias) | See “Statistical Methods”, “Results” and “Discussion” sections. |
| Justification for exclusion (e.g., exclusion of non-English-language citations) | See “Material and methods” section (inclusion criteria) |
| Assessment of quality of included studies | See “Results” section (Quality of evidence) |
| Consideration of alternative explanations for observed results | The outcome of booster immunization must be interpreted with caution given the limited number of studies. |
| Generalization of the conclusions (i.e., appropriate for the data presented and within the domain of the literature review) | The summary outcome can be generally accepted because criteria supporting the strength of evidence were met. |
| Guidelines for future research | We underlined that our findings cannot be regarded as conclusive for booster immunization. |
| Disclosure of funding source | This work was supported by the Cooperatio 31 fund, Health Sciences, Charles University, Prague, Czech Republic, and Ministry of Health of the Czech Republic, grant No. NU22-A-125. |
|  |  |
