# Supplementary material for: Risk factors affecting COVID-19 vaccine effectiveness identified from 290 cross-country observational studies until February 2022: a meta-analysis and meta-regression
Source: BMC Med. 2022 Nov 25;20:461. doi: 10.1186/s12916-022-02663-z (PMC9701077; doi:10.1186/s12916-022-02663-z)
Supplement: Supplementary file 2 — Additional file 2. Search strategy (Table S4). [file 12916_2022_2663_MOESM2_ESM.docx]

**Additional file 2**

**Table S4. Search strategy**

| **Criteria** |
| --- |
| Search query: Medline + Embase  1) Vaccine  **VAC1** *VACCIN* OR *IMMUNIS* OR *IMMUNIZ*  **VAC2** CHADOX1 OR BNT162* OR AZD1222 OR AD26COVS1 OR BNT162B2 OR MRNA(W)1273  **VAC3** "VACCINATION"/CT OR "VACCINE"/CT OR "VACCINES"/CT OR "MRNA VACCINES"/CT OR "VIRAL VACCINES"/CT OR "MASS VACCINATION"/CT OR "IMMUNIZATION"/CT OR "IMMUNIZATION PROGRAMS"/CT OR "IMMUNIZATION, SECONDARY"/CT  **VAC4** "COVID-19 VACCINES"/CT OR "2019-NCOV VACCINE MRNA-1273"/CT OR "BNT162 VACCINE"/CT OR "AD26COVS1"/CT OR "CHADOX1 NCOV-19"/CT  2) COVID-19  **COV1** COVID* OR NCOV* OR SARS2 OR SARSCOV2 OR 2019NCOV OR (SEVERE(W)ACUTE(W)RESPIRATORY(W)SYNDROME OR SARS)(W)(COV OR CORONA*)(W)2 OR CORONA*(2A)(EPIDEMIC OR OUTBREAK OR PANDEMIC)  **COV2** (2019 OR 19)(A)(NCOV OR HCOV OR CORONAVIRUS OR CORONA(W)VIRUS OR SARS) OR (NEW OR NOVEL OR WUHAN OR 2)(A)(CORONAVIRUS* OR SARS OR CORONA(W)VIRUS)  **COV3** "SARS-COV-2"/CT OR "COVID-19"/CT OR "CORONAVIRUS INFECTIONS"/CT OR "CORONAVIRUS DISEASE 2019"/CT OR "CORONAVIRUS"/CT  3) Effectiveness  **EFF1** "*COMPARATIVE EFFECTIVENESS RESEARCH"/CT  **EFF2** "VACCINE EFFICACY"/CT  **EFF3** EFFECTIVENESS OR "REAL-WORLD" OR "VACCINE EFFECTIVENESS" OR "REAL-WORLD DATA" OR "REAL-WORLD EVIDENCE" OR "REAL-WORLD INVESTIGATION" OR "REAL-WORLD OUTCOMES"  **EFF4** (EFFECT* OR IMPACT)(5A)(*VACCIN* OR *IMMUNIS* OR *IMMUNIZ* OR CHADOX1 OR BNT162* OR AZD1222 OR AD26COVS1 OR BNT162B2 OR MRNA(W)1273)  Medline  **1A (VAC1 OR VAC2) AND (COV1 OR COV2) AND (EFF1 OR EFF2 OR EFF3 OR EFF4)**  **1B (VAC4 OR ((VAC3 OR VAC1/TI) AND (COV3 OR COV1/TI OR COV2/TI))) AND (EFF1 OR EFF2 OR EFF3 or EFF4)**  **(1A OR 1B) NOT ANIMAL/CT**  Embase  **VAC5** "*SARS-COV-2 VACCINE"/CT OR "*SEVERE ACUTE RESPIRATORY SYNDROME VACCINE"/CT  **VAC6** "*AD26.COV2.S VACCINE"/CT OR "*COMIRNATY"/CT OR "*CORONAVAC"/CT OR "*COMIRNATY"/CT OR "*MRNA-1273 VACCINE"/CT OR "*RNA VACCINE"/CT OR "*SPUTNIK V VACCINE"/CT OR "*MRNA 1273"/CT  **VAC7** "*VACCINE"/CT OR "*VACCINATION"/CT OR "*IMMUNIZATION"/CT OR "*MASS IMMUNIZATION"/CT OR "*RNA IMMUNIZATION"/CT  **COV4** "*CORONAVIRUS DISEASE 2019"/CT  **EFF5** "CLINICAL EFFECTIVENESS"/CT OR "COMPARATIVE EFFECTIVENESS"/CT OR "DRUG EFFECT"/CT OR "PROGRAM EFFECTIVENESS"/CT  **EFF6** "VACCINE EFFECTIVENESS"/ST OR EFFECTIVENESS/ST OR "EFFECTIVENESS OF THE SARS-COV-2 VACCINE"/ST OR "REAL-WORLD"/ST OR "COMPARATIVE EFFECTIVENESS"/CT OR "*DRUG EFFECT"/CT  **EFF7** "POST VACCINE SURVEILLANCE"/ST  **2A (VAC1 OR VAC2) AND (COV1 OR COV2) AND (EFF1 OR EFF2 OR EFF3 OR EFF4)**  **2B (VAC5 OR VAC6 OR (VAC7 AND COV4)) AND (EFF3 OR EFF4 OR EFF5 OR EFF6 OR EFF7 OR EFFECTIVENESS/TI)**  **(2A OR 2B) AND HUMAN/CT**  Search query: MedRxiv  (COVID* OR SARS*) AND (VACCIN* or IMMUNIS* OR IMMUNIZ*) AND effectiveness |
|  |
