# Supplementary material for: Risk factors affecting COVID-19 vaccine effectiveness identified from 290 cross-country observational studies until February 2022: a meta-analysis and meta-regression
Source: BMC Med. 2022 Nov 25;20:461. doi: 10.1186/s12916-022-02663-z (PMC9701077; doi:10.1186/s12916-022-02663-z)
Supplement: Supplementary file 3 — Additional file 3. Data extraction, assessment of evidence quality, assessment of RoB. [file 12916_2022_2663_MOESM3_ESM.docx]

**Additional file 3**

**Data extraction**

The following data were extracted by two reviewers:

1. Study design (cohort study, case-control study, or cross-sectional study);
2. Start and end dates of the observational study;
3. Study characteristics (title, 1st author name and year of publication, including country of study population);
4. Specification of the population (comorbidities and type of study population such as healthcare workers, those living in long-term care or residential care homes, etc.);
5. Total number of participants, number of men, number of vaccinated participants;
6. Age of the study population characterized by any of the following parameters: mean age including standard deviation; or median age including interquartile range; minimum and, if applicable, maximum age;
7. Confirmation of COVID-19 regardless of its severity by PCR (molecular test), antigen test, or serology;
8. The follow-up period was evaluated at least by the interval between start and end study, unless the authors explicitly stated the mean or median of follow-up;
9. Circulating SAR-CoV-2 variants, if the outcome was related to a variant specified by the authors themselves;
10. Analysis, including the method used and adjustment or matching;
11. Interventions (commercial or type-specific vaccine, number of doses);
12. Outcomes (vaccine effectiveness against SARS-CoV-2 infection independently of symptom presence, against COVID-19 regardless of its severity, against hospitalization for and death from COVID-19, related to specific variants and post-vaccination time).

Data of each study was extracted by 2 reviewers. If consensus had not been achieved, the discrepancy was resolved through discussions among reviewers.

**Assessment of evidence quality**

The following criteria were adopted to assess the strength of evidence according to GRADE:

1. A sufficient number of VE records (≥10);
2. No serious limitation was assumed in studies at low risk of bias (RoB) having ≥7 NOS stars;
3. No serious heterogeneity of studies was achieved if pooled inconsistency index <50%;
4. No serious indirectness based on difference between vaccinated and unvaccinated participants was documented in studies having 2 stars of NOS comparability of groups;
5. No serious imprecision of studies was observed if pooled standard error (SE) <10%;
6. No serious publication bias of studies was documented if no significant difference between log-transformed effect sizes derived from VEs of both models, i.e., fixed-effect and random-effects models.

**Assessment of RoB (risk of bias) according to NOS (Newcastle–Ottawa Quality Assessment Scale) stars**

Cohort studies and case-control studies were evaluated according to the respective NOS questionnaire. A few studies were conducted as cross-sectional ones and were evaluated using the questionnaire for cohort studies. A modified questionnaire for cross-sectional studies was not intentionally used as it did not fully meet the needs for these specific studies focused on vaccine effectiveness. This procedure maintained consistency for the final evaluation of all observational studies.

While both types of studies evaluated selection and comparability, outcome (disease) and exposure (vaccination) were assessed by cohort and case-control studies, respectively. For the purpose of this study, the following queries were adapted:

1) "Demonstration that outcome of interest was not present at start of study" or "Definition of Controls - no history of disease (endpoint)"

Note: If the study authors stated that they had excluded previously infected participants from the analysis or that they had conducted the analysis with adjustment to a previous infection, then the query was awarded a star.

2) Comparability of cohorts (or cases and controls) on the basis of design or analysis

a) Study controls for _____________ (select the most important factor)

b) Study controls for any additional factor

a) The most important selected factors were sex and age.

b) An additional factor in addition to sex and age was required.

3) Was follow-up long enough for outcomes to occur

The answer was "yes" if the study follow-up was at least 4 weeks (1 month).

4) Adequacy of follow-up of cohorts

Subjects lost to follow-up unlikely to introduce bias – small number lost (<20%)

5) Non-Response rate

The same rate for both groups was if the rate of cases was not different from that of controls by more than 20%.

As the assessment of RoB was performed independently by a total of 6 assessors, a standard operating procedure had been developed. Each study was evaluated by 2 assessors. If consensus had not been achieved, the discrepancy was resolved through discussions among all assessors.
