# Supplementary material for: Risk factors affecting COVID-19 vaccine effectiveness identified from 290 cross-country observational studies until February 2022: a meta-analysis and meta-regression
Source: BMC Med. 2022 Nov 25;20:461. doi: 10.1186/s12916-022-02663-z (PMC9701077; doi:10.1186/s12916-022-02663-z)
Supplement: Supplementary file 4 — Additional file 4. Studies included in quantitative synthesis (Table S5). [file 12916_2022_2663_MOESM4_ESM.docx]

**Additional file 4**

**Table S5. Studies included in quantitative synthesis**

| **Study** |  |
| --- | --- |
| Abhilash, 2022 | Abhilash KPP, Mathiyalagan P, Krishnaraj VRK, Selvan S, Kanagarajan R, Reddy NP, Rajendiran N, Hazra D, Gunasekaran K, Moorthy M, Jasmine S, Davis JP, George T, George K, Varghese GM, Rupali P, Barney Isaac TJ, Gupta R, Pichamuthu K, Joy M, Jayaseelan L, Mathews P, Peter JV. Impact of prior vaccination with Covishield™ and Covaxin^®^ on mortality among symptomatic COVID-19 patients during the second wave of the pandemic in South India during April and May 2021: a cohort study. Vaccine. 2022 Feb 10;40(13):2107–13. doi: 10.1016/j.vaccine.2022.02.023. Epub ahead of print. PMID: 35168837; PMCID: PMC8828422. |
| Abu-Raddad, 2022 | Abu-Raddad LJ, Chemaitelly H, Bertollini R; National Study Group for COVID-19 Vaccination. Waning mRNA-1273 Vaccine Effectiveness against SARS-CoV-2 Infection in Qatar. N Engl J Med. 2022 Jan 26. doi: 10.1056/NEJMc2119432. Epub ahead of print. PMID: 35081294. |
| Abu-Raddad, 2021 | Abu-Raddad LJ, Chemaitelly H, Butt AA; National Study Group for COVID-19 Vaccination. Effectiveness of the BNT162b2 Covid-19 Vaccine against the B.1.1.7 and B.1.351 Variants. N Engl J Med. 2021 Jul 8;385(2):187-189. doi: 10.1056/NEJMc2104974. Epub 2021 May 5. PMID: 33951357; PMCID: PMC8117967. |
| Accorsi, 2022 | Accorsi EK, Britton A, Fleming-Dutra KE, Smith ZR, Shang N, Derado G, Miller J, Schrag SJ, Verani JR. Association Between 3 Doses of mRNA COVID-19 Vaccine and Symptomatic Infection Caused by the SARS-CoV-2 Omicron and Delta Variants. JAMA. 2022 Jan 21. doi: 10.1001/jama.2022.0470. Epub ahead of print. PMID: 35060999. |
| Alali, 2021 | Alali WQ, Ali LA, AlSeaidan M, Al-Rashidi M. Effectiveness of BNT162b2 and ChAdOx1 Vaccines against Symptomatic COVID-19 among Healthcare Workers in Kuwait: A Retrospective Cohort Study. Healthcare (Basel). 2021 Dec 7;9(12):1692. doi: 10.3390/healthcare9121692. PMID: 34946418; PMCID: PMC8701668. |
| Alencar, 2021 | Alencar CH, Cavalcanti LPG, Almeida MM, Barbosa PPL, Cavalcante KKS, Melo DN, de Brito Alves BCF, Heukelbach J. High Effectiveness of SARS-CoV-2 Vaccines in Reducing COVID-19-Related Deaths in over 75-Year-Olds, Ceará State, Brazil. Trop Med Infect Dis. 2021 Jul 13;6(3):129. doi: 10.3390/tropicalmed6030129. PMID: 34287384; PMCID: PMC8293450. |
| Amirthalingam, 2021 | Amirthalingam G, Bernal JL, Andrews NJ, Whitaker H, Gower C, Stowe J, Tessier E, Subbarao S, Ireland G, Baawuah F, Linley E, Warrener L, O'Brien M, Whillock C, Moss P, Ladhani SN, Brown KE, Ramsay ME. Serological responses and vaccine effectiveness for extended COVID-19 vaccine schedules in England. Nat Commun. 2021 Dec 10;12(1):7217. doi: 10.1038/s41467-021-27410-5. PMID: 34893611; PMCID: PMC8664823. |
| Amit, 2021 | Amit S, Regev-Yochay G, Afek A, Kreiss Y, Leshem E. Early rate reductions of SARS-CoV-2 infection and COVID-19 in BNT162b2 vaccine recipients. Lancet. 2021 Mar 6;397(10277):875-877. doi: 10.1016/S0140-6736(21)00448-7. Epub 2021 Feb 18. PMID: 33610193; PMCID: PMC7906709. |
| Andeweg, 2022 | Stijn P. Andeweg, Brechje de Gier, Dirk Eggink, Caroline van den Ende, Noortje van Maarseveen, Lubna Ali, Boris Vlaemynck, Raf Schepers, RIVM COVID-19 surveillance and epidemiology team, Susan J.M. Hahné, Chantal Reusken, Hester E. de Melker, Susan van den Hof, Mirjam J. Knol. Protection of COVID-19 vaccination and previous infection against Omicron BA.1 and Delta SARS-CoV-2 infections, the Netherlands, 22 November 2021- 19 January 2022. medRxiv 2022.02.06.22270457; doi: https://doi.org/10.1101/2022.02.06.22270457 |
| Andrejko, 2021 | Andrejko KL, Pry J, Myers JF, Jewell NP, Openshaw J, Watt J, Jain S, Lewnard JA; California COVID-19 Case-Control Study Team. Prevention of COVID-19 by mRNA-based vaccines within the general population of California. Clin Infect Dis. 2021 Jul 20:ciab640. doi: 10.1093/cid/ciab640. Epub ahead of print. PMID: 34282839; PMCID: PMC8406879. |
| Andrews, 2022 | Andrews N, Tessier E, Stowe J, Gower C, Kirsebom F, Simmons R, Gallagher E, Thelwall S, Groves N, Dabrera G, Myers R, Campbell CNJ, Amirthalingam G, Edmunds M, Zambon M, Brown K, Hopkins S, Chand M, Ladhani SN, Ramsay M, Lopez Bernal J. Duration of Protection against Mild and Severe Disease by Covid-19 Vaccines. N Engl J Med. 2022 Jan 12. doi: 10.1056/NEJMoa2115481. Epub ahead of print. PMID: 35021002. |
| Andrews, 2022 | Andrews N, Stowe J, Kirsebom F, Toffa S, Sachdeva R, Gower C, Ramsay M, Bernal JL. Effectiveness of COVID-19 booster vaccines against covid-19 related symptoms, hospitalisation and death in England. Nat Med. 2022 Jan 14. doi: 10.1038/s41591-022-01699-1. Epub ahead of print. PMID: 35045566. |
| Andrews, 2022 | N. Andrews, J. Stowe, F. Kirsebom, S. Toffa, T. Rickeard, E. Gallagher, C. Gower, M. Kall, N. Groves, A.-M. O’Connell, D. Simons, P.B. Blomquist, A. Zaidi, S. Nash, N. Iwani Binti Abdul Aziz, S. Thelwall, G. Dabrera, R. Myers, G. Amirthalingam, S. Gharbia, J.C. Barrett, R. Elson, S.N. Ladhani, N. Ferguson, M. Zambon, C.N.J. Campbell, K. Brown, S. Hopkins, M. Chand, M. Ramsay, and J. Lopez Bernal. Covid-19 Vaccine Effectiveness against the Omicron (B.1.1.529) Variant. 2022. DOI: 10.1056/NEJMoa2119451 |
| Angel, 2021 | Angel Y, Spitzer A, Henig O, Saiag E, Sprecher E, Padova H, Ben-Ami R. Association Between Vaccination With BNT162b2 and Incidence of Symptomatic and Asymptomatic SARS-CoV-2 Infections Among Health Care Workers. JAMA. 2021 Jun 22;325(24):2457-2465. doi: 10.1001/jama.2021.7152. PMID: 33956048; PMCID: PMC8220476. |
| Aslam , 2022 | Aslam S, Liu J, Sigler R, Syed RR, Tu XM, Little SJ, De Gruttola V. COVID-19 vaccination is protective of clinical disease in solid organ transplant recipients. Transpl Infect Dis. 2022 Jan 5. doi: 10.1111/tid.13788. Epub ahead of print. PMID: 34989104. |
| Aslam, 2021 | Aslam S, Adler E, Mekeel K, Little SJ. Clinical effectiveness of COVID-19 vaccination in solid organ transplant recipients. Transpl Infect Dis. 2021 Oct;23(5):e13705. doi: 10.1111/tid.13705. Epub 2021 Aug 3. PMID: 34324256; PMCID: PMC8420394. |
| Auvigne, 2022 | Vincent Auvigne, Sophie Vaux, Yann Le Strat, Justine Schaeffer, Lucie Fournier, Cynthia Tamandjou, Charline Montagnat, Bruno Coignard, Daniel Levy-Bruhl, Isabelle Parent du Châtelet. Severe hospital events following symptomatic infection with Sars-CoV-2 Omicron and Delta variants in France, December 2021 – January 2022: a retrospective, population-based, matched cohort study. medRxiv 2022.02.02.22269952; doi: https://doi.org/10.1101/2022.02.02.22269952 |
| Azamgarhi, 2021 | Azamgarhi T, Hodgkinson M, Shah A, Skinner JA, Hauptmannova I, Briggs TWR, Warren S. BNT162b2 vaccine uptake and effectiveness in UK healthcare workers - a single centre cohort study. Nat Commun. 2021 Jun 17;12(1):3698. doi: 10.1038/s41467-021-23927-x. PMID: 34140469; PMCID: PMC8211683. |
| Bajema, 2021 | Bajema KL, Dahl RM, Evener SL, Prill MM, Rodriguez-Barradas MC, Marconi VC, Beenhouwer DO, Holodniy M, Lucero-Obusan C, Brown ST, Tremarelli M, Epperson M, Mills L, Park SH, Rivera-Dominguez G, Morones RG, Ahmadi-Izadi G, Deovic R, Mendoza C, Jeong C, Schrag SJ, Meites E, Hall AJ, Kobayashi M, McMorrow M, Verani JR, Thornburg NJ, Surie D; SUPERNOVA COVID-19; Surveillance Group; Surveillance Platform for Enteric and Respiratory Infectious Organisms at the VA (SUPERNOVA) COVID-19 Surveillance Group. Comparative Effectiveness and Antibody Responses to Moderna and Pfizer-BioNTech COVID-19 Vaccines among Hospitalized Veterans - Five Veterans Affairs Medical Centers, United States, February 1-September 30, 2021. MMWR Morb Mortal Wkly Rep. 2021 Dec 10;70(49):1700-1705. doi: 10.15585/mmwr.mm7049a2. PMID: 34882654; PMCID: PMC8659185. |
| Bajema, 2021 | Bajema KL, Dahl RM, Prill MM, Meites E, Rodriguez-Barradas MC, Marconi VC, Beenhouwer DO, Brown ST, Holodniy M, Lucero-Obusan C, Rivera-Dominguez G, Morones RG, Whitmire A, Goldin EB, Evener SL, Tremarelli M, Tong S, Hall AJ, Schrag SJ, McMorrow M, Kobayashi M, Verani JR, Surie D; SUPERNOVA COVID-19; Surveillance Group; Surveillance Platform for Enteric and Respiratory Infectious Organisms at the VA (SUPERNOVA) COVID-19 Surveillance Group. Effectiveness of COVID-19 mRNA Vaccines Against COVID-19-Associated Hospitalization - Five Veterans Affairs Medical Centers, United States, February 1-August 6, 2021. MMWR Morb Mortal Wkly Rep. 2021 Sep 17;70(37):1294-1299. doi: 10.15585/mmwr.mm7037e3. PMID: 34529636; PMCID: PMC8445376. |
| Baltas, 2021 | Baltas I, Boshier FAT, Williams CA, Bayzid N, Cotic M, Guerra-Assunção JA, Irish-Tavares D, Haque T, Hart J, Roy S, Williams R, Breuer J, Mahungu TW. Post-vaccination COVID-19: A case-control study and genomic analysis of 119 breakthrough infections in partially vaccinated individuals. Clin Infect Dis. 2021 Aug 19:ciab714. doi: 10.1093/cid/ciab714. Epub ahead of print. PMID: 34410361; PMCID: PMC8513403. |
| Barchuk, 2021 | Anton Barchuk, Mikhail Cherkashin, Anna Bulina, Natalia Berezina, Tatyana Rakova, Darya Kuplevatskaya, Oksana Stanevich, Dmitriy Skougarevskiy, Artemiy Okhotin.Vaccine Effectiveness against Referral to Hospital and Severe Lung Injury Associated with COVID-19: A Population-Based Case-Control Study in St. Petersburg, Russia. medRxiv 2021.08.18.21262065; doi: https://doi.org/10.1101/2021.08.18.21262065 |
| Barchuk, 2022 | Anton Barchuk, Anna Bulina, Mikhail Cherkashin, Natalia Berezina, Tatyana Rakova, Darya Kuplevatskaya, Oksana Stanevich, Dmitriy Skougarevskiy, Artemiy Okhotin. COVID-19 vaccines effectiveness against symptomatic SARS-CoV-2 Delta variant infection: a population-based case-control study in St. Petersburg, Russia. medRxiv 2022.01.24.22269714; doi: https://doi.org/10.1101/2022.01.24.22269714 |
| Barlow, 2021 | Russell S. Barlow, Kevin Jian, Lindsey Larson. Effectiveness of COVID-19 Vaccines Against SARS-CoV-2 Infection During a Delta Variant Epidemic Surge in Multnomah County, Oregon, July 2021. medRxiv 2021.08.30.21262446; doi: https://doi.org/10.1101/2021.08.30.21262446 |
| Baum, 2021 | Baum U, Poukka E, Palmu AA, Salo H, Lehtonen TO, Leino T. Effectiveness of vaccination against SARS-CoV-2 infection and Covid-19 hospitalisation among Finnish elderly and chronically ill – An interim analysis of a nationwide cohort study. PLoS One. 2021 Nov 18;16(11):e0258704. doi: 10.1371/journal.pone.0258704. PMID: 34793476; PMCID: PMC8601574. |
| Bedston, 2022 | Bedston S, Akbari A, Jarvis CI, Lowthian E, Torabi F, North L, Lyons J, Perry M, Griffiths LJ, Owen RK, Beggs J, Chuter A, Bradley DT, de Lusignan S, Fry R, Richard Hobbs FD, Hollinghurst J, Katikireddi SV, Murphy S, O’Reily D, Robertson C, Shi T, Tsang RSM, Sheikh A, Lyons RA. COVID-19 vaccine uptake, effectiveness, and waning in 82,959 health care workers: A national prospective cohort study in Wales. Vaccine. 2022 Jan 15:S0264-410X(21)01535-8. doi: 10.1016/j.vaccine.2021.11.061. Epub ahead of print. PMID: 35042645; PMCID: PMC8760602. |
| Bell, 2022 | Bell S, Campbell J, Lambourg E, Watters C, O’Neil M, Almond A, Buck K, Carr EJ, Clark L, Cousland Z, Findlay M, Joss N, Metcalfe W, Petrie M, Spalding E, Traynor JP, Sanu V, Thomson P, Methven S, Mark PB. The Impact of Vaccination on Incidence and Outcomes of SARS-CoV-2 Infection in Patients with Kidney Failure in Scotland. J Am Soc Nephrol. 2022 Feb 2:ASN.2022010046. doi: 10.1681/ASN.2022010046. Epub ahead of print. PMID: 35110363. |
| Berec, 2021 | Luděk Berec, Martin Šmíd, Lenka Přibylová, Ondřej Májek, Tomáš Pavlík, Jiří Jarkovský, Milan Zajíček, Jakub Weiner, Tamara Barusová, Jan Trnka. Real-life protection provided by vaccination, booster doses and previous infection against covid-19 infection, hospitalisation or death over time in the Czech Republic: a whole country retrospective view. medRxiv 2021.12.10.21267590; doi: https://doi.org/10.1101/2021.12.10.21267590 |
| Bermingham, 2021 | Charlotte Bermingham, Jasper Morgan, Daniel Ayoubkhani, Myer Glickman, Nazrul Islam, Aziz Sheikh, Jonathan Sterne, A. Sarah Walker, Vahé Nafilyan. Estimating the effectiveness of first dose of COVID-19 vaccine against mortality in England: a quasi-experimental study. medRxiv 2021.07.12.21260385; doi: https://doi.org/10.1101/2021.07.12.21260385 |
| Bertollini, 2021 | Bertollini R, Chemaitelly H, Yassine HM, Al-Thani MH, Al-Khal A, Abu-Raddad LJ. Associations of Vaccination and of Prior Infection With Positive PCR Test Results for SARS-CoV-2 in Airline Passengers Arriving in Qatar. JAMA. 2021 Jul 13;326(2):185-188. doi: 10.1001/jama.2021.9970. PMID: 34106201; PMCID: PMC8190701. |
| Bianchi, 2021 | Bianchi FP, Germinario CA, Migliore G, Vimercati L, Martinelli A, Lobifaro A, Tafuri S, Stefanizzi P; Control Room Working Group. BNT162b2 mRNA COVID-19 Vaccine Effectiveness in the Prevention of SARS-CoV-2 Infection: A Preliminary Report. J Infect Dis. 2021 Aug 2;224(3):431-434. doi: 10.1093/infdis/jiab262. PMID: 34007998; PMCID: PMC8194590. |
| Bianchi, 2021 | Bianchi FP, Tafuri S, Migliore G, Vimercati L, Martinelli A, Lobifaro A, Diella G, Stefanizzi P, Group OBOTCRW. BNT162b2 mRNA COVID-19 Vaccine Effectiveness in the Prevention of SARS-CoV-2 Infection and Symptomatic Disease in Five-Month Follow-Up: A Retrospective Cohort Study. Vaccines (Basel). 2021 Oct 7;9(10):1143. doi: 10.3390/vaccines9101143. PMID: 34696252; PMCID: PMC8538139. |
| Björk, 2021 | Björk J, Inghammar M, Moghaddassi M, Rasmussen M, Malmqvist U, Kahn F. High level of protection against COVID-19 after two doses of BNT162b2 vaccine in the working age population - first results from a cohort study in Southern Sweden. Infect Dis (Lond). 2022 Feb;54(2):128-133. doi: 10.1080/23744235.2021.1982144. Epub 2021 Sep 29. PMID: 34586934; PMCID: PMC8500302. |
| Björk, 2021 | Jonas Björk, Carl Bonander, Mahnaz Moghaddassi, Magnus Rasmussen, Ulf Malmqvist, Fredrik Kahn, Malin Inghammar. Surveillance of COVID-19 vaccine effectiveness – a real-time case-control study in southern Sweden. medRxiv 2021.12.09.21267515; doi: https://doi.org/10.1101/2021.12.09.21267515 |
| Bobdey, 2021 | Bobdey S, Kaushik SK, Sahu R, Naithani N, Vaidya R, Sharma M, Vashishtha K, Yadav AK, Sen S, Karade S. Effectiveness of ChAdOx1 nCOV-19 Vaccine: Experience of a tertiary care institute. Med J Armed Forces India. 2021 Jul;77(Suppl 2):S271-S277. doi: 10.1016/j.mjafi.2021.06.006. Epub 2021 Jul 26. PMID: 34334893; PMCID: PMC8313083. |
| Bouton, 2021 | Bouton TC, Lodi S, Turcinovic J, Schaeffer B, Weber SE, Quinn E, Korn C, Steiner J, Schechter-Perkins EM, Duffy E, Ragan EJ, Taylor BP, Miller N, Davidoff R, Hanage WP, Connor J, Pierre C, Jacobson KR. Coronavirus Disease 2019 Vaccine Impact on Rates of Severe Acute Respiratory Syndrome Coronavirus 2 Cases and Postvaccination Strain Sequences Among Health Care Workers at an Urban Academic Medical Center: A Prospective Cohort Study. Open Forum Infect Dis. 2021 Sep 17;8(10):ofab465. doi: 10.1093/ofid/ofab465. PMID: 34646910; PMCID: PMC8500299. |
| Braeye, 2021 | Braeye T, Cornelissen L, Catteau L, Haarhuis F, Proesmans K, De Ridder K, Djiena A, Mahieu R, De Leeuw F, Dreuw A, Hammami N, Quoilin S, Van Oyen H, Wyndham-Thomas C, Van Cauteren D. Vaccine effectiveness against infection and onwards transmission of COVID-19: Analysis of Belgian contact tracing data, January-June 2021. Vaccine. 2021 Sep 15;39(39):5456-5460. doi: 10.1016/j.vaccine.2021.08.060. Epub 2021 Aug 19. PMID: 34454789; PMCID: PMC8373820. |
| Britton, 2021 | Britton A, Jacobs Slifka KM, Edens C, Nanduri SA, Bart SM, Shang N, Harizaj A, Armstrong J, Xu K, Ehrlich HY, Soda E, Derado G, Verani JR, Schrag SJ, Jernigan JA, Leung VH, Parikh S. Effectiveness of the Pfizer-BioNTech COVID-19 Vaccine Among Residents of Two Skilled Nursing Facilities Experiencing COVID-19 Outbreaks - Connecticut, December 2020-February 2021. MMWR Morb Mortal Wkly Rep. 2021 Mar 19;70(11):396-401. doi: 10.15585/mmwr.mm7011e3. PMID: 33735160; PMCID: PMC7976620. |
| Britton, 2022 | Britton A, Fleming-Dutra KE, Shang N, Smith ZR, Dorji T, Derado G, Accorsi EK, Ajani UA, Miller J, Schrag SJ, Verani JR. Association of COVID-19 Vaccination With Symptomatic SARS-CoV-2 Infection by Time Since Vaccination and Delta Variant Predominance. JAMA. 2022 Feb 14. doi: 10.1001/jama.2022.2068. Epub ahead of print. PMID: 35157002. |
| Bruxvoort, 2021 | Bruxvoort KJ, Sy LS, Qian L, Ackerson BK, Luo Y, Lee GS, Tian Y, Florea A, Aragones M, Tubert JE, Takhar HS, Ku JH, Paila YD, Talarico CA, Tseng HF. Effectiveness of mRNA-1273 against delta, mu, and other emerging variants of SARS-CoV-2: test negative case-control study. BMJ. 2021 Dec 15;375:e068848. doi: 10.1136/bmj-2021-068848. PMID: 34911691; PMCID: PMC8671836. |
| Bruxvoort, 2022 | Bruxvoort KJ, Sy LS, Qian L, Ackerson BK, Luo Y, Lee GS, Tian Y, Florea A, Takhar HS, Tubert JE, Talarico CA, Tseng HF. Real-world effectiveness of the mRNA-1273 vaccine against COVID-19: Interim results from a prospective observational cohort study. Lancet Reg Health Am. 2022 Feb;6:100134. doi: 10.1016/j.lana.2021.100134. Epub 2021 Nov 25. PMID: 34849505; PMCID: PMC8614600. |
| Buchan, 2022 | Sarah A. Buchan, Hannah Chung, Kevin A. Brown, Peter C. Austin, Deshayne B. Fell, Jonathan B. Gubbay, Sharifa Nasreen, Kevin L. Schwartz, Maria E. Sundaram, Mina Tadrous, Kumanan Wilson, Sarah E. Wilson, Jeffrey C. Kwong. Effectiveness of COVID-19 vaccines against Omicron or Delta symptomatic infection and severe outcomes. medRxiv 2021.12.30.21268565; doi: https://doi.org/10.1101/2021.12.30.21268565 |
| Busic, 2022 | Busic N, Lucijanic T, Barsic B, Luksic I, Busic I, Kurdija G, Barbic L, Kunstek S, Jelic T, Lucijanic M. Vaccination provides protection from respiratory deterioration and death among hospitalized COVID-19 patients: differences between vector and mRNA vaccines. J Med Virol. 2022 Feb 20. doi: 10.1002/jmv.27666. Epub ahead of print. PMID: 35187697. |
| Butt, 2021 | Butt AA, Nafady-Hego H, Chemaitelly H, Abou-Samra AB, Khal AA, Coyle PV, Kanaani ZA, Kaleeckal AH, Latif AN, Masalmani YA, Bertollini R, Raddad LJA. Outcomes Among Patients with Breakthrough SARS-CoV-2 Infection After Vaccination. Int J Infect Dis. 2021 Sep;110:353-358. doi: 10.1016/j.ijid.2021.08.008. Epub 2021 Aug 8. PMID: 34375762; PMCID: PMC8349447. |
| Butt, 2021 | Butt AA, Yan P, Shaikh OS, Mayr FB, Omer SB. Rate and Risk Factors for Severe/Critical Disease Among Fully Vaccinated Persons with Breakthrough SARS-CoV-2 Infection in a High-risk National Population. Clin Infect Dis. 2021 Dec 10:ciab1023. doi: 10.1093/cid/ciab1023. Epub ahead of print. PMID: 34893812. |
| Butt, 2021 | Butt AA, Omer SB, Yan P, Shaikh OS, Mayr FB. SARS-CoV-2 Vaccine Effectiveness in a High-Risk National Population in a Real-World Setting. Ann Intern Med. 2021 Oct;174(10):1404-1408. doi: 10.7326/M21-1577. Epub 2021 Jul 20. PMID: 34280332; PMCID: PMC8381771. |
| Butt, 2021 | Butt AA, Chemaitelly H, Al Khal A, Coyle PV, Saleh H, Kaleeckal AH, Latif AN, Bertollini R, Abou-Samra AB, Abu-Raddad LJ. SARS-CoV-2 vaccine effectiveness in preventing confirmed infection in pregnant women. J Clin Invest. 2021 Dec 1;131(23):e153662. doi: 10.1172/JCI153662. PMID: 34618693; PMCID: PMC8631593. |
| Butt, 2021 | Butt AA, Dargham SR, Chemaitelly H, Al Khal A, Tang P, Hasan MR, Coyle PV, Thomas AG, Borham AM, Concepcion EG, Kaleeckal AH, Latif AN, Bertollini R, Abou-Samra AB, Abu-Raddad LJ. Severity of Illness in Persons Infected With the SARS-CoV-2 Delta Variant vs Beta Variant in Qatar. JAMA Intern Med. 2021 Dec 22. doi: 10.1001/jamainternmed.2021.7949. Epub ahead of print. PMID: 34935861. |
| Butt, 2022 | Butt AA, Talisa VB, Yan P, Shaikh OS, Omer SB, Mayr FB. Real-world Effectiveness of the SARS-CoV-2 mRNA Vaccines in Preventing Confirmed Infection in Patients on Chronic Hemodialysis. Clin Infect Dis. 2022 Feb 9:ciac118. doi: 10.1093/cid/ciac118. Epub ahead of print. PMID: 35139175. |
| Cabezas, 2021 | Cabezas, Carmen and Coma, Ermengol and Mora-Fernandez, Nuria and Li, Xintong and Martinez-Marcos, Montse and Fina-Aviles, Francesc and Fabregas, Mireia and Hermosilla, Eduardo and Jover, Angel and Contel, Juan Carlos and Lejardi, Yolanda and Enfedaque, Belen and Argimon, Josep Maria and Medina-Peralta, Manuel and Prieto-Alhambra, Daniel, Effects of BNT162b2 mRNA Vaccination on COVID-19 Disease, Hospitalisation and Mortality in Nursing Homes and Healthcare Workers: A Prospective Cohort Study Including 28,594 Nursing Home Residents, 26,238 Nursing Home Staff, and 61,951 Healthcare Workers in Catalonia. Available at SSRN: https://ssrn.com/abstract=3815682 or http://dx.doi.org/10.2139/ssrn.3815682 |
| Callaghan, 2022 | Callaghan CJ, Mumford L, Curtis RMK, Williams SV, Whitaker H, Andrews N, Lopez Bernal J, Ushiro-Lumb I, Pettigrew GJ, Thorburn D, Forsythe JLR, Ravanan R; NHSBT Organ and Tissue Donation and Transplantation Clinical Team. Real-world Effectiveness of the Pfizer-BioNTech BNT162b2 and Oxford-AstraZeneca ChAdOx1-S Vaccines Against SARS-CoV-2 in Solid Organ and Islet Transplant Recipients. Transplantation. 2022 Jan 4. doi: 10.1097/TP.0000000000004059. Epub ahead of print. PMID: 34982758. |
| Carazo, 2021 | Carazo S, Talbot D, Boulianne N, Brisson M, Gilca R, Deceuninck G, Brousseau N, Drolet M, Ouakki M, Sauvageau C, Barkati S, Fortin É, Carignan A, De Wals P, Skowronski DM, De Serres G. Single-dose mRNA vaccine effectiveness against SARS-CoV-2 in healthcare workers extending 16 weeks post-vaccination: a test-negative design from Quebec, Canada. Clin Infect Dis. 2021 Aug 30:ciab739. doi: 10.1093/cid/ciab739. Epub ahead of print. PMID: 34460902; PMCID: PMC8522396. |
| Cavanaugh, 2021 | Cavanaugh AM, Fortier S, Lewis P, Arora V, Johnson M, George K, Tobias J, Lunn S, Miller T, Thoroughman D, Spicer KB. COVID-19 Outbreak Associated with a SARS-CoV-2 R.1 Lineage Variant in a Skilled Nursing Facility After Vaccination Program - Kentucky, March 2021. MMWR Morb Mortal Wkly Rep. 2021 Apr 30;70(17):639-643. doi: 10.15585/mmwr.mm7017e2. PMID: 33914720; PMCID: PMC8084128. |
| Cerqueira-Silva, 2021 | Cerqueira-Silva T, Oliveira VA, Boaventura VS, Pescarini JM, Júnior JB, Machado TM, Flores-Ortiz R, Penna GO, Ichihara MY, de Barros JV, Barreto ML, Werneck GL, Barral-Netto M. Influence of age on the effectiveness and duration of protection of Vaxzevria and CoronaVac vaccines: A population-based study. Lancet Reg Health Am. 2022 Feb;6:100154. doi: 10.1016/j.lana.2021.100154. Epub 2021 Dec 22. PMID: 34957437; PMCID: PMC8692070. |
| Cocchio, 2022 | Cocchio S, Zabeo F, Facchin G, Piva N, Furlan P, Nicoletti M, Saia M, Tonon M, Mongillo M, Russo F, Baldo V. The Effectiveness of a Diverse COVID-19 Vaccine Portfolio and Its Impact on the Persistence of Positivity and Length of Hospital Stays: The Veneto Region’s Experience. Vaccines (Basel). 2022 Jan 11;10(1):107. doi: 10.3390/vaccines10010107. PMID: 35062767; PMCID: PMC8777781. |
| Coggiola, 2021 | Coggiola M, Clemente G, Frammartino R, Grillo E, Corradi A, Corezzi M, Kakaa O, Paradisi E, Tuffanelli A, Alfonso Pensamiento MC, Godono A, Pira E. SARS-CoV-2 infection: efficacy of extensive vaccination of the healthcare workforce in a large Italian hospital. Med Lav. 2021 Dec 23;112(6):465-476. doi: 10.23749/mdl.v112i6.12124. PMID: 34939622. |
| Cohn, 2021 | Cohn BA, Cirillo PM, Murphy CC, Krigbaum NY, Wallace AW. SARS-CoV-2 vaccine protection and deaths among US veterans during 2021. Science. 2021 Nov 4:eabm0620. doi: 10.1126/science.abm0620. Epub ahead of print. PMID: 34735261. |
| Collie, 2021 | Collie S, Champion J, Moultrie H, Bekker LG, Gray G. Effectiveness of BNT162b2 Vaccine against Omicron Variant in South Africa. N Engl J Med. 2021 Dec 29. doi: 10.1056/NEJMc2119270. Epub ahead of print. PMID: 34965358. |
| Consonni, 2021 | Consonni, D.; Bono, P.; Oggioni, M.; Renteria, S. U.; Piatti, A.; Castaldi, S.; Muscatello, A.; Carugno, M.; Riboldi, L.; Ceriotti, F.; Bandera, A.; Gori, A.; Pesatori, A. C.; Lombardi, A. Effectiveness of COVID-19 vaccine in health care workers, Milan, Italy. Occupational and Environmental Medicine; 78(SUPPL 1):A84-A85, 2021. |
| Consonni, 2022 | Consonni D, Lombardi A, Mangioni D, et al. Effectiveness of BNT162b2 COVID-19 vaccine among healthcare workers of a large hospital, Milan, Italy Safety and Health at Work. 2022 Jan;13:S220-S220. PMCID: PMC8817444. |
| Corchado-Garcia, 2021 | Corchado-Garcia J, Zemmour D, Hughes T, Bandi H, Cristea-Platon T, Lenehan P, Pawlowski C, Bade S, O'Horo JC, Gores GJ, Williams AW, Badley AD, Halamka J, Virk A, Swift MD, Wagner T, Soundararajan V. Analysis of the Effectiveness of the Ad26.COV2.S Adenoviral Vector Vaccine for Preventing COVID-19. JAMA Netw Open. 2021 Nov 1;4(11):e2132540. doi: 10.1001/jamanetworkopen.2021.32540. PMID: 34726743; PMCID: PMC8564583. |
| Dagan, 2021 | Dagan N, Barda N, Kepten E, Miron O, Perchik S, Katz MA, Hernán MA, Lipsitch M, Reis B, Balicer RD. BNT162b2 mRNA Covid-19 Vaccine in a Nationwide Mass Vaccination Setting. N Engl J Med. 2021 Apr 15;384(15):1412-1423. doi: 10.1056/NEJMoa2101765. Epub 2021 Feb 24. PMID: 33626250; PMCID: PMC7944975. |
| Dagan, 2021 | Dagan N, Barda N, Biron-Shental T, Makov-Assif M, Key C, Kohane IS, Hernán MA, Lipsitch M, Hernandez-Diaz S, Reis BY, Balicer RD. Effectiveness of the BNT162b2 mRNA COVID-19 vaccine in pregnancy. Nat Med. 2021 Oct;27(10):1693-1695. doi: 10.1038/s41591-021-01490-8. Epub 2021 Sep 7. PMID: 34493859. |
| de Gier, 2021 | Brechje de Gier, Marjolein Kooijman, Jeanet Kemmeren, Nicolette de Keizer, Dave Dongelmans, Senna C.J.L. van Iersel, Jan van de Kassteele, Stijn P. Andeweg, the RIVM COVID-19 epidemiology and surveillance team, Hester E. de Melker, Susan J. M. Hahné, Mirjam J. Knol, Susan van den Hof. COVID-19 vaccine effectiveness against hospitalizations and ICU admissions in the Netherlands, April- August 2021. medRxiv 2021.09.15.21263613; doi: https://doi.org/10.1101/2021.09.15.21263613 |
| Del Cura-Bilbao, 2022 | Del Cura-Bilbao A, López-Mendoza H, Chaure-Pardos A, Vergara-Ugarriza A, Guimbao-Bescós J. Effectiveness of 3 COVID-19 Vaccines in Preventing SARS-CoV-2 Infections, January-May 2021, Aragon, Spain. Emerg Infect Dis. 2022 Mar;28(3):591-598. doi: 10.3201/eid2803.212027. PMID: 35195514. |
| Desai, 2021 | Desai A, Desai P, Mehta J, Sachora W, Bharti N, Patel T, Sukhwani K, Jain A, Sorathiya D, Nanda V, Mehta P, Desai A. Measuring the impact of a single dose of ChAdOx1 nCoV-19 (recombinant) coronavirus vaccine on hospital stay, ICU requirement, and mortality outcome in a tertiary care centre. Int J Infect Dis. 2021 Dec;113:282-287. doi: 10.1016/j.ijid.2021.10.032. Epub 2021 Oct 21. PMID: 34688949; PMCID: PMC8529545. |
| Domi, 2021 | Domi M, Leitson M, Gifford D, Nicolaou A, Sreenivas K, Bishnoi C. The BNT162b2 vaccine is associated with lower new COVID-19 cases in nursing home residents and staff. J Am Geriatr Soc. 2021 Aug;69(8):2079-2089. doi: 10.1111/jgs.17224. Epub 2021 May 18. PMID: 33955567; PMCID: PMC8242377. |
| Dorabawila, 2022 | Vajeera Dorabawila, Dina Hoefer, Ursula E. Bauer, Mary T. Bassett, Emily Lutterloh, Eli S. Rosenberg. Effectiveness of the BNT162b2 vaccine among children 5-11 and 12-17 years in New York after the Emergence of the Omicron Variant. medRxiv 2022.02.25.22271454; doi: https://doi.org/10.1101/2022.02.25.22271454 |
| Drawz, 2022 | Drawz PE, DeSilva M, Bodurtha P, Benitez GV, Murray A, Chamberlain AM, Dudley RA, Waring S, Kharbanda AB, Murphy D, Muscoplat MH, Melendez V, Margolis KL, McFarling L, Lupu R, Winkelman TNA, Johnson SG. Effectiveness of BNT162b2 and mRNA-1273 Second Doses and Boosters for SARS-CoV-2 infection and SARS-CoV-2 Related Hospitalizations: A Statewide Report from the Minnesota Electronic Health Record Consortium. Clin Infect Dis. 2022 Feb 7:ciac110. doi: 10.1093/cid/ciac110. Epub ahead of print. PMID: 35137021. |
| Embi, 2021 | Embi PJ, Levy ME, Naleway AL, Patel P, Gaglani M, Natarajan K, Dascomb K, Ong TC, Klein NP, Liao IC, Grannis SJ, Han J, Stenehjem E, Dunne MM, Lewis N, Irving SA, Rao S, McEvoy C, Bozio CH, Murthy K, Dixon BE, Grisel N, Yang DH, Goddard K, Kharbanda AB, Reynolds S, Raiyani C, Fadel WF, Arndorfer J, Rowley EA, Fireman B, Ferdinands J, Valvi NR, Ball SW, Zerbo O, Griggs EP, Mitchell PK, Porter RM, Kiduko SA, Blanton L, Zhuang Y, Steffens A, Reese SE, Olson N, Williams J, Dickerson M, McMorrow M, Schrag SJ, Verani JR, Fry AM, Azziz-Baumgartner E, Barron MA, Thompson MG, DeSilva MB. Effectiveness of 2-Dose Vaccination with mRNA COVID-19 Vaccines Against COVID-19-Associated Hospitalizations Among Immunocompromised Adults - Nine States, January-September 2021. MMWR Morb Mortal Wkly Rep. 2021 Nov 5;70(44):1553-1559. doi: 10.15585/mmwr.mm7044e3. PMID: 34735426; PMCID: PMC8568092. |
| Emborg, 2021 | Hanne-Dorthe Emborg, Palle Valentiner-Branth, Astrid Blicher Schelde, Katrine Finderup Nielsen, Mie Agermose Gram, Ida Rask Moustsen-Helms, Manon Chaine, Ulla Holten Seidelin, Jens Nielsen. Vaccine effectiveness of the BNT162b2 mRNA COVID-19 vaccine against RT-PCR confirmed SARS-CoV-2 infections, hospitalisations and mortality in prioritised risk groups. medRxiv 2021.05.27.21257583; doi: https://doi.org/10.1101/2021.05.27.21257583 |
| Fabiani, 2021 | Fabiani M, Ramigni M, Gobbetto V, Mateo-Urdiales A, Pezzotti P, Piovesan C. Effectiveness of the Comirnaty (BNT162b2, BioNTech/Pfizer) vaccine in preventing SARS-CoV-2 infection among healthcare workers, Treviso province, Veneto region, Italy, 27 December 2020 to 24 March 2021. Euro Surveill. 2021 Apr;26(17):2100420. doi: 10.2807/1560-7917.ES.2021.26.17.2100420. PMID: 33928898; PMCID: PMC8086247. |
| Fabiani, 2022 | Fabiani M, Puopolo M, Morciano C, Spuri M, Spila Alegiani S, Filia A, D'Ancona F, Del Manso M, Riccardo F, Tallon M, Proietti V, Sacco C, Massari M, Da Cas R, Mateo-Urdiales A, Siddu A, Battilomo S, Bella A, Palamara AT, Popoli P, Brusaferro S, Rezza G, Menniti Ippolito F, Pezzotti P; Italian Integrated Surveillance of covid-19 study group and Italian covid-19 Vaccines Registry group. Effectiveness of mRNA vaccines and waning of protection against SARS-CoV-2 infection and severe covid-19 during predominant circulation of the delta variant in Italy: retrospective cohort study. BMJ. 2022 Feb 10;376:e069052. doi: 10.1136/bmj-2021-069052. PMID: 35144968. |
| Farah, 2022 | Zeina Farah, Nadine Haddad, Hala Abou El Naja, Majd Saleh, Pamela Mrad, Nada Ghosn. Effectiveness of Pfizer-BioNTech Vaccine Against COVID-19 Associated Hospitalizations among Lebanese Adults ≥75 years - Lebanon, April-May 2021. medRxiv 2022.01.19.22269514; doi: https://doi.org/10.1101/2022.01.19.22269514 |
| Fisman, 2022 | David N. Fisman, Nelson Lee, Ashleigh R. Tuite. Timing of Breakthrough Infection Risk After Vaccination Against SARS-CoV-2. medRxiv 2022.01.04.22268773; doi: https://doi.org/10.1101/2022.01.04.22268773 |
| Flacco, 2021 | Flacco ME, Soldato G, Acuti Martellucci C, Carota R, Di Luzio R, Caponetti A, Manzoli L. Interim Estimates of COVID-19 Vaccine Effectiveness in a Mass Vaccination Setting: Data from an Italian Province. Vaccines (Basel). 2021 Jun 10;9(6):628. doi: 10.3390/vaccines9060628. PMID: 34200538; PMCID: PMC8227269. |
| Florea, 2021 | Ana Florea, Lina S. Sy, Yi Luo, Lei Qian, Katia J. Bruxvoort, Bradley K. Ackerson, Gina S. Lee, Jennifer H. Ku, Julia E. Tubert, Yun Tian, Carla A. Talarico, Hung Fu Tseng. Durability of mRNA-1273 against COVID-19 in the time of Delta: Interim results from an observational cohort study. medRxiv 2021.12.13.21267620; doi: https://doi.org/10.1101/2021.12.13.21267620 |
| Fournier, 2022 | Fournier PE, Houhamdi L, Colson P, Cortaredona S, Delorme L, Cassagne C, Lagier JC, Chaudet H, Tissot-Dupont H, Giraud-Gatineau A, Fenollar F, Million M, Raoult D. SARS-CoV-2 Vaccination and Protection Against Clinical Disease: A Retrospective Study, Bouches-du-Rhône District, Southern France, 2021. Front Microbiol. 2022 Jan 18;12:796807. doi: 10.3389/fmicb.2021.796807. PMID: 35116013; PMCID: PMC8803903. |
| Fowlkes, 2021 | Fowlkes A, Gaglani M, Groover K, Thiese MS, Tyner H, Ellingson K; HEROES-RECOVER Cohorts. Effectiveness of COVID-19 Vaccines in Preventing SARS-CoV-2 Infection Among Frontline Workers Before and During B.1.617.2 (Delta) Variant Predominance - Eight U.S. Locations, December 2020-August 2021. MMWR Morb Mortal Wkly Rep. 2021 Aug 27;70(34):1167-1169. doi: 10.15585/mmwr.mm7034e4. PMID: 34437521; PMCID: PMC8389394. |
| Gaio, 2022 | Vânia Gaio, Adriana Silva, Palmira Amaral, João Faro Viana, Pedro Pinto Leite, Carlos Matias Dias, Irina Kislaya, Baltazar Nunes, Ausenda Machado. COVID-19 vaccine effectiveness among healthcare workers in Portugal: results from a hospital-based cohort study, December 2020 to November 2021. medRxiv 2022.01.07.22268889; doi: https://doi.org/10.1101/2022.01.07.22268889 |
| Garvey, 2021 | Garvey MI, Wilkinson MAC, Holden E, Shields A, Robertson A, Richter A, Ball S. Early observations on the impact of a healthcare worker COVID-19 vaccination programme at a major UK tertiary centre. J Infect. 2021 Jul;83(1):119-145. doi: 10.1016/j.jinf.2021.04.027. Epub 2021 Apr 29. PMID: 33933530; PMCID: PMC8081749. |
| Ge, 2022 | Jin Ge, Jean C. Digitale, Mark J. Pletcher, Jennifer C. Lai, the N3C Consortium. Breakthrough SARS-CoV-2 Infection Outcomes in Vaccinated Patients with Chronic Liver Disease and Cirrhosis: A National COVID Cohort Collaborative Study. medRxiv 2022.02.25.22271490; doi: https://doi.org/10.1101/2022.02.25.22271490 |
| Ghosh, 2021 | Ghosh S, Shankar S, Chatterjee K, Chatterjee K, Yadav AK, Pandya K, Suryam V, Agrawal S, Ray S, Phutane V, Datta R. COVISHIELD (AZD1222) VaccINe effectiveness among healthcare and frontline Workers of INdian Armed Forces: Interim results of VIN-WIN cohort study. Med J Armed Forces India. 2021 Jul;77(Suppl 2):S264-S270. doi: 10.1016/j.mjafi.2021.06.032. Epub 2021 Jul 26. PMID: 34334892; PMCID: PMC8313084. |
| Giansante, 2021 | Giansante C, Stivanello E, Perlangeli V, Ferretti F, Marzaroli P, Musti MA, Pizzi L, Resi D, Saraceni S, Pandolfi P. COVID-19 vaccine effectiveness among the staff of the Bologna Health Trust, Italy, December 2020-April 2021. Acta Biomed. 2021 Sep 2;92(4):e2021270. doi: 10.23750/abm.v92i4.11896. PMID: 34487060; PMCID: PMC8477111. |
| Glampson, 2021 | Glampson B, Brittain J, Kaura A, Mulla A, Mercuri L, Brett SJ, Aylin P, Sandall T, Goodman I, Redhead J, Saravanakumar K, Mayer EK. Assessing COVID-19 Vaccine Uptake and Effectiveness Through the North West London Vaccination Program: Retrospective Cohort Study. JMIR Public Health Surveill. 2021 Sep 17;7(9):e30010. doi: 10.2196/30010. PMID: 34265740; PMCID: PMC8451961. |
| Glatman-Freedman, 2021 | Glatman-Freedman A, Bromberg M, Dichtiar R, Hershkovitz Y, Keinan-Boker L. The BNT162b2 vaccine effectiveness against new COVID-19 cases and complications of breakthrough cases: A nation-wide retrospective longitudinal multiple cohort analysis using individualised data. EBioMedicine. 2021 Oct;72:103574. doi: 10.1016/j.ebiom.2021.103574. Epub 2021 Sep 17. PMID: 34537449; PMCID: PMC8445746. |
| Glatman-Freedman, 2021 | Glatman-Freedman A, Hershkovitz Y, Kaufman Z, Dichtiar R, Keinan-Boker L, Bromberg M. Effectiveness of BNT162b2 Vaccine in Adolescents during Outbreak of SARS-CoV-2 Delta Variant Infection, Israel, 2021. Emerg Infect Dis. 2021 Nov;27(11):2919-2922. doi: 10.3201/eid2711.211886. Epub 2021 Sep 27. PMID: 34570694; PMCID: PMC8544958. |
| Goldberg, 2021 | Yair Goldberg, Micha Mandel, Yonatan Woodbridge, Ronen Fluss, Ilya Novikov, Rami Yaari, Arnona Ziv, Laurence Freedman, Amit Huppert. Protection of previous SARS-CoV-2 infection is similar to that of BNT162b2 vaccine protection: A three-month nationwide experience from Israel. medRxiv 2021.04.20.21255670; doi: https://doi.org/10.1101/2021.04.20.21255670 |
| Goldin, 2022 | Goldin S, Adler L, Azuri J, Mendel L, Haviv S, Maimon N. BNT162b2 mRNA COVID-19 (Comirnaty) Vaccine Effectiveness in Elderly Patients Who Live in Long-Term Care Facilities: A Nationwide Cohort. Gerontology. 2022 Feb 8:1-8. doi: 10.1159/000521899. Epub ahead of print. PMID: 35134810. |
| Goldshtein, 2021 | Goldshtein I, Nevo D, Steinberg DM, Rotem RS, Gorfine M, Chodick G, Segal Y. Association Between BNT162b2 Vaccination and Incidence of SARS-CoV-2 Infection in Pregnant Women. JAMA. 2021 Aug 24;326(8):728-735. doi: 10.1001/jama.2021.11035. PMID: 34251417; PMCID: PMC8276131. |
| Gomes, 2021 | Gomes D, Beyerlein A, Katz K, Hoelscher G, Nennstiel U, Liebl B, Überla K, von Kries R. Is the BNT162b2 COVID-19 vaccine effective in elderly populations? Results from population data from Bavaria, Germany. PLoS One. 2021 Nov 5;16(11):e0259370. doi: 10.1371/journal.pone.0259370. PMID: 34739520; PMCID: PMC8570490. |
| González, 2021 | González S, Olszevicki S, Salazar M, Calabria A, Regairaz L, Marín L, Campos P, Varela T, Martínez VVG, Ceriani L, Garcia E, Kreplak N, Pifano M, Estenssoro E, Marsico F. Effectiveness of the first component of Gam-COVID-Vac (Sputnik V) on reduction of SARS-CoV-2 confirmed infections, hospitalisations and mortality in patients aged 60-79: a retrospective cohort study in Argentina. EClinicalMedicine. 2021 Oct;40:101126. doi: 10.1016/j.eclinm.2021.101126. Epub 2021 Sep 12. PMID: 34541480; PMCID: PMC8435263. |
| Gram, 2021 | Gram MA, Nielsen J, Schelde AB, Nielsen KF, Moustsen-Helms IR, Sørensen AKB, Valentiner-Branth P, Emborg HD. Vaccine effectiveness against SARS-CoV-2 infection, hospitalization, and death when combining a first dose ChAdOx1 vaccine with a subsequent mRNA vaccine in Denmark: A nationwide population-based cohort study. PLoS Med. 2021 Dec 17;18(12):e1003874. doi: 10.1371/journal.pmed.1003874. Epub ahead of print. PMID: 34919548. |
| Grannis, 2021 | Grannis SJ, Rowley EA, Ong TC, Stenehjem E, Klein NP, DeSilva MB, Naleway AL, Natarajan K, Thompson MG; VISION Network. Interim Estimates of COVID-19 Vaccine Effectiveness Against COVID-19-Associated Emergency Department or Urgent Care Clinic Encounters and Hospitalizations Among Adults During SARS-CoV-2 B.1.617.2 (Delta) Variant Predominance - Nine States, June-August 2021. MMWR Morb Mortal Wkly Rep. 2021 Sep 17;70(37):1291-1293. doi: 10.15585/mmwr.mm7037e2. PMID: 34529642; PMCID: PMC8445373. |
| Grant, 2021 | Grant R, Charmet T, Schaeffer L, Galmiche S, Madec Y, Von Platen C, Chény O, Omar F, David C, Rogoff A, Paireau J, Cauchemez S, Carrat F, Septfons A, Levy-Bruhl D, Mailles A, Fontanet A. Impact of SARS-CoV-2 Delta variant on incubation, transmission settings and vaccine effectiveness: Results from a nationwide case-control study in France. Lancet Reg Health Eur. 2021 Nov 26:100278. doi: 10.1016/j.lanepe.2021.100278. Epub ahead of print. PMID: 34849500; PMCID: PMC8616730. |
| Gras-Valentí, 2021 | Gras-Valentí P, Chico-Sánchez P, Algado-Sellés N, Jiménez-Sepúlveda NJ, Gómez-Sotero IL, Fuster-Pérez M, Cartagena-Llopis L, Sánchez-Valero M, Cerezo-Milán P, Martínez-Tornero I, Tremiño-Sánchez L, Nadal-Morante V, Monerris-Palmer M, Esclapez-Martínez A, Morenode Arcos-Fuentes E, Escalada-Martín I, Escribano-Cañadas I, Merino-Lucas E, Rodríguez-Díaz JC, Sánchez-Payá J. Efectividad de la primera dosis de vacuna BNT162b2 para prevenir la COVID-19 en personal sanitario [Effectiveness of the first dose of BNT162b2 vaccine to preventing covid-19 in healthcare personnel.]. Rev Esp Salud Publica. 2021 Apr 29;95:e202104070. Spanish. PMID: 33913444. |
| Gray, 2021 | Glenda E Gray, Shirley Collie, Nigel Garrett, Ameena Goga, Jared Champion, Matthew Zylstra, Tarylee Reddy, Nonhlanhla Yende, Ishen Seocharan, Azwi Takalani, Ian Sanne, Fatima Mayat, Jacky Odhiambo, Lesley Bamford, Harry Moultrie, Lara Fairall, Linda-Gail Bekker. Vaccine effectiveness against hospital admission in South African health care workers who received a homologous booster of Ad26.COV2 during an Omicron COVID19 wave: Preliminary Results of the Sisonke 2 Study. medRxiv 2021.12.28.21268436; doi: https://doi.org/10.1101/2021.12.28.21268436 |
| Grgič, 2022 | Grgič Vitek M, Klavs I, Učakar V, Serdt M, Mrzel M, Vrh M, Fafangel M. Vaccine effectiveness against severe acute respiratory infections (SARI) COVID-19 hospitalisations estimated from real-world surveillance data, Slovenia, October 2021. Euro Surveill. 2022 Jan;27(1). doi: 10.2807/1560-7917.ES.2022.27.1.2101110. PMID: 34991780. |
| Guijarro, 2021 | Guijarro C, Galán I, Martínez-Ponce D, Pérez-Fernández E, Goyanes MJ, Castilla V, Velasco M. SARS-CoV-2 new infections among health-care workers after the first dose of the BNT162b2 mRNA COVID-19 vaccine. A hospital-wide cohort study. Clin Microbiol Infect. 2021 Nov;27(11):1699.e1-1699.e4. doi: 10.1016/j.cmi.2021.06.026. Epub 2021 Jun 29. PMID: 34197936; PMCID: PMC8239210. |
| Gupta, 2021 | Gupta K, O'Brien WJ, Bellino P, Linsenmeyer K, Doshi SJ, Sprague RS, Charness ME. Incidence of SARS-CoV-2 Infection in Health Care Workers After a Single Dose of mRNA-1273 Vaccine. JAMA Netw Open. 2021 Jun 1;4(6):e2116416. doi: 10.1001/jamanetworkopen.2021.16416. PMID: 34132795; PMCID: PMC8209555. |
| Haas, 2021 | Haas EJ, Angulo FJ, McLaughlin JM, Anis E, Singer SR, Khan F, Brooks N, Smaja M, Mircus G, Pan K, Southern J, Swerdlow DL, Jodar L, Levy Y, Alroy-Preis S. Impact and effectiveness of mRNA BNT162b2 vaccine against SARS-CoV-2 infections and COVID-19 cases, hospitalisations, and deaths following a nationwide vaccination campaign in Israel: an observational study using national surveillance data. Lancet. 2021 May 15;397(10287):1819-1829. doi: 10.1016/S0140-6736(21)00947-8. Epub 2021 May 5. Erratum in: Lancet. 2021 Jul 17;398(10296):212. PMID: 33964222; PMCID: PMC8099315. |
| Hall, 2021 | Hall VJ, Foulkes S, Saei A, Andrews N, Oguti B, Charlett A, Wellington E, Stowe J, Gillson N, Atti A, Islam J, Karagiannis I, Munro K, Khawam J, Chand MA, Brown CS, Ramsay M, Lopez-Bernal J, Hopkins S; SIREN Study Group. COVID-19 vaccine coverage in health-care workers in England and effectiveness of BNT162b2 mRNA vaccine against infection (SIREN): a prospective, multicentre, cohort study. Lancet. 2021 May 8;397(10286):1725-1735. doi: 10.1016/S0140-6736(21)00790-X. Epub 2021 Apr 23. PMID: 33901423; PMCID: PMC8064668. |
| Hall, 2022 | Hall V, Foulkes S, Insalata F, Kirwan P, Saei A, Atti A, Wellington E, Khawam J, Munro K, Cole M, Tranquillini C, Taylor-Kerr A, Hettiarachchi N, Calbraith D, Sajedi N, Milligan I, Themistocleous Y, Corrigan D, Cromey L, Price L, Stewart S, de Lacy E, Norman C, Linley E, Otter AD, Semper A, Hewson J, D'Arcangelo S, Chand M, Brown CS, Brooks T, Islam J, Charlett A, Hopkins S; SIREN Study Group. Protection against SARS-CoV-2 after Covid-19 Vaccination and Previous Infection. N Engl J Med. 2022 Feb 16. doi: 10.1056/NEJMoa2118691. Epub ahead of print. PMID: 35172051. |
| Hansen, 2021 | Christian Holm Hansen, Astrid Blicher Schelde, Ida Rask Moustsen-Helm, Hanne-Dorthe Emborg, Tyra Grove Krause, Kåre Mølbak, Palle Valentiner-Branth.Vaccine effectiveness against SARS-CoV-2 infection with the Omicron or Delta variants following a two-dose or booster BNT162b2 or mRNA-1273 vaccination series: A Danish cohort study. medRxiv 2021.12.20.21267966; doi: https://doi.org/10.1101/2021.12.20.21267966 |
| Heftdal, 2022 | Heftdal LD, Schultz M, Lange T, Knudsen AD, Fogh K, Hasselbalch RB, Linander CB, Kallemose T, Bundgaard H, Grønbæk K, Valentiner-Branth P, Iversen K, Nielsen SD. Incidence of positive SARS-CoV-2 PCR after COVID-19 vaccination with up to eight months of follow-up: Real life data from the Capital Region of Denmark. Clin Infect Dis. 2022 Jan 7:ciac012. doi: 10.1093/cid/ciac012. Epub ahead of print. PMID: 35015858. |
| Hitchings, 2021 | Hitchings MDT, Ranzani OT, Dorion M, D'Agostini TL, de Paula RC, de Paula OFP, de Moura Villela EF, Torres MSS, de Oliveira SB, Schulz W, Almiron M, Said R, de Oliveira RD, Silva PV, de Araújo WN, Gorinchteyn JC, Andrews JR, Cummings DAT, Ko AI, Croda J. Effectiveness of ChAdOx1 vaccine in older adults during SARS-CoV-2 Gamma variant circulation in São Paulo. Nat Commun. 2021 Oct 28;12(1):6220. doi: 10.1038/s41467-021-26459-6. PMID: 34711813; PMCID: PMC8553924. |
| Hyams, 2021 | Hyams C, Marlow R, Maseko Z, King J, Ward L, Fox K, Heath R, Tuner A, Friedrich Z, Morrison L, Ruffino G, Antico R, Adegbite D, Szasz-Benczur Z, Garcia Gonzalez M, Oliver J, Danon L, Finn A. Effectiveness of BNT162b2 and ChAdOx1 nCoV-19 COVID-19 vaccination at preventing hospitalisations in people aged at least 80 years: a test-negative, case-control study. Lancet Infect Dis. 2021 Nov;21(11):1539-1548. doi: 10.1016/S1473-3099(21)00330-3. Epub 2021 Jun 23. Erratum in: Lancet Infect Dis. 2021 Aug;21(8):e208. PMID: 34174190; PMCID: PMC8221734. |
| Chadeau-Hyam, 2021 | Marc Chadeau-Hyam, Oliver Eales, Barbara Bodinier, Haowei Wang, David Haw, Matthew Whitaker, Caroline E. Walters, Jakob Jonnerby, Christina Atchison, Peter J. Diggle, Andrew J. Page, Deborah Ashby, Wendy Barclay, Graham Taylor, Graham Cooke, Helen Ward, Ara Darzi, Christl A. Donnelly, Paul Elliott. REACT-1 round 15 final report: Increased breakthrough SARS-CoV-2 infections among adults who had received two doses of vaccine, but booster doses and first doses in children are providing important protection. medRxiv 2021.12.14.21267806; doi: https://doi.org/10.1101/2021.12.14.21267806 |
| Chadeau-Hyam, 2022 | Chadeau-Hyam M, Wang H, Eales O, Haw D, Bodinier B, Whitaker M, Walters CE, Ainslie KEC, Atchison C, Fronterre C, Diggle PJ, Page AJ, Trotter AJ, Ashby D, Barclay W, Taylor G, Cooke G, Ward H, Darzi A, Riley S, Donnelly CA, Elliott P; COVID-19 Genomics UK consortium. SARS-CoV-2 infection and vaccine effectiveness in England (REACT-1): a series of cross-sectional random community surveys. Lancet Respir Med. 2022 Jan 24:S2213-2600(21)00542-7. doi: 10.1016/S2213-2600(21)00542-7. Epub ahead of print. PMID: 35085490; PMCID: PMC8786320. |
| Charmet, 2021 | Charmet T, Schaeffer L, Grant R, Galmiche S, Chény O, Von Platen C, Maurizot A, Rogoff A, Omar F, David C, Septfons A, Cauchemez S, Gaymard A, Lina B, Lefrancois LH, Enouf V, van der Werf S, Mailles A, Levy-Bruhl D, Carrat F, Fontanet A. Impact of original, B.1.1.7, and B.1.351/P.1 SARS-CoV-2 lineages on vaccine effectiveness of two doses of COVID-19 mRNA vaccines: Results from a nationwide case-control study in France. Lancet Reg Health Eur. 2021 Sep;8:100171. doi: 10.1016/j.lanepe.2021.100171. Epub 2021 Jul 13. PMID: 34278372; PMCID: PMC8277121. |
| Chemaitelly, 2021 | Chemaitelly H, Tang P, Hasan MR, AlMukdad S, Yassine HM, Benslimane FM, Al Khatib HA, Coyle P, Ayoub HH, Al Kanaani Z, Al Kuwari E, Jeremijenko A, Kaleeckal AH, Latif AN, Shaik RM, Abdul Rahim HF, Nasrallah GK, Al Kuwari MG, Al Romaihi HE, Butt AA, Al-Thani MH, Al Khal A, Bertollini R, Abu-Raddad LJ. Waning of BNT162b2 Vaccine Protection against SARS-CoV-2 Infection in Qatar. N Engl J Med. 2021 Dec 9;385(24):e83. doi: 10.1056/NEJMoa2114114. Epub 2021 Oct 6. PMID: 34614327; PMCID: PMC8522799. |
| Chemaitelly, 2021 | Chemaitelly H, Yassine HM, Benslimane FM, Al Khatib HA, Tang P, Hasan MR, Malek JA, Coyle P, Ayoub HH, Al Kanaani Z, Al Kuwari E, Jeremijenko A, Kaleeckal AH, Latif AN, Shaik RM, Abdul Rahim HF, Nasrallah GK, Al Kuwari MG, Al Romaihi HE, Al-Thani MH, Al Khal A, Butt AA, Bertollini R, Abu-Raddad LJ. mRNA-1273 COVID-19 vaccine effectiveness against the B.1.1.7 and B.1.351 variants and severe COVID-19 disease in Qatar. Nat Med. 2021 Sep;27(9):1614-1621. doi: 10.1038/s41591-021-01446-y. Epub 2021 Jul 9. PMID: 34244681. |
| Chemaitelly, 2021 | Hiam Chemaitelly, Sawsan AlMukdad, Jobin Paravila Joy, Houssein H. Ayoub, Hadi M. Yassine, Fatiha M. Benslimane, Hebah A. Al Khatib, Patrick Tang, Mohammad R. Hasan, Peter Coyle, Zaina Al Kanaani, Einas Al Kuwari, Andrew Jeremijenko, Anvar Hassan Kaleeckal, Ali Nizar Latif, Riyazuddin Mohammad Shaik, Hanan F. Abdul Rahim, Gheyath K. Nasrallah, Mohamed Ghaith Al Kuwari, Adeel A. Butt, Hamad Eid Al Romaihi, Mohamed H. Al-Thani, Mohamad M. Alkadi, Omar Ali, Muna Al-Maslamani, Roberto Bertollini, Hassan Al Malki, Yousuf Almaslamani, Laith J. Abu-Raddad, Abdullatif Al Khal. SARS-CoV-2 vaccine effectiveness in immunosuppressed kidney transplant recipients. medRxiv 2021.08.07.21261578; doi: https://doi.org/10.1101/2021.08.07.21261578 |
| Chemaitelly, 2022 | Hiam Chemaitelly, Houssein H. Ayoub, Sawsan AlMukdad, Patrick Tang, Mohammad R. Hasan, Hadi M. Yassine, Hebah A. Al Khatib, Maria K. Smatti, Peter Coyle, Zaina Al Kanaani, Einas Al Kuwari, Andrew Jeremijenko, Anvar Hassan Kaleeckal, Ali Nizar Latif, Riyazuddin Mohammad Shaik, Hanan F. Abdul Rahim, Gheyath K. Nasrallah, Mohamed Ghaith Al Kuwari, Adeel A. Butt, Hamad Eid Al Romaihi, Mohamed H. Al-Thani, Abdullatif Al Khal, Roberto Bertollini, Laith J. Abu-Raddad. Duration of protection of BNT162b2 and mRNA-1273 COVID-19 vaccines against symptomatic SARS-CoV-2 Omicron infection in Qatar. medRxiv 2022.02.07.22270568; doi: https://doi.org/10.1101/2022.02.07.22270568 |
| Chia, 2021 | Chia PY, Xiang Ong SW, Chiew CJ, Ang LW, Chavatte JM, Mak TM, Cui L, Kalimuddin S, Chia WN, Tan CW, Ann Chai LY, Tan SY, Zheng S, Pin Lin RT, Wang L, Leo YS, Lee VJ, Lye DC, Young BE. Virological and serological kinetics of SARS-CoV-2 Delta variant vaccine-breakthrough infections: a multi-center cohort study. Clin Microbiol Infect. 2021 Nov 23:S1198-743X(21)00638-8. doi: 10.1016/j.cmi.2021.11.010. Epub ahead of print. PMID: 34826623; PMCID: PMC8608661. |
| Chico-Sánchez, 2021 | Chico-Sánchez P, Gras-Valentí P, Algado-Sellés N, Merino-Lucas E, Rodríguez-Díaz JC, Ronda-Pérez E, Sánchez-Payá J; Grupo COVID-19 de Medicina Preventiva. Efectividad de la vacuna BNT162b2 para prevenir la COVID-19 en personal sanitario [Effectiveness of BNT162b2 vaccine to preventing COVID-19 in healthcare personnel]. Gac Sanit. 2021 Nov 26:S0213-9111(21)00317-4. Spanish. doi: 10.1016/j.gaceta.2021.11.003. Epub ahead of print. PMID: 34952732; PMCID: PMC8616741. |
| Chin, 2021 | Chin ET, Leidner D, Zhang Y, Long E, Prince L, Li Y, Andrews JR, Studdert DM, Goldhaber-Fiebert JD, Salomon JA. Effectiveness of the mRNA-1273 Vaccine during a SARS-CoV-2 Delta Outbreak in a Prison. N Engl J Med. 2021 Dec 9;385(24):2300-2301. doi: 10.1056/NEJMc2114089. Epub 2021 Oct 20. PMID: 34670040; PMCID: PMC8552536. |
| Chin, 2022 | Chin ET, Leidner D, Zhang Y, Long E, Prince L, Schrag SJ, Verani JR, Wiegand RE, Alarid-Escudero F, Goldhaber-Fiebert JD, Studdert DM, Andrews JR, Salomon JA. Effectiveness of COVID-19 vaccines among incarcerated people in California state prisons: retrospective cohort study. Clin Infect Dis. 2022 Jan 27:ciab1032. doi: 10.1093/cid/ciab1032. Epub ahead of print. PMID: 35083482. |
| Chodick, 2021 | Chodick G, Tene L, Patalon T, Gazit S, Ben Tov A, Cohen D, Muhsen K. Assessment of Effectiveness of 1 Dose of BNT162b2 Vaccine for SARS-CoV-2 Infection 13 to 24 Days After Immunization. JAMA Netw Open. 2021 Jun 1;4(6):e2115985. doi: 10.1001/jamanetworkopen.2021.15985. PMID: 34097044; PMCID: PMC8185600. |
| Chodick, 2021 | Chodick G, Tene L, Rotem RS, Patalon T, Gazit S, Ben-Tov A, Weil C, Goldshtein I, Twig G, Cohen D, Muhsen K. The effectiveness of the TWO-DOSE BNT162b2 vaccine: analysis of real-world data. Clin Infect Dis. 2021 May 17:ciab438. doi: 10.1093/cid/ciab438. Epub ahead of print. PMID: 33999127; PMCID: PMC8240867. |
| Chung, 2021 | Chung H, He S, Nasreen S, Sundaram ME, Buchan SA, Wilson SE, Chen B, Calzavara A, Fell DB, Austin PC, Wilson K, Schwartz KL, Brown KA, Gubbay JB, Basta NE, Mahmud SM, Righolt CH, Svenson LW, MacDonald SE, Janjua NZ, Tadrous M, Kwong JC; Canadian Immunization Research Network (CIRN) Provincial Collaborative Network (PCN) Investigators. Effectiveness of BNT162b2 and mRNA-1273 covid-19 vaccines against symptomatic SARS-CoV-2 infection and severe covid-19 outcomes in Ontario, Canada: test negative design study. BMJ. 2021 Aug 20;374:n1943. doi: 10.1136/bmj.n1943. PMID: 34417165; PMCID: PMC8377789. |
| Chung, 2022 | Chung JR, Kim SS, Belongia EA, McLean HQ, King JP, Nowalk MP, Zimmerman RK, Moehling Geffel K, Martin ET, Monto AS, Lamerato LE, Gaglani M, Hoffman E, Volz M, Jackson ML, Jackson LA, Patel MM, Flannery B. Vaccine effectiveness against COVID-19 among symptomatic persons aged ≥12 years with reported contact with COVID-19 cases, February-September 2021. Influenza Other Respir Viruses. 2022 Feb 15. doi: 10.1111/irv.12973. Epub ahead of print. PMID: 35170231. |
| Iliaki , 2021 | Iliaki E, Lan FY, Christophi CA, Guidotti G, Jobrack AD, Buley J, Osgood R, Bruno-Murtha LA, Kales SN. COVID-19 Vaccine Effectiveness in a Diverse Urban Health Care Worker Population. Mayo Clin Proc. 2021 Dec;96(12):3180-3182. doi: 10.1016/j.mayocp.2021.10.005. Epub 2021 Oct 19. PMID: 34863402; PMCID: PMC8523483. |
| Ioannou, 2021 | Ioannou GN, Locke ER, O'Hare AM, Bohnert ASB, Boyko EJ, Hynes DM, Berry K. COVID-19 Vaccination Effectiveness Against Infection or Death in a National U.S. Health Care System: A Target Trial Emulation Study. Ann Intern Med. 2021 Dec 21:M21-3256. doi: 10.7326/M21-3256. Epub ahead of print. PMID: 34928700; PMCID: PMC8697485. |
| Iskander, 2021 | John Iskander, Jamie Frost, Sharon Russell, Jaspal Ahluwalia, Emily Ward, Shane Steiner, Dana Thomas, Paul Michaud. Effectiveness of Vaccination against Reported SARS-CoV-2 Infection in United States Coast Guard Personnel between May and August 2021: A Time-Series Analysis. medRxiv 2021.11.19.21266537; doi: https://doi.org/10.1101/2021.11.19.21266537 |
| Jalali, 2022 | Neda Jalali, Hilde K. Brustad, Arnoldo Frigessi, Emily MacDonald, Hinta Meijerink, Siri Feruglio, Karin Nygård, Gunnar Isaksson Rø, Elisabeth H. Madslien, Birgitte Freiesleben De Blasio. Increased household transmission and immune escape of the SARS-CoV-2 Omicron variant compared to the Delta variant: evidence from Norwegian contact tracing and vaccination data. medRxiv 2022.02.07.22270437; doi: https://doi.org/10.1101/2022.02.07.22270437 |
| Jameson, 2021 | Jameson AP, Sebastian T, Jacques LR. Coronavirus disease 2019 (COVID-19) vaccination in healthcare workers: An early real-world experience. Infect Control Hosp Epidemiol. 2021 Apr 16:1-2. doi: 10.1017/ice.2021.171. Epub ahead of print. PMID: 33858531; PMCID: PMC8134897. |
| John, 2021 | John BV, Deng Y, Scheinberg A, Mahmud N, Taddei TH, Kaplan D, Labrada M, Baracco G, Dahman B. Association of BNT162b2 mRNA and mRNA-1273 Vaccines With COVID-19 Infection and Hospitalization Among Patients With Cirrhosis. JAMA Intern Med. 2021 Oct 1;181(10):1306-1314. doi: 10.1001/jamainternmed.2021.4325. PMID: 34254978; PMCID: PMC8278308. |
| John, 2022 | John BV, Deng Y, Schwartz KB, Taddei TH, Kaplan DE, Martin P, Chao HH, Dahman B. Post-Vaccination COVID-19 Infection is Associated with Reduced Mortality in Patients With Cirrhosis. Hepatology. 2022 Jan 12. doi: 10.1002/hep.32337. Epub ahead of print. PMID: 35023206. |
| Johnson, 2022 | Johnson AG, Amin AB, Ali AR, Hoots B, Cadwell BL, Arora S, Avoundjian T, Awofeso AO, Barnes J, Bayoumi NS, Busen K, Chang C, Cima M, Crockett M, Cronquist A, Davidson S, Davis E, Delgadillo J, Dorabawila V, Drenzek C, Eisenstein L, Fast HE, Gent A, Hand J, Hoefer D, Holtzman C, Jara A, Jones A, Kamal-Ahmed I, Kangas S, Kanishka F, Kaur R, Khan S, King J, Kirkendall S, Klioueva A, Kocharian A, Kwon FY, Logan J, Lyons BC, Lyons S, May A, McCormick D; MSHI, Mendoza E, Milroy L, O'Donnell A, Pike M, Pogosjans S, Saupe A, Sell J, Smith E, Sosin DM, Stanislawski E, Steele MK, Stephenson M, Stout A, Strand K, Tilakaratne BP, Turner K, Vest H, Warner S, Wiedeman C, Zaldivar A, Silk BJ, Scobie HM. COVID-19 Incidence and Death Rates Among Unvaccinated and Fully Vaccinated Adults with and Without Booster Doses During Periods of Delta and Omicron Variant Emergence - 25 U.S. Jurisdictions, April 4-December 25, 2021. MMWR Morb Mortal Wkly Rep. 2022 Jan 28;71(4):132-138. doi: 10.15585/mmwr.mm7104e2. PMID: 35085223. |
| June Choe, 2022 | June Choe Y, Yi S, Hwang I, Kim J, Park YJ, Cho E, Jo M, Lee H, Hwa Choi E. Safety and effectiveness of BNT162b2 mRNA Covid-19 vaccine in adolescents. Vaccine. 2022 Jan 31;40(5):691-694. doi: 10.1016/j.vaccine.2021.12.044. Epub 2021 Dec 24. PMID: 35012777; PMCID: PMC8702409. |
| Kale, 2021 | Kale P, Gupta E, Bihari C, Patel N, Rooge S, Pandey A, Bajpai M, Khillan V, Chattopadhyay P, Devi P, Maurya R, Jha N, Mehta P, Kumar M, Sharma P, Saifi S, Swaminathan A, Alam S, Uppili B, Faruq M, Agrawal A, Pandey R, Sarin SK. Vaccine Breakthrough Infections by SARS-CoV-2 Variants after ChAdOx1 nCoV-19 Vaccination in Healthcare Workers. Vaccines (Basel). 2021 Dec 31;10(1):54. doi: 10.3390/vaccines10010054. PMID: 35062715. |
| Katikireddi, 2021 | Katikireddi SV, Cerqueira-Silva T, Vasileiou E, Robertson C, Amele S, Pan J, Taylor B, Boaventura V, Werneck GL, Flores-Ortiz R, Agrawal U, Docherty AB, McCowan C, McMenamin J, Moore E, Ritchie LD, Rudan I, Shah SA, Shi T, Simpson CR, Barreto ML, Oliveira VA, Barral-Netto M, Sheikh A. Two-dose ChAdOx1 nCoV-19 vaccine protection against COVID-19 hospital admissions and deaths over time: a retrospective, population-based cohort study in Scotland and Brazil. Lancet. 2021 Dec 20;399(10319):25–35. doi: 10.1016/S0140-6736(21)02754-9. Epub ahead of print. PMID: 34942103; PMCID: PMC8687670. |
| Katz, 2021 | Katz MA, Harlev EB, Chazan B, Chowers M, Greenberg D, Peretz A, Tshori S, Levy J, Yacobi M, Hirsch A, Amichay D, Weinberger R, Dor AB, Taraday EK, Reznik D, Chayat CB, Sagas D, Zvi HB, Berdinstein R, Rashid G, Avni YS, Mandelboim M, Zuckerman N, Rainy N, Akriv A, Dagan N, Kepten E, Barda N, Balicer RD. Early effectiveness of BNT162b2 Covid-19 vaccine in preventing SARS-CoV-2 infection in healthcare personnel in six Israeli hospitals (CoVEHPI). Vaccine. 2021 Dec 10:S0264-410X(21)01580-2. doi: 10.1016/j.vaccine.2021.11.092. Epub ahead of print. PMID: 34903372; PMCID: PMC8662353. |
| Keegan, 2021 | Keegan LT, Truelove S, Lessler J. Analysis of Vaccine Effectiveness Against COVID-19 and the Emergence of Delta and Other Variants of Concern in Utah. JAMA Netw Open. 2021 Dec 1;4(12):e2140906. doi: 10.1001/jamanetworkopen.2021.40906. PMID: 34940869. |
| Khan, 2021 | Khan N, Mahmud N. Effectiveness of SARS-CoV-2 Vaccination in a Veterans Affairs Cohort of Patients With Inflammatory Bowel Disease With Diverse Exposure to Immunosuppressive Medications. Gastroenterology. 2021 Sep;161(3):827-836. doi: 10.1053/j.gastro.2021.05.044. Epub 2021 May 25. PMID: 34048782; PMCID: PMC8146263. |
| Khan, 2022 | Khan M, Kamran Mushtaq, Muhammad Saddique, Mohd Alghizzawi, Muhammad Yasir, Hafiz Younis, Farah Rashid, Deema AlSoub, Rafie Yakoob, Saad AlKaabi, Khalid Al-Ejji, SAFETY AND EFFECTIVENESS OF THE BNT162B2 MRNA COVID-19 VACCINE IN A NATIONWIDE COHORT OF PATIENTS WITH INFLAMMATORY BOWEL DISEASE, Inflammatory Bowel Diseases, Volume 28, Issue Supplement_1, February 2022, Pages S12–S13, https://doi.org/10.1093/ibd/izac015.019 |
| Kim, 2021 | Kim SS, Chung JR, Belongia EA, McLean HQ, King JP, Nowalk MP, Zimmerman RK, Balasubramani GK, Martin ET, Monto AS, Lamerato LE, Gaglani M, Smith ME, Dunnigan KM, Jackson ML, Jackson LA, Tenforde MW, Verani JR, Kobayashi M, Schrag S, Patel MM, Flannery B. mRNA Vaccine Effectiveness against COVID-19 among Symptomatic Outpatients Aged ≥16 Years in the United States, February - May 2021. J Infect Dis. 2021 Sep 8:jiab451. doi: 10.1093/infdis/jiab451. Epub ahead of print. PMID: 34498052; PMCID: PMC8522410. |
| Kissling, 2021 | Kissling E, Hooiveld M, Sandonis Martín V, Martínez-Baz I, William N, Vilcu AM, Mazagatos C, Domegan L, de Lusignan S, Meijer A, Machado A, Brytting M, Casado I, Murray JK, Belhillil S, Larrauri A, O'Donnell J, Tsang R, de Lange M, Rodrigues AP, Riess M, Castilla J, Hamilton M, Falchi A, Pozo F, Dunford L, Cogdale J, Jansen T, Guiomar R, Enkirch T, Burgui C, Sigerson D, Blanchon T, Martínez Ochoa EM, Connell J, Ellis J, van Gageldonk-Lafeber R, Kislaya I, Rose AM, Valenciano M; I-MOVE-COVID-19 primary care study team; I-MOVE-COVID-19 primary care study team (in addition to authors above). Vaccine effectiveness against symptomatic SARS-CoV-2 infection in adults aged 65 years and older in primary care: I-MOVE-COVID-19 project, Europe, December 2020 to May 2021. Euro Surveill. 2021 Jul;26(29):2100670. doi: 10.2807/1560-7917.ES.2021.26.29.2100670. PMID: 34296676; PMCID: PMC8299744. |
| Lan, 2021 | Fan-Yun Lan, Amalia Sidossis, Eirini Iliaki, Jane Buley, Neetha Nathan, Lou Ann Bruno-Murtha, Stefanos N. Kales. Continued Effectiveness of COVID-19 Vaccination among Urban Healthcare Workers during Delta Variant Predominance. medRxiv 2021.11.15.21265753; doi: https://doi.org/10.1101/2021.11.15.21265753 |
| Larese Filon, 2022 | Larese Filon F, Rui F, Ronchese F, De Michieli P, Negro C. Incidence of COVID-19 infection in hospital workers from March 1, 2020 to May 31, 2021 routinely tested, before and after vaccination with BNT162B2. Sci Rep. 2022 Feb 15;12(1):2533. doi: 10.1038/s41598-021-04665-y. PMID: 35169127; PMCID: PMC8847551. |
| Lauring, 2022 | Adam S. Lauring, Mark W. Tenforde, James D. Chappell, Manjusha Gaglani, Adit A. Ginde, Tresa McNeal, Shekhar Ghamande, David J. Douin, H. Keipp Talbot, Jonathan D. Casey, Nicholas M. Mohr, Anne Zepeski, Nathan I. Shapiro, Kevin W. Gibbs, D. Clark Files, David N. Hager, Arber Shehu, Matthew E. Prekker, Heidi L. Erickson, Matthew C. Exline, Michelle N. Gong, Amira Mohamed, Nicholas J. Johnson, Vasisht Srinivasan, Jay S. Steingrub, Ithan D. Peltan, Samuel M. Brown, Emily T. Martin, Arnold S. Monto, Akram Khan, Catherine L. Hough, Laurence W. Busse, Caitlin C. ten Lohuis, Abhijit Duggal, Jennifer G. Wilson, Alexandra June Gordon, Nida Qadir, Steven Y. Chang, Christopher Mallow, Carolina Rivas, Hilary M. Babcock, Jennie H. Kwon, Natasha Halasa, Carlos G. Grijalva, Todd W. Rice, William B. Stubblefield, Adrienne Baughman, Kelsey N. Womack, Jillian P. Rhoads, Christopher J. Lindsell, Kimberly W. Hart, Yuwei Zhu, Katherine Adams, Stephanie J. Schrag, Samantha M. Olson, Miwako Kobayashi, Jennifer R. Verani, Manish M. Patel, Wesley H. Self, For the Influenza and Other Viruses in the Acutely Ill (IVY) Network. Clinical Severity and mRNA Vaccine Effectiveness for Omicron, Delta, and Alpha SARS-CoV-2 Variants in the United States: A Prospective Observational Study. medRxiv 2022.02.06.22270558; doi: https://doi.org/10.1101/2022.02.06.22270558 |
| Lefèvre, 2021 | Lefèvre B, Tondeur L, Madec Y, Grant R, Lina B, van der Werf S, Rabaud C, Fontanet A. Beta SARS-CoV-2 variant and BNT162b2 vaccine effectiveness in long-term care facilities in France. Lancet Healthy Longev. 2021 Nov;2(11):e685-e687. doi: 10.1016/S2666-7568(21)00230-0. Epub 2021 Sep 22. PMID: 34580665; PMCID: PMC8457759. |
| Lewis, 2021 | Lewis NM, Naioti EA, Self WH, Ginde AA, Douin DJ, Talbot HK, Casey JD, Mohr NM, Zepeski A, Gaglani M, Ghamande SA, McNeal TA, Shapiro NI, Gibbs KW, Files DC, Hager DN, Shehu A, Prekker ME, Erickson HL, Gong MN, Mohamed A, Henning DJ, Steingrub JS, Peltan ID, Brown SM, Martin ET, Hubel K, Hough CL, Busse LW, Ten Lohuis CC, Duggal A, Wilson JG, Gordon AJ, Qadir N, Chang SY, Mallow C, Rivas C, Babcock HM, Kwon JH, Exline MC, Halasa N, Chappell JD, Lauring AS, Grijalva CG, Rice TW, Rhoads JP, Stubblefield WB, Baughman A, Womack KN, Lindsell CJ, Hart KW, Zhu Y, Schrag SJ, Kobayashi M, Verani JR, Patel MM, Tenforde MW. Effectiveness of mRNA vaccines in preventing COVID-19 hospitalization by age and burden of chronic medical conditions among immunocompetent US adults, March-August 2021. J Infect Dis. 2021 Dec 21:jiab619. doi: 10.1093/infdis/jiab619. Epub ahead of print. PMID: 34932114. |
| Lin, 2022 | Lin DY, Gu Y, Wheeler B, Young H, Holloway S, Sunny SK, Moore Z, Zeng D. Effectiveness of Covid-19 Vaccines over a 9-Month Period in North Carolina. N Engl J Med. 2022 Jan 12. doi: 10.1056/NEJMoa2117128. Epub ahead of print. PMID: 35020982. |
| Liu, 2021 | Cong Liu, Junghwan Lee, Casey Ta, Ali Soroush, James R. Rogers, Jae Hyun Kim, Karthik Natarajan, Jason Zucker, Chunhua Weng. A Retrospective Analysis of COVID-19 mRNA Vaccine Breakthrough Infections – Risk Factors and Vaccine Effectiveness. medRxiv 2021.10.05.21264583; doi: https://doi.org/10.1101/2021.10.05.21264583 |
| Lopez Bernal, 2021 | Lopez Bernal J, Andrews N, Gower C, Gallagher E, Simmons R, Thelwall S, Stowe J, Tessier E, Groves N, Dabrera G, Myers R, Campbell CNJ, Amirthalingam G, Edmunds M, Zambon M, Brown KE, Hopkins S, Chand M, Ramsay M. Effectiveness of Covid-19 Vaccines against the B.1.617.2 (Delta) Variant. N Engl J Med. 2021 Aug 12;385(7):585-594. doi: 10.1056/NEJMoa2108891. Epub 2021 Jul 21. PMID: 34289274; PMCID: PMC8314739. |
| Lopez Bernal, 2021 | Lopez Bernal J, Andrews N, Gower C, Robertson C, Stowe J, Tessier E, Simmons R, Cottrell S, Roberts R, O'Doherty M, Brown K, Cameron C, Stockton D, McMenamin J, Ramsay M. Effectiveness of the Pfizer-BioNTech and Oxford-AstraZeneca vaccines on covid-19 related symptoms, hospital admissions, and mortality in older adults in England: test negative case-control study. BMJ. 2021 May 13;373:n1088. doi: 10.1136/bmj.n1088. PMID: 33985964; PMCID: PMC8116636. |
| Lopez Bernal, 2021 | Jamie Lopez Bernal, Nick Andrews, Charlotte Gower, Julia Stowe, Elise Tessier, Ruth Simmons, Mary Ramsay. Effectiveness of BNT162b2 mRNA vaccine and ChAdOx1 adenovirus vector vaccine on mortality following COVID-19. medRxiv 2021.05.14.21257218; doi: https://doi.org/10.1101/2021.05.14.21257218 |
| Lumley, 2021 | Lumley SF, Rodger G, Constantinides B, Sanderson N, Chau KK, Street TL, O'Donnell D, Howarth A, Hatch SB, Marsden BD, Cox S, James T, Warren F, Peck LJ, Ritter TG, de Toledo Z, Warren L, Axten D, Cornall RJ, Jones EY, Stuart DI, Screaton G, Ebner D, Hoosdally S, Chand M, Crook DW, O'Donnell AM, Conlon CP, Pouwels KB, Walker AS, Peto TEA, Hopkins S, Walker TM, Stoesser NE, Matthews PC, Jeffery K, Eyre DW. An observational cohort study on the incidence of SARS-CoV-2 infection and B.1.1.7 variant infection in healthcare workers by antibody and vaccination status. Clin Infect Dis. 2021 Jul 3:ciab608. doi: 10.1093/cid/ciab608. Epub ahead of print. PMID: 34216472. |
| Luong, 2021 | Luong Ngyen LB, Bauer R, Lesieur Z, Galtier F, Duval X, Vanhems P, Lainé F, Tattevin P, Durier C, Launay O; FLUVAC Study group. Vaccine effectiveness against COVID-19 hospitalization in adults in France: A test negative case control study. Infect Dis Now. 2021 Dec 15:S2666-9919(21)00547-9. doi: 10.1016/j.idnow.2021.12.002. Epub ahead of print. PMID: 34920180; PMCID: PMC8673927. |
| Lutrick, 2021 | Lutrick K, Rivers P, Yoo YM, Grant L, Hollister J, Jovel K, Khan S, Lowe A, Baccam Z, Hanson H, Olsho LEW, Fowlkes A, Caban-Martinez AJ, Porter C, Yoon S, Meece J, Gaglani M, Burns J, Mayo Lamberte J, Nakayima Miiro F, Bissonnette A, LeClair L, Kutty PK, Romine JK, Stefanski E, Edwards LJ, Ellingson K, Gerald JK, Bedrick EJ, Madhivanan P, Krupp K, Gerald LB, Thompson M, Burgess JL. Interim Estimate of Vaccine Effectiveness of BNT162b2 (Pfizer-BioNTech) Vaccine in Preventing SARS-CoV-2 Infection Among Adolescents Aged 12-17 Years - Arizona, July-December 2021. MMWR Morb Mortal Wkly Rep. 2021 Dec 31;70(5152):1761-1765. doi: 10.15585/mmwr.mm705152a2. PMID: 34968373; PMCID: PMC8736269. |
| Lytras, 2022 | Theodore Lytras, Flora Kontopidou, Angeliki Lambrou, Sotirios Tsiodras. Comparative effectiveness of COVID-19 vaccination against death and severe disease in an ongoing nationwide mass vaccination campaign. medRxiv 2022.01.28.22270009; doi: https://doi.org/10.1101/2022.01.28.22270009 |
| Macchia, 2021 | Macchia A, Ferrante D, Angeleri P, Biscayart C, Mariani J, Esteban S, Tablado MR, de Quirós FGB. Evaluation of a COVID-19 Vaccine Campaign and SARS-CoV-2 Infection and Mortality Among Adults Aged 60 Years And Older in a Middle-Income Country. JAMA Netw Open. 2021 Oct 1;4(10):e2130800. doi: 10.1001/jamanetworkopen.2021.30800. PMID: 34714342; PMCID: PMC8556631. |
| Maeda, 2022 | Haruka Maeda, Nobuo Saito, Ataru Igarashi, Masayuki Ishida, Kazuya Suami, Ai Yagiuchi, Yuya Kimura, Masaru Komino, Hiromi Arai, Toru Morikawa, Iori Motohashi, Rei Miyazawa, Tetsu Moriyama, Hiroshi Kamura, Mayumi Terada, Osamu Kuwamitsu, Tomoichiro Hayakawa, Eiichiro Sando, Yasuji Ohara, Osamu Teshigawara, Motoi Suzuki, Konosuke Morimoto. Effectiveness of mRNA COVID-19 vaccines against symptomatic SARS-CoV-2 infections during the Delta variant epidemic in Japan: Vaccine Effectiveness Real-time Surveillance for SARS-CoV-2 (VERSUS). medRxiv 2022.01.17.22269394; doi: https://doi.org/10.1101/2022.01.17.22269394 |
| Machado, 2021 | Ausenda Machado, Irina Kislaya, Ana Paula Rodrigues, Duarte Sequeira, João Lima, Camila Cruz, Pedro Pinto Leite, Carlos Matias-Dias, Baltazar Nunes. COVID-19 vaccine effectiveness against laboratory confirmed symptomatic SARS-CoV-2 infection, COVID-19 related hospitalizations and deaths, among individuals aged 65 years or more in Portugal: a cohort study based on data-linkage of national registries February-September 2021. medRxiv 2021.12.10.21267619; doi: https://doi.org/10.1101/2021.12.10.21267619 |
| Mallow, 2022 | Mallow C, Ferreira T, Shukla B, Warde P, Sosa MA, Parekh DJ, Gershengorn HB. Real world SARS-COV-2 vaccine effectiveness in a Miami academic institution. Am J Emerg Med. 2022 Feb 3;54:97-101. doi: 10.1016/j.ajem.2022.01.066. Epub ahead of print. PMID: 35151018. |
| Manley, 2021 | Harold J. Manley, Gideon N. Aweh, Caroline M. Hsu, Daniel E. Weiner, Dana Miskulin, Antonia M. Harford, Doug Johnson, Eduardo K. Lacson. SARS-CoV-2 vaccine effectiveness and breakthrough infections in maintenance dialysis patients. medRxiv 2021.09.24.21264081; doi: https://doi.org/10.1101/2021.09.24.21264081 |
| Martínez-Baz, 2021 | Martínez-Baz I, Miqueleiz A, Casado I, Navascués A, Trobajo-Sanmartín C, Burgui C, Guevara M, Ezpeleta C, Castilla J; Working Group for the Study of COVID-19 in Navarra. Effectiveness of COVID-19 vaccines in preventing SARS-CoV-2 infection and hospitalisation, Navarre, Spain, January to April 2021. Euro Surveill. 2021 May;26(21):2100438. doi: 10.2807/1560-7917.ES.2021.26.21.2100438. PMID: 34047271; PMCID: PMC8161727. |
| Martínez-Baz, 2021 | Martínez-Baz I, Trobajo-Sanmartín C, Miqueleiz A, Guevara M, Fernández-Huerta M, Burgui C, Casado I, Portillo ME, Navascués A, Ezpeleta C, Castilla J; Working Group for the Study of COVID-19 in Navarre; Investigators, other members of the Working Group for the Study of COVID-19 in Navarre. Product-specific COVID-19 vaccine effectiveness against secondary infection in close contacts, Navarre, Spain, April to August 2021. Euro Surveill. 2021 Sep;26(39):2100894. doi: 10.2807/1560-7917.ES.2021.26.39.2100894. PMID: 34596016; PMCID: PMC8485582. |
| Mason, 2021 | Mason TFD, Whitston M, Hodgson J, Watkinson RE, Lau YS, Abdulrazeg O, Sutton M. Effects of BNT162b2 mRNA vaccine on COVID-19 infection and hospitalisation amongst older people: matched case control study for England. BMC Med. 2021 Oct 18;19(1):275. doi: 10.1186/s12916-021-02149-4. PMID: 34663326; PMCID: PMC8523007. |
| Mateo-Urdiales, 2021 | Mateo-Urdiales A, Spila Alegiani S, Fabiani M, Pezzotti P, Filia A, Massari M, Riccardo F, Tallon M, Proietti V, Del Manso M, Puopolo M, Spuri M, Morciano C, D’Ancona FP, Da Cas R, Battilomo S, Bella A, Menniti-Ippolito F; Italian Integrated Surveillance of COVID-19 study group; on behalf of the Italian COVID-19 vaccines registry. Risk of SARS-CoV-2 infection and subsequent hospital admission and death at different time intervals since first dose of COVID-19 vaccine administration, Italy, 27 December 2020 to mid-April 2021. Euro Surveill. 2021 Jun;26(25):2100507. doi: 10.2807/1560-7917.ES.2021.26.25.2100507. PMID: 34169819; PMCID: PMC8229378. |
| Matsuo, 2022 | Matsuo R, Matsumoto N, Kadowaki T, Mitsuhashi T, Takao S, Yorifuji T. Effect of mRNA vaccines in preventing COVID-19 severe pneumonia among COVID-19 patients in Japan. J Epidemiol. 2022 Jan 8. doi: 10.2188/jea.JE20210487. Epub ahead of print. PMID: 35013024. |
| Mattiuzzi, 2022 | Mattiuzzi C, Lippi G. Primary COVID-19 vaccine cycle and booster doses efficacy: analysis of Italian nationwide vaccination campaign. Eur J Public Health. 2022 Jan 3:ckab220. doi: 10.1093/eurpub/ckab220. Epub ahead of print. PMID: 34978571; PMCID: PMC8755380. |
| Mattiuzzi, 2022 | Mattiuzzi C, Lippi G. Efficacy of COVID-19 vaccine booster doses in older people. Eur Geriatr Med. 2022 Jan 24. doi: 10.1007/s41999-022-00615-7. Epub ahead of print. PMID: 35067909. |
| Mazagatos, 2021 | Mazagatos C, Monge S, Olmedo C, Vega L, Gallego P, Martín-Merino E, Sierra MJ, Limia A, Larrauri A; Working group for the surveillance and control of COVID-19 in Spain. Effectiveness of mRNA COVID-19 vaccines in preventing SARS-CoV-2 infections and COVID-19 hospitalisations and deaths in elderly long-term care facility residents, Spain, weeks 53 2020 to 13 2021. Euro Surveill. 2021 Jun;26(24):2100452. doi: 10.2807/1560-7917.ES.2021.26.24.2100452. PMID: 34142647; PMCID: PMC8212595. |
| McEvoy, 2022 | McEvoy CM, Lee A, Misra PS, Lebovic G, Wald R, Yuen DA. Real-world Impact of 2-dose SARS-CoV-2 Vaccination in Kidney Transplant Recipients. Transplantation. 2022 Feb 25. doi: 10.1097/TP.0000000000004081. Epub ahead of print. PMID: 35220385. |
| McKeigue, 2021 | Paul M McKeigue, David A McAllister, Sharon J Hutchinson, Chris Robertson, Diane Stockton, Helen M Colhoun, for the PHS COVID-19 Epidemiology and Research Cell. Efficacy of vaccination against severe COVID-19 in relation to Delta variant and time since second dose: the REACT-SCOT case-control study. medRxiv 2021.09.12.21263448; doi: https://doi.org/10.1101/2021.09.12.21263448 |
| McKeigue, 2021 | Paul M McKeigue, David A McAllister, Chris Robertson, Sharon Hutchinson, Stuart McGurnaghan, Diane Stockton, Helen M Colhoun, for the PHS COVID-19 Epidemiology and Research Cell. Efficacy of two doses of COVID-19 vaccine against severe COVID-19 in those with risk conditions and residual risk to the clinically extremely vulnerable: the REACT-SCOT case-control study. medRxiv 2021.09.13.21262360; doi: https://doi.org/10.1101/2021.09.13.21262360 |
| McLean, 2022 | McLean HQ, McClure DL, King JP, Meece JK, Pattinson D, Neumann G, Kawaoka Y, Rolfes MA, Belongia EA. mRNA COVID-19 vaccine effectiveness against SARS-CoV-2 infection in a prospective community cohort, rural Wisconsin, November 2020 to December 2021. Influenza Other Respir Viruses. 2022 Feb 18. doi: 10.1111/irv.12970. Epub ahead of print. PMID: 35178857. |
| Mendola, 2021 | Mendola M, Tonelli F, Garletti FS, Greco D, Fiscella M, Cucchi I, Costa MC, Carrer P. COVID-19 impact and vaccine effectiveness among healthcare workers of a large University Hospital in Lombardy, Italy. Med Lav. 2021 Dec 23;112(6):453-464. doi: 10.23749/mdl.v112i6.11983. PMID: 34939623. |
| Menni, 2021 | Menni C, Klaser K, May A, Polidori L, Capdevila J, Louca P, Sudre CH, Nguyen LH, Drew DA, Merino J, Hu C, Selvachandran S, Antonelli M, Murray B, Canas LS, Molteni E, Graham MS, Modat M, Joshi AD, Mangino M, Hammers A, Goodman AL, Chan AT, Wolf J, Steves CJ, Valdes AM, Ourselin S, Spector TD. Vaccine side-effects and SARS-CoV-2 infection after vaccination in users of the COVID Symptom Study app in the UK: a prospective observational study. Lancet Infect Dis. 2021 Jul;21(7):939-949. doi: 10.1016/S1473-3099(21)00224-3. Epub 2021 Apr 27. PMID: 33930320; PMCID: PMC8078878. |
| Mirahmadizadeh, 2022 | Alireza Mirahmadizadeh, Alireza Heiran, Kamran Bagheri Lankarani, Mohammadreza Serati, Mohammad Habibi, Owrang Eilami, Fatemeh Heiran, Mohsen Moghadami. Effectiveness of COVID-19 Vaccines in preventing Infectiousness, Hospitalization and Mortality: A Historical Cohort Study Using Iranian Registration Data During Vaccination program. medRxiv 2022.01.18.22269330; doi: https://doi.org/10.1101/2022.01.18.22269330 |
| Moline, 2021 | Moline HL, Whitaker M, Deng L, Rhodes JC, Milucky J, Pham H, Patel K, Anglin O, Reingold A, Chai SJ, Alden NB, Kawasaki B, Meek J, Yousey-Hindes K, Anderson EJ, Farley MM, Ryan PA, Kim S, Nunez VT, Como-Sabetti K, Lynfield R, Sosin DM, McMullen C, Muse A, Barney G, Bennett NM, Bushey S, Shiltz J, Sutton M, Abdullah N, Talbot HK, Schaffner W, Chatelain R, Ortega J, Murthy BP, Zell E, Schrag SJ, Taylor C, Shang N, Verani JR, Havers FP. Effectiveness of COVID-19 Vaccines in Preventing Hospitalization Among Adults Aged ≥65 Years - COVID-NET, 13 States, February-April 2021. MMWR Morb Mortal Wkly Rep. 2021 Aug 13;70(32):1088-1093. doi: 10.15585/mmwr.mm7032e3. PMID: 34383730; PMCID: PMC8360274. |
| Monge, 2021 | Monge S, Olmedo C, Alejos B, Lapeña MF, Sierra MJ, Limia A; COVID-19 Registries Study Group2. Direct and Indirect Effectiveness of mRNA Vaccination against Severe Acute Respiratory Syndrome Coronavirus 2 in Long-Term Care Facilities, Spain. Emerg Infect Dis. 2021 Oct;27(10):2595-2603. doi: 10.3201/eid2710.211184. Epub 2021 Jul 27. PMID: 34314670; PMCID: PMC8462307. |
| Mor, 2021 | Mor O, Zuckerman NS, Hazan I, Fluss R, Ash N, Ginish N, Mendelson E, Alroy-Preis S, Freedman L, Huppert A. BNT162b2 vaccine effectiveness was marginally affected by the SARS-CoV-2 beta variant in fully vaccinated individuals. J Clin Epidemiol. 2021 Oct 29;142:38-44. doi: 10.1016/j.jclinepi.2021.10.011. Epub ahead of print. PMID: 34715314; PMCID: PMC8553421. |
| Morgan, 2022 | Morgan JA, Biggio JR Jr, Martin JK, Mussarat N, Chawla HK, Puri P, Williams FB. Maternal Outcomes After Severe Acute Respiratory Syndrome Coronavirus 2 (SARS-CoV-2) Infection in Vaccinated Compared With Unvaccinated Pregnant Patients. Obstet Gynecol. 2022 Jan 1;139(1):107-109. doi: 10.1097/AOG.0000000000004621. PMID: 34644272. |
| Moustsen-Helms, 2021 | Ida Rask Moustsen-Helms, Hanne-Dorthe Emborg, Jens Nielsen, Katrine Finderup Nielsen, Tyra Grove Krause, Kåre Mølbak, Karina Lauenborg Møller, Ann-Sofie Nicole Berthelsen, Palle Valentiner-Branth. Vaccine effectiveness after 1st and 2nd dose of the BNT162b2 mRNA Covid-19 Vaccine in long-term care facility residents and healthcare workers – a Danish cohort study. medRxiv 2021.03.08.21252200; doi: https://doi.org/10.1101/2021.03.08.21252200 |
| Muhsen, 2021 | Muhsen K, Maimon N, Mizrahi A, Bodenneimer O, Cohen D, Maimon M, Grotto I, Dagan R. Effectiveness of BNT162b2 mRNA COVID-19 vaccine against acquisitions of SARS-CoV-2 among health care workers in long-term care facilities: a prospective cohort study. Clin Infect Dis. 2021 Oct 26:ciab918. doi: 10.1093/cid/ciab918. Epub ahead of print. PMID: 34698808; PMCID: PMC8675294. |
| Muthukrishnan, 2021 | Muthukrishnan J, Vardhan V, Mangalesh S, Koley M, Shankar S, Yadav AK, Khera A. Vaccination status and COVID-19 related mortality: A hospital based cross sectional study. Med J Armed Forces India. 2021 Jul;77(Suppl 2):S278-S282. doi: 10.1016/j.mjafi.2021.06.034. Epub 2021 Jul 26. PMID: 34334894; PMCID: PMC8313045. |
| Nanduri, 2021 | Nanduri S, Pilishvili T, Derado G, Soe MM, Dollard P, Wu H, Li Q, Bagchi S, Dubendris H, Link-Gelles R, Jernigan JA, Budnitz D, Bell J, Benin A, Shang N, Edwards JR, Verani JR, Schrag SJ. Effectiveness of Pfizer-BioNTech and Moderna Vaccines in Preventing SARS-CoV-2 Infection Among Nursing Home Residents Before and During Widespread Circulation of the SARS-CoV-2 B.1.617.2 (Delta) Variant - National Healthcare Safety Network, March 1-August 1, 2021. MMWR Morb Mortal Wkly Rep. 2021 Aug 27;70(34):1163-1166. doi: 10.15585/mmwr.mm7034e3. PMID: 34437519; PMCID: PMC8389386. |
| Nasreen, 2022 | Nasreen S, Chung H, He S, Brown KA, Gubbay JB, Buchan SA, Fell DB, Austin PC, Schwartz KL, Sundaram ME, Calzavara A, Chen B, Tadrous M, Wilson K, Wilson SE, Kwong JC; Canadian Immunization Research Network (CIRN) Provincial Collaborative Network (PCN) Investigators. Effectiveness of COVID-19 vaccines against symptomatic SARS-CoV-2 infection and severe outcomes with variants of concern in Ontario. Nat Microbiol. 2022 Feb 7. doi: 10.1038/s41564-021-01053-0. Epub ahead of print. PMID: 35132198. |
| Ng, 2021 | Ng OT, Koh V, Chiew CJ, Marimuthu K, Thevasagayam NM, Mak TM, Chua JK, Ong SSH, Lim YK, Ferdous Z, Johari AKB, Chen MI, Maurer-Stroh S, Cui L, Lin RTP, Tan KB, Cook AR, Leo PY, Lee PVJ. Impact of Delta Variant and Vaccination on SARS-CoV-2 Secondary Attack Rate Among Household Close Contacts. Lancet Reg Health West Pac. 2021 Dec;17:100299. doi: 10.1016/j.lanwpc.2021.100299. Epub 2021 Nov 1. PMID: 34746899; PMCID: PMC8560026. |
| Nguyen, 2022 | Vincent Grigori Nguyen, Alexei Yavlinsky, Sarah Beale, Susan Hoskins, Vasileios Lampos, Isobel Braithwaite, Thomas E Byrne, Wing Lam Erica Fong, Ellen Fragaszy, Cyril Geismar, Jana Kovar, Annalan M D Navaratnam, Parth Patel, Madhumita Shrotri, Sophie Weber, Andrew C Hayward, Robert W Aldridge. Comparative effectiveness of different primary vaccination courses on mRNA based booster vaccines against SARs-COV-2 infections: A time-varying cohort analysis using trial emulation in the Virus Watch community cohort. medRxiv 2022.02.04.22270479; doi: https://doi.org/10.1101/2022.02.04.22270479 |
| Niessen, 2021 | F.A. Niessen, Mirjam J. Knol, Susan J.M. Hahné, VECTOR study group, M.J.M. Bonten, P.C.J.L. Bruijning-Verhagen. Vaccine effectiveness against COVID-19 related hospital admission in the Netherlands: a test-negative case-control study. medRxiv 2021.11.09.21266060; doi: https://doi.org/10.1101/2021.11.09.21266060 |
| Nordström, 2021 | Nordström P, Ballin M, Nordström A. Effectiveness of heterologous ChAdOx1 nCoV-19 and mRNA prime-boost vaccination against symptomatic Covid-19 infection in Sweden: A nationwide cohort study. Lancet Reg Health Eur. 2021 Dec;11:100249. doi: 10.1016/j.lanepe.2021.100249. Epub 2021 Oct 18. PMID: 34693387; PMCID: PMC8520818. |
| Nordström, 2022 | Nordström P, Ballin M, Nordström A. Risk of infection, hospitalisation, and death up to 9 months after a second dose of COVID-19 vaccine: a retrospective, total population cohort study in Sweden. Lancet. 2022 Feb 26;399(10327):814-823. doi: 10.1016/S0140-6736(22)00089-7. Epub 2022 Feb 4. PMID: 35131043; PMCID: PMC8816388. |
| North, 2021 | North CM, Barczak A, Goldstein RH, Healy BC, Finkelstein DM, Ding DD, Kim A, Boucau J, Shaw B, Gilbert RF, Vyas T, Reynolds Z, Siddle KJ, MacInnis BL, Regan J, Flynn JP, Choudhary MC, Vyas JM, Laskowski K, Dighe AS, Lemieux JE, Li JZ, Baden LR, Siedner MJ, Woolley AE, Sacks CA. Determining the Incidence of Asymptomatic SARS-CoV-2 among Early Recipients of COVID-19 Vaccines: A Prospective Cohort Study of Healthcare Workers before, during and after Vaccination [DISCOVER-COVID-19]. Clin Infect Dis. 2021 Aug 7:ciab643. doi: 10.1093/cid/ciab643. Epub ahead of print. PMID: 34363462; PMCID: PMC8436402. |
| Nunes, 2021 | Nunes B, Rodrigues AP, Kislaya I, Cruz C, Peralta-Santos A, Lima J, Pinto Leite P, Sequeira D, Matias Dias C, Machado A. mRNA vaccine effectiveness against COVID-19-related hospitalisations and deaths in older adults: a cohort study based on data linkage of national health registries in Portugal, February to August 2021. Euro Surveill. 2021 Sep;26(38):2100833. doi: 10.2807/1560-7917.ES.2021.26.38.2100833. PMID: 34558406; PMCID: PMC8462036. |
| Nunes, 2022 | Marta C. Nunes, Sthembile Sibanda, Vicky L. Baillie, Gaurav Kwatra, Ricardo Aguas, Shabir A. Madhi, the Wits VIDA HCW Study Group. SARS-CoV-2 Omicron symptomatic infections in previously infected or vaccinated South African healthcare workers. medRxiv 2022.02.04.22270480; doi: https://doi.org/10.1101/2022.02.04.22270480 |
| Oliveira, 2022 | Oliveira CR, Niccolai LM, Sheikha H, Elmansy L, Kalinich CC, Grubaugh ND, Shapiro ED; Yale SARS-CoV-2 Genomic Surveillance Initiative. Assessment of Clinical Effectiveness of BNT162b2 COVID-19 Vaccine in US Adolescents. JAMA Netw Open. 2022 Mar 1;5(3):e220935. doi: 10.1001/jamanetworkopen.2022.0935. PMID: 35238933; PMCID: PMC8895259. |
| Olson, 2021 | Olson SM, Newhams MM, Halasa NB, Price AM, Boom JA, Sahni LC, Irby K, Walker TC, Schwartz SP, Pannaraj PS, Maddux AB, Bradford TT, Nofziger RA, Boutselis BJ, Cullimore ML, Mack EH, Schuster JE, Gertz SJ, Cvijanovich NZ, Kong M, Cameron MA, Staat MA, Levy ER, Chatani BM, Chiotos K, Zambrano LD, Campbell AP, Patel MM, Randolph AG; Overcoming COVID-19 Investigators. Effectiveness of Pfizer-BioNTech mRNA Vaccination Against COVID-19 Hospitalization Among Persons Aged 12-18 Years - United States, June-September 2021. MMWR Morb Mortal Wkly Rep. 2021 Oct 22;70(42):1483-1488. doi: 10.15585/mmwr.mm7042e1. PMID: 34673751. |
| Olson, 2022 | Olson SM, Newhams MM, Halasa NB, Price AM, Boom JA, Sahni LC, Pannaraj PS, Irby K, Walker TC, Schwartz SP, Maddux AB, Mack EH, Bradford TT, Schuster JE, Nofziger RA, Cameron MA, Chiotos K, Cullimore ML, Gertz SJ, Levy ER, Kong M, Cvijanovich NZ, Staat MA, Kamidani S, Chatani BM, Bhumbra SS, Bline KE, Gaspers MG, Hobbs CV, Heidemann SM, Maamari M, Flori HR, Hume JR, Zinter MS, Michelson KN, Zambrano LD, Campbell AP, Patel MM, Randolph AG; Overcoming Covid-19 Investigators. Effectiveness of BNT162b2 Vaccine against Critical Covid-19 in Adolescents. N Engl J Med. 2022 Jan 12. doi: 10.1056/NEJMoa2117995. Epub ahead of print. PMID: 35021004. |
| Ostropolets, 2021 | Anna Ostropolets, George Hripcsak. COVID-19 vaccination effectiveness rates by week and sources of bias. medRxiv 2021.12.22.21268253; doi: https://doi.org/10.1101/2021.12.22.21268253 |
| Paris, 2021 | Paris C, Perrin S, Hamonic S, Bourget B, Roué C, Brassard O, Tadié E, Gicquel V, Bénézit F, Thibault V, Garlantézec R, Tattevin P. Effectiveness of mRNA-BNT162b2, mRNA-1273, and ChAdOx1 nCoV-19 vaccines against COVID-19 in healthcare workers: an observational study using surveillance data. Clin Microbiol Infect. 2021 Nov;27(11):1699.e5-1699.e8. doi: 10.1016/j.cmi.2021.06.043. Epub 2021 Jul 13. PMID: 34265462; PMCID: PMC8275842. |
| Pascucci, 2021 | Pascucci D, Nurchis MC, Sapienza M, Castrini F, Beccia F, D'Ambrosio F, Grossi A, Castagna C, Pezzullo AM, Zega M, Staiti D, De Simone FM, Mores N, Cambieri A, Vetrugno G, Damiani G, Laurenti P. Evaluation of the Effectiveness and Safety of the BNT162b2 COVID-19 Vaccine in the Vaccination Campaign among the Health Workers of Fondazione Policlinico Universitario Agostino Gemelli IRCCS. Int J Environ Res Public Health. 2021 Oct 22;18(21):11098. doi: 10.3390/ijerph182111098. PMID: 34769618; PMCID: PMC8582885. |
| Pawlowski, 2021 | Pawlowski C, Lenehan P, Puranik A, Agarwal V, Venkatakrishnan AJ, Niesen MJM, O’Horo JC, Virk A, Swift MD, Badley AD, Halamka J, Soundararajan V. FDA-authorized mRNA COVID-19 vaccines are effective per real-world evidence synthesized across a multi-state health system. Med (N Y). 2021 Aug 13;2(8):979-992.e8. doi: 10.1016/j.medj.2021.06.007. Epub 2021 Jun 29. PMID: 34223401; PMCID: PMC8238652. |
| Petráš, 2021 | Petráš M, Lesná IK, Večeřová L, Nyčová E, Malinová J, Klézl P, Nezvedová M, White RE, Máčalík R, Dáňová J, Čelko AM, Adámková V. The Effectiveness of Post-Vaccination and Post-Infection Protection in the Hospital Staff of Three Prague Hospitals: A Cohort Study of 8-Month Follow-Up from the Start of the COVID-19 Vaccination Campaign (COVANESS). Vaccines. 2022; 10(1):9. https://doi.org/10.3390/vaccines10010009 |
| Pilishvili, 2021 | Pilishvili T, Gierke R, Fleming-Dutra KE, Farrar JL, Mohr NM, Talan DA, Krishnadasan A, Harland KK, Smithline HA, Hou PC, Lee LC, Lim SC, Moran GJ, Krebs E, Steele MT, Beiser DG, Faine B, Haran JP, Nandi U, Schrading WA, Chinnock B, Henning DJ, Lovecchio F, Lee J, Barter D, Brackney M, Fridkin SK, Marceaux-Galli K, Lim S, Phipps EC, Dumyati G, Pierce R, Markus TM, Anderson DJ, Debes AK, Lin MY, Mayer J, Kwon JH, Safdar N, Fischer M, Singleton R, Chea N, Magill SS, Verani JR, Schrag SJ; Vaccine Effectiveness among Healthcare Personnel Study Team. Effectiveness of mRNA Covid-19 Vaccine among U.S. Health Care Personnel. N Engl J Med. 2021 Dec 16;385(25):e90. doi: 10.1056/NEJMoa2106599. Epub 2021 Sep 22. PMID: 34551224; PMCID: PMC8482809. |
| Pilishvili, 2021 | Pilishvili T, Fleming-Dutra KE, Farrar JL, Gierke R, Mohr NM, Talan DA, Krishnadasan A, Harland KK, Smithline HA, Hou PC, Lee LC, Lim SC, Moran GJ, Krebs E, Steele M, Beiser DG, Faine B, Haran JP, Nandi U, Schrading WA, Chinnock B, Henning DJ, LoVecchio F, Nadle J, Barter D, Brackney M, Britton A, Marceaux-Galli K, Lim S, Phipps EC, Dumyati G, Pierce R, Markus TM, Anderson DJ, Debes AK, Lin M, Mayer J, Babcock HM, Safdar N, Fischer M, Singleton R, Chea N, Magill SS, Verani J, Schrag S; Vaccine Effectiveness Among Healthcare Personnel Study Team. Interim Estimates of Vaccine Effectiveness of Pfizer-BioNTech and Moderna COVID-19 Vaccines Among Health Care Personnel - 33 U.S. Sites, January-March 2021. MMWR Morb Mortal Wkly Rep. 2021 May 21;70(20):753-758. doi: 10.15585/mmwr.mm7020e2. PMID: 34014909; PMCID: PMC8136422. |
| Polinski, 2021 | Jennifer M. Polinski, Andrew R. Weckstein, Michael Batech, Carly Kabelac, Tripthi Kamath, Raymond Harvey, Sid Jain, Jeremy A. Rassen, Najat Khan, Sebastian Schneeweiss. Effectiveness of the Single-Dose Ad26.COV2.S COVID Vaccine. medRxiv 2021.09.10.21263385; doi: https://doi.org/10.1101/2021.09.10.21263385 |
| Porru, 2022 | Porru S, Spiteri G, Monaco MGL, Valotti A, Carta A, Lotti V, Diani E, Lippi G, Gibellini D, Verlato G. Post-Vaccination SARS-CoV-2 Infections among Health Workers at the University Hospital of Verona, Italy: A Retrospective Cohort Survey. Vaccines (Basel). 2022 Feb 10;10(2):272. doi: 10.3390/vaccines10020272. PMID: 35214733. |
| Poukka, 2022 | Poukka E, Baum U, Palmu AA, Lehtonen TO, Salo H, Nohynek H, Leino T. Cohort study of Covid-19 vaccine effectiveness among healthcare workers in Finland, December 2020 - October 2021. Vaccine. 2022 Jan 31;40(5):701-705. doi: 10.1016/j.vaccine.2021.12.032. Epub 2021 Dec 18. PMID: 34953607; PMCID: PMC8683266. |
| Pouwels, 2021 | Pouwels KB, Pritchard E, Matthews PC, Stoesser N, Eyre DW, Vihta KD, House T, Hay J, Bell JI, Newton JN, Farrar J, Crook D, Cook D, Rourke E, Studley R, Peto TEA, Diamond I, Walker AS. Effect of Delta variant on viral burden and vaccine effectiveness against new SARS-CoV-2 infections in the UK. Nat Med. 2021 Dec;27(12):2127-2135. doi: 10.1038/s41591-021-01548-7. Epub 2021 Oct 14. PMID: 34650248; PMCID: PMC8674129. |
| Powell, 2022 | Annabel A Powell, Freja Kirsebom, Julia Stowe, Kelsey McOwat, Vanessa Saliba, Mary E Ramsay, Jamie Lopez-Bernal, Nick Andrews, Shamez N Ladhani. Adolescent vaccination with BNT162b2 (Comirnaty, Pfizer-BioNTech) vaccine and effectiveness against COVID-19: national test-negative case-control study, England. medRxiv 2021.12.10.21267408; doi: https://doi.org/10.1101/2021.12.10.21267408 |
| Pramod, 2022 | Stuti Pramod, Dhanajayan Govindan, Premkumar Ramasubramani, Sitanshu Sekhar Kar, Rakesh Aggarwal, Nandhini Manoharan, Palanivel Chinnakali, Mahalakshmy Thulasingam, Sonali Sarkar, Molly Mary Thabah. Effectiveness of Covishield vaccine in preventing Covid-19 – A test-negative case-control study. Vaccine, 2022. https://doi.org/10.1016/j.vaccine.2022.02.014. |
| Pritchard, 2021 | Pritchard E, Matthews PC, Stoesser N, Eyre DW, Gethings O, Vihta KD, Jones J, House T, VanSteenHouse H, Bell I, Bell JI, Newton JN, Farrar J, Diamond I, Rourke E, Studley R, Crook D, Peto TEA, Walker AS, Pouwels KB. Impact of vaccination on new SARS-CoV-2 infections in the United Kingdom. Nat Med. 2021 Aug;27(8):1370-1378. doi: 10.1038/s41591-021-01410-w. Epub 2021 Jun 9. PMID: 34108716; PMCID: PMC8363500. |
| Prunas, 2022 | Ottavia Prunas, Daniel M. Weinberger, Virginia E. Pitzer, Sivan Gazit, Tal Patalon. Waning Effectiveness of the BNT162b2 Vaccine Against Infection in Adolescents. medRxiv 2022.01.04.22268776; doi: https://doi.org/10.1101/2022.01.04.22268776 |
| Puranik, 2022 | Puranik A, Lenehan PJ, Silvert E, Niesen MJM, Corchado-Garcia J, O’Horo JC, Virk A, Swift MD, Gordon JE, Speicher LL, Geyer HL, Kremers W, Halamka J, Badley AD, Venkatakrishnan AJ, Soundararajan V. Comparative effectiveness of mRNA-1273 and BNT162b2 against symptomatic SARS-CoV-2 infection. Med (N Y). 2022 Jan 14;3(1):28-41.e8. doi: 10.1016/j.medj.2021.12.002. Epub 2021 Dec 11. PMID: 34927113; PMCID: PMC8664708. |
| Rane, 2022 | Madhura S. Rane, McKaylee Robertson, Sarah Kulkarni, Daniel Frogel, Chris Gainus, Denis Nash. Effectiveness of Covid-19 vaccines against symptomatic and asymptomatic SARS-CoV-2 infections in an urgent care setting. medRxiv 2022.02.21.22271298; doi: https://doi.org/10.1101/2022.02.21.22271298 |
| Ranzani, 2021 | Otavio T Ranzani, Rogério dos Santos Leite, Larissa Domingues Castilho, Crhistinne Cavalheiro Maymone Gonçalves, Geraldo Resende, Rosana Leite de Melo, Julio Croda. Vaccine effectiveness of Ad26.COV2.S against symptomatic COVID-19 and clinical outcomes in Brazil: a test-negative study design. medRxiv 2021.10.15.21265006; doi: https://doi.org/10.1101/2021.10.15.21265006 |
| Ranzani, 2022 | Ranzani OT, Silva AAB, Peres IT, Antunes BBP, Gonzaga-da-Silva TW, Soranz DR, Cerbino-Neto J, Hamacher S, Bozza FA. Vaccine effectiveness of ChAdOx1 nCoV-19 against COVID-19 in a socially vulnerable community in Rio de Janeiro, Brazil: a test-negative design study. Clin Microbiol Infect. 2022 Feb 9:S1198-743X(22)00056-8. doi: 10.1016/j.cmi.2022.01.032. Epub ahead of print. PMID: 35150884. |
| Regev-Yochay, 2021 | Regev-Yochay G, Amit S, Bergwerk M, Lipsitch M, Leshem E, Kahn R, Lustig Y, Cohen C, Doolman R, Ziv A, Novikov I, Rubin C, Gimpelevich I, Huppert A, Rahav G, Afek A, Kreiss Y. Decreased infectivity following BNT162b2 vaccination: A prospective cohort study in Israel. Lancet Reg Health Eur. 2021 Aug;7:100150. doi: 10.1016/j.lanepe.2021.100150. Epub 2021 Jul 7. PMID: 34250518; PMCID: PMC8261633. |
| Reis, 2021 | Reis BY, Barda N, Leshchinsky M, Kepten E, Hernán MA, Lipsitch M, Dagan N, Balicer RD. Effectiveness of BNT162b2 Vaccine against Delta Variant in Adolescents. N Engl J Med. 2021 Nov 25;385(22):2101-2103. doi: 10.1056/NEJMc2114290. Epub 2021 Oct 20. PMID: 34670036; PMCID: PMC8552532. |
| Reynolds, 2022 | Matthew W Reynolds, Alex Secora, Alice Joules, Lisa Albert, Emma Brinkley, Tom Kwon, Christina Mack, Stephen Toovey, Nancy A. Dreyer. Evaluating Real-World COVID-19 Vaccine Effectiveness Using a Test-Negative Case-Control Design. medRxiv 2022.01.06.22268726; doi: https://doi.org/10.1101/2022.01.06.22268726 |
| Risk, 2022 | Risk M, Shen C, Hayek SS, Holevinski L, Schiopu E, Freed G, Akin C, Zhao L. Comparative Effectiveness of COVID-19 Vaccines against the Delta Variant. Clin Infect Dis. 2022 Feb 7:ciac106. doi: 10.1093/cid/ciac106. Epub ahead of print. PMID: 35137006. |
| Roberts, 2022 | Emily K. Roberts, Tian Gu, Bhramar Mukherjee, Lars G. Fritsche. Estimating COVID-19 Vaccination Effectiveness Using Electronic Health Records of an Academic Medical Center in Michigan. medRxiv 2022.01.29.22269971; doi: https://doi.org/10.1101/2022.01.29.22269971 |
| Robilotti, 2021 | Robilotti EV, Whiting K, Lucca A, Poon C, Guest R, McMillen T, Jani K, Solovyov A, Kelson S, Browne K, Freeswick S, Hohl TM, Korenstein D, Ruchnewitz D, Lässig M, Łuksza M, Greenbaum B, Seshan VE, Babady NE, Kamboj M. Clinical and Genomic Characterization of SARS CoV-2 infections in mRNA Vaccinated Health Care Personnel in New York City. Clin Infect Dis. 2021 Oct 13:ciab886. doi: 10.1093/cid/ciab886. Epub ahead of print. PMID: 34644393. |
| Robles-Fontán, 2022 | Robles-Fontán MM, Nieves EG, Cardona-Gerena I, Irizarry RA. Effectiveness estimates of three COVID-19 vaccines based on observational data from Puerto Rico. Lancet Reg Health Am. 2022 May;9:100212. doi: 10.1016/j.lana.2022.100212. Epub 2022 Feb 24. PMID: 35229081; PMCID: PMC8867062. |
| Rosenberg, 2021 | Rosenberg ES, Dorabawila V, Easton D, Bauer UE, Kumar J, Hoen R, Hoefer D, Wu M, Lutterloh E, Conroy MB, Greene D, Zucker HA. Covid-19 Vaccine Effectiveness in New York State. N Engl J Med. 2021 Dec 1:NEJMoa2116063. doi: 10.1056/NEJMoa2116063. Epub ahead of print. PMID: 34942067; PMCID: PMC8693697. |
| Rovida, 2021 | Rovida F, Cassaniti I, Paolucci S, Percivalle E, Sarasini A, Piralla A, Giardina F, Sammartino JC, Ferrari A, Bergami F, Muzzi A, Novelli V, Meloni A, Cutti S, Grugnetti AM, Grugnetti G, Rona C, Daglio M, Marena C, Triarico A, Lilleri D, Baldanti F. SARS-CoV-2 vaccine breakthrough infections with the alpha variant are asymptomatic or mildly symptomatic among health care workers. Nat Commun. 2021 Oct 15;12(1):6032. doi: 10.1038/s41467-021-26154-6. PMID: 34654808; PMCID: PMC8521593. |
| Rudolph, 2021 | Rudolph JL, Hartronft S, McConeghy K, Kennedy M, Intrator O, Minor L, Hubert TL, Goldstein MK. Proportion of SARS-CoV-2 positive tests and vaccination in Veterans Affairs Community Living Centers. J Am Geriatr Soc. 2021 Aug;69(8):2090-2095. doi: 10.1111/jgs.17180. Epub 2021 May 4. PMID: 33861871; PMCID: PMC8250473. |
| Saciuk, 2021 | Saciuk Y, Kertes J, Mandel M, Hemo B, Shamir Stein N, Ekka Zohar A. Pfizer-BioNTech vaccine effectiveness against Sars-Cov-2 infection: Findings from a large observational study in Israel. Prev Med. 2021 Dec 30;155:106947. doi: 10.1016/j.ypmed.2021.106947. Epub ahead of print. PMID: 34974072; PMCID: PMC8717697. |
| Sagiraju, 2021 | Hari Krishna Raju Sagiraju, Arunmozhimaran Elavarasi, Nishkarsh Gupta, Rohit Kumar Garg, Saurav Sekhar Paul, Saurabh Vig, Prashant Sirohiya, Brajesh Ratre, Rakesh Garg, Anuja Pandit, Ram Nalwa, Balbir Kumar, Ved Prakash Meena, Naveet Wig, Saurabh Mittal, Sourabh Pahuja, Karan Madan, Nupur Das, Tanima Dwivedi, Ritu Gupta, Laxmitej Wundawalli, Angel Rajan Singh, Sheetal Singh, Abhinav Mishra, Manisha Pandey, Karanvir Singh Matharoo, Sunil Kumar, Anant Mohan, Randeep Guleria, Sushma Bhatnagar. The effectiveness of SARS-CoV-2 vaccination in preventing severe illness and death – real-world data from a cohort of patients hospitalized with COVID-19. medRxiv 2021.08.26.21262705; doi: https://doi.org/10.1101/2021.08.26.21262705 |
| Satwik, 2021 | Satwik R, Satwik A, Katoch S, Saluja S. ChAdOx1 nCoV-19 effectiveness during an unprecedented surge in SARS COV-2 infections. Eur J Intern Med. 2021 Nov;93:112-113. doi: 10.1016/j.ejim.2021.08.005. Epub 2021 Aug 16. PMID: 34419309; PMCID: PMC8364816. |
| Self, 2021 | Self WH, Tenforde MW, Rhoads JP, Gaglani M, Ginde AA, Douin DJ, Olson SM, Talbot HK, Casey JD, Mohr NM, Zepeski A, McNeal T, Ghamande S, Gibbs KW, Files DC, Hager DN, Shehu A, Prekker ME, Erickson HL, Gong MN, Mohamed A, Henning DJ, Steingrub JS, Peltan ID, Brown SM, Martin ET, Monto AS, Khan A, Hough CL, Busse LW, Ten Lohuis CC, Duggal A, Wilson JG, Gordon AJ, Qadir N, Chang SY, Mallow C, Rivas C, Babcock HM, Kwon JH, Exline MC, Halasa N, Chappell JD, Lauring AS, Grijalva CG, Rice TW, Jones ID, Stubblefield WB, Baughman A, Womack KN, Lindsell CJ, Hart KW, Zhu Y, Mills L, Lester SN, Stumpf MM, Naioti EA, Kobayashi M, Verani JR, Thornburg NJ, Patel MM; IVY Network. Comparative Effectiveness of Moderna, Pfizer-BioNTech, and Janssen (Johnson & Johnson) Vaccines in Preventing COVID-19 Hospitalizations Among Adults Without Immunocompromising Conditions - United States, March-August 2021. MMWR Morb Mortal Wkly Rep. 2021 Sep 24;70(38):1337-1343. doi: 10.15585/mmwr.mm7038e1. PMID: 34555004; PMCID: PMC8459899. |
| Seppälä, 2021 | Seppälä E, Veneti L, Starrfelt J, Danielsen AS, Bragstad K, Hungnes O, Taxt AM, Watle SV, Meijerink H. Vaccine effectiveness against infection with the Delta (B.1.617.2) variant, Norway, April to August 2021. Euro Surveill. 2021 Sep;26(35):2100793. doi: 10.2807/1560-7917.ES.2021.26.35.2100793. PMID: 34477054; PMCID: PMC8414959. |
| Shah, 2021 | Shah ASV, Gribben C, Bishop J, Hanlon P, Caldwell D, Wood R, Reid M, McMenamin J, Goldberg D, Stockton D, Hutchinson S, Robertson C, McKeigue PM, Colhoun HM, McAllister DA. Effect of Vaccination on Transmission of SARS-CoV-2. N Engl J Med. 2021 Oct 28;385(18):1718-1720. doi: 10.1056/NEJMc2106757. Epub 2021 Sep 8. PMID: 34496200; PMCID: PMC8451182. |
| Sharma, 2021 | Aditya Sharma, Gina Oda, Mark Holodniy. Effectiveness of a third dose of BNT162b2 or mRNA-1273 vaccine for preventing post-vaccination COVID-19 infection: an observational study. medRxiv 2021.11.29.21266777; doi: https://doi.org/10.1101/2021.11.29.21266777 |
| Sheikh, 2021 | Sheikh A, Robertson C, Taylor B. BNT162b2 and ChAdOx1 nCoV-19 Vaccine Effectiveness against Death from the Delta Variant. N Engl J Med. 2021 Dec 2;385(23):2195-2197. doi: 10.1056/NEJMc2113864. Epub 2021 Oct 20. PMID: 34670038; PMCID: PMC8552534. |
| Sheikh, 2021 | Sheikh A, McMenamin J, Taylor B, Robertson C; Public Health Scotland and the EAVE II Collaborators. SARS-CoV-2 Delta VOC in Scotland: demographics, risk of hospital admission, and vaccine effectiveness. Lancet. 2021 Jun 26;397(10293):2461-2462. doi: 10.1016/S0140-6736(21)01358-1. Epub 2021 Jun 14. PMID: 34139198; PMCID: PMC8201647. |
| Shen, 2022 | Shen C, Risk M, Schiopu E, Hayek SS, Xie T, Holevinski L, Akin C, Freed G, Zhao L. Efficacy of COVID-19 vaccines in patients taking immunosuppressants. Ann Rheum Dis. 2022 Feb 23:annrheumdis-2021-222045. doi: 10.1136/annrheumdis-2021-222045. Epub ahead of print. PMID: 35197265. |
| Shrestha, 2021 | Nabin K. Shrestha, Amy S. Nowacki, Patrick C. Burke, Paul Terpeluk, Steven M. Gordon. Effectiveness of mRNA COVID-19 Vaccines among Employees in an American Healthcare System. medRxiv 2021.06.02.21258231; doi: https://doi.org/10.1101/2021.06.02.21258231 |
| Shrestha, 2022 | Shrestha NK, Burke PC, Nowacki AS, Terpeluk P, Gordon SM. Necessity of COVID-19 Vaccination in Persons Who Have Already Had COVID-19. Clin Infect Dis. 2022 Jan 13:ciac022. doi: 10.1093/cid/ciac022. Epub ahead of print. PMID: 35028662. |
| Shrotri, 2021 | Shrotri M, Krutikov M, Palmer T, Giddings R, Azmi B, Subbarao S, Fuller C, Irwin-Singer A, Davies D, Tut G, Lopez Bernal J, Moss P, Hayward A, Copas A, Shallcross L. Vaccine effectiveness of the first dose of ChAdOx1 nCoV-19 and BNT162b2 against SARS-CoV-2 infection in residents of long-term care facilities in England (VIVALDI): a prospective cohort study. Lancet Infect Dis. 2021 Nov;21(11):1529-1538. doi: 10.1016/S1473-3099(21)00289-9. Epub 2021 Jun 23. PMID: 34174193; PMCID: PMC8221738. |
| Schiavetti, 2022 | Irene Schiavetti, Cinzia Cordioli, Maria Laura Stromillo, Maria Teresa Ferrò, Alice Laroni, Eleonora Cocco, Gaia Cola, Livia Pasquali, Maria Teresa Rilla, Elisabetta Signoriello, Rosa Iodice, Alessia Di Sapio, Roberta Lanzillo, Francesca Caleri, Pietro Annovazzi, Antonella Conte, Giuseppe Liberatore, Francesca Ruscica, Renato Docimo, Simona Bonavita, Monica Ulivelli, Paola Cavalla, Francesco Patti, Diana Ferraro, Marinella Clerico, Paolo Immovilli, Massimiliano Di Filippo, Marco Salvetti, Maria Pia Sormani, the “Breakthrough infections in MS” study group. Breakthrough SARS-CoV-2 infections in MS patients on disease modifying therapies. medRxiv 2022.01.22.22269630; doi: https://doi.org/10.1101/2022.01.22.22269630 |
| Sibbel, 2021 | Sibbel S, McKeon K, Luo J, Wendt K, Walker AG, Kelley T, Lazar R, Zywno ML, Connaire JJ, Tentori F, Young A, Brunelli SM. Real-World Effectiveness and Immunogenicity of BNT162b2 and mRNA-1273 SARS-CoV-2 Vaccines in Patients on Hemodialysis. J Am Soc Nephrol. 2022 Jan;33(1):49-57. doi: 10.1681/ASN.2021060778. Epub 2021 Nov 17. PMID: 34789546. |
| Singer, 2021 | Singer SR, Angulo FJ, Swerdlow DL, McLaughlin JM, Hazan I, Ginish N, Anis E, Mendelson E, Mor O, Zuckerman NS, Erster O, Southern J, Pan K, Mircus G, Lipsitch M, Haas EJ, Jodar L, Levy Y, Alroy-Preis S. Effectiveness of BNT162b2 mRNA COVID-19 vaccine against SARS-CoV-2 variant Beta (B.1.351) among persons identified through contact tracing in Israel: A prospective cohort study. EClinicalMedicine. 2021 Dec;42:101190. doi: 10.1016/j.eclinm.2021.101190. Epub 2021 Nov 29. PMID: 34870134; PMCID: PMC8628463. |
| Singh, 2021 | Singh C, Naik BN, Pandey S, Biswas B, Pati BK, Verma M, Singh PK. Effectiveness of COVID-19 vaccine in preventing infection and disease severity: a case-control study from an Eastern State of India. Epidemiol Infect. 2021 Oct 11;149:e224. doi: 10.1017/S0950268821002247. PMID: 34632972; PMCID: PMC8545845. |
| Skowronski, 2021 | Skowronski DM, Setayeshgar S, Zou M, Prystajecky N, Tyson JR, Galanis E, Naus M, Patrick DM, Sbihi H, El Adam S, Henry B, Hoang LMN, Sadarangani M, Jassem AN, Krajden M. Single-dose mRNA vaccine effectiveness against SARS-CoV-2, including Alpha and Gamma variants: a test-negative design in adults 70 years and older in British Columbia, Canada. Clin Infect Dis. 2021 Jul 9:ciab616. doi: 10.1093/cid/ciab616. Epub ahead of print. PMID: 34244723; PMCID: PMC8406884. |
| Skowronski, 2021 | Danuta M Skowronski, Solmaz Setayeshgar, Yossi Febriani, Manale Ouakki, Macy Zou, Denis Talbot, Natalie Prystajecky, John R Tyson, Rodica Gilca, Nicholas Brousseau, Geneviève Deceuninck, Eleni Galanis, Chris D Fjell, Hind Sbihi, Elise Fortin, Sapha Barkati, Chantal Sauvageau, Monika Naus, David M Patrick, Bonnie Henry, Linda M N Hoang, Philippe De Wals, Christophe Garenc, Alex Carignan, Mélanie Drolet, Manish Sadarangani, Marc Brisson, Mel Krajden, Gaston De Serres. Two-dose SARS-CoV-2 vaccine effectiveness with mixed schedules and extended dosing intervals: test-negative design studies from British Columbia and Quebec, Canada. medRxiv 2021.10.26.21265397; doi: https://doi.org/10.1101/2021.10.26.21265397 |
| Skowronski, 2022 | Skowronski DM, Setayeshgar S, Zou M, Prystajecky N, Tyson JR, Sbihi H, Fjell CD, Galanis E, Naus M, Patrick DM, El Adam S, Ahmed MA, Kim S, Henry B, Hoang LMN, Sadarangani M, Jassem AN, Krajden M. Comparative single-dose mRNA and ChAdOx1 vaccine effectiveness against SARS-CoV-2, including variants of concern: test-negative design, British Columbia, Canada. J Infect Dis. 2022 Jan 27:jiac023. doi: 10.1093/infdis/jiac023. Epub ahead of print. PMID: 35084500. |
| Spensley, 2022 | Katrina Spensley, Sarah Gleeson, Paul Martin, Tina Thomson, Candice L. Clarke, Graham Pickard, David Thomas, Stephen P. McAdoo, Paul Randell, Peter Kelleher, Rachna Bedi, Liz Lightstone, Maria Prendecki, Michelle Willicombe. Comparison of vaccine effectiveness against the Omicron (B.1.1.529) variant in patients receiving haemodialysis. medRxiv 2022.01.25.22269804; doi: https://doi.org/10.1101/2022.01.25.22269804 |
| Sritipsukho, 2022 | Sritipsukho P MD, MSc, Khawcharoenporn T MD, MSc, Siribumrungwong B MD, PhD, Damronglerd P MD, Suwantarat N MD, Satdhabudha A MD, Chaiyakulsil C MD, Sinlapamongkolkul P MD, Tangsathapornpong A MD, Bunjoungmanee P MD, Nanthapisal S MD, PhD, Tanprasertkul C MD, PhD, Sritipsukho N PhD, Mingmalairak C MD, Apisarnthanarak A MD, Tantiyavarong P MD, PhD. Comparing real-life effectiveness of various COVID-19 vaccine regimens during the delta variant-dominant pandemic: A test-negative case-control study. Emerg Microbes Infect. 2022 Feb 3:1-22. doi: 10.1080/22221751.2022.2037398. Epub ahead of print. PMID: 35114893. |
| Starrfelt, 2021 | Jostein Starrfelt, Eirik Alnes Buanes, Lene Kristine Juvet, Trude Marie Lyngstad, Gunnar Øyvind Isaksson Rø, Lamprini Veneti, Hinta Meijerink. Age and product dependent vaccine effectiveness against SARS-CoV-2 infection and hospitalisation among adults in Norway: a national cohort study, January – September 2021. medRxiv 2021.11.12.21266222; doi: https://doi.org/10.1101/2021.11.12.21266222 |
| Starrfelt, 2021 | Jostein Starrfelt, Anders S Danielsen, Oliver Kacelnik, Anita Wang Børseth, Elina Seppälä, Hinta Meijerink. High vaccine effectiveness against COVID-19 infection and severe disease among residents and staff of long-term care facilities in Norway, November – June 2021. medRxiv 2021.08.08.21261357; doi: https://doi.org/10.1101/2021.08.08.21261357 |
| Suah, 2021 | Suah JL, Tok PSK, Ong SM, Husin M, Tng BH, Sivasampu S, Thevananthan T, Appannan MR, Muhamad Zin F, Mohd Zin S, Yahaya H, Rusli N, Ujang MF, Mohd Ibrahim H, Abdullah NH, Peariasamy KM. PICK-ing Malaysia’s Epidemic Apart: Effectiveness of a Diverse COVID-19 Vaccine Portfolio. Vaccines (Basel). 2021 Nov 24;9(12):1381. doi: 10.3390/vaccines9121381. PMID: 34960126; PMCID: PMC8706086. |
| Suah, 2022 | Jing Lian Suah, Masliyana Husin, Peter Seah Keng Tok, Boon Hwa Tng, Thevesh Thevananthan, Ee Vien Low, Maheshwara Rao Appannan, Faizah Muhamad Zin, Shahanizan Mohd Zin, Hazlina Yahaya, Kalaiarasu M. Peariasamy, Sheamini Sivasampu. Waning COVID-19 Vaccine Effectiveness for BNT162b2 and CoronaVac in Malaysia: An Observational Study. medRxiv 2022.01.15.22269326; doi: https://doi.org/10.1101/2022.01.15.22269326 |
| Sultan, 2022 | Iyad Sultan, Abdelghani Tbakhi, Osama Abuatta, Sawsan Mubarak, Osama Alsmadi, Adib Edilbi, Ruba Al-Ani, Manar Makhlouf, Rawan Hajir, Omar Khreisat, Majeda A. Al-Ruzzieh, Hikmat Abdelrazeq, Asem Mansour. Distinct Vaccine Efficacy Rates Among Health Care Workers During a COVID-19 Outbreak in Jordan. medRxiv 2022.01.15.22269356; doi: https://doi.org/10.1101/2022.01.15.22269356 |
| Swift, 2021 | Swift MD, Breeher LE, Tande AJ, Tommaso CP, Hainy CM, Chu H, Murad MH, Berbari EF, Virk A. Effectiveness of Messenger RNA Coronavirus Disease 2019 (COVID-19) Vaccines Against Severe Acute Respiratory Syndrome Coronavirus 2 (SARS-CoV-2) Infection in a Cohort of Healthcare Personnel. Clin Infect Dis. 2021 Sep 15;73(6):e1376-e1379. doi: 10.1093/cid/ciab361. PMID: 33900384; PMCID: PMC8135611. |
| Syed, 2022 | Syed MA Dr, A/Qotba HA Dr, Al Nuaimi AS Dr. Effectiveness of COVID-19 vaccines in Qatar. J Infect. 2022 Mar 1:S0163-4453(22)00121-9. doi: 10.1016/j.jinf.2022.02.034. Epub ahead of print. PMID: 35245580; PMCID: PMC8889880. |
| Šmíd, 2022 | Martin Šmíd, Luděk Berec, Ondřej Májek, Tomáš Pavlík, Jiří Jarkovský, Jakub Weiner, Lenka Přibylová, Tamara Barusová, Jan Trnka. Protection by vaccines and previous infection against the Omicron variant of SARS-CoV-2. medRxiv 2022.02.24.22271396; doi: https://doi.org/10.1101/2022.02.24.22271396 |
| Tabak, 2021 | Tabak YP, Sun X, Brennan TA, Chaguturu SK. Incidence and Estimated Vaccine Effectiveness Against Symptomatic SARS-CoV-2 Infection Among Persons Tested in US Retail Locations, May 1 to August 7, 2021. JAMA Netw Open. 2021 Dec 1;4(12):e2143346. doi: 10.1001/jamanetworkopen.2021.43346. PMID: 34935923. |
| Tande, 2021 | Tande AJ, Pollock BD, Shah ND, Farrugia G, Virk A, Swift M, Breeher L, Binnicker M, Berbari EF. Impact of the COVID-19 Vaccine on Asymptomatic Infection Among Patients Undergoing Pre-Procedural COVID-19 Molecular Screening. Clin Infect Dis. 2021 Mar 10:ciab229. doi: 10.1093/cid/ciab229. Epub ahead of print. PMID: 33704435; PMCID: PMC7989519. |
| Tande, 2021 | Tande AJ, Pollock BD, Shah ND, Binnicker M, Berbari EF. mRNA vaccine effectiveness against asymptomatic severe acute respiratory coronavirus virus 2 (SARS-CoV-2) infection over seven months. Infect Control Hosp Epidemiol. 2021 Sep 6:1-3. doi: 10.1017/ice.2021.399. Epub ahead of print. PMID: 34486511; PMCID: PMC8723985. |
| Tang, 2021 | Tang L, Hijano DR, Gaur AH, Geiger TL, Neufeld EJ, Hoffman JM, Hayden RT. Asymptomatic and Symptomatic SARS-CoV-2 Infections After BNT162b2 Vaccination in a Routinely Screened Workforce. JAMA. 2021 Jun 22;325(24):2500-2502. doi: 10.1001/jama.2021.6564. PMID: 33956050; PMCID: PMC8220512. |
| Tang, 2021 | Tang P, Hasan MR, Chemaitelly H, Yassine HM, Benslimane FM, Al Khatib HA, AlMukdad S, Coyle P, Ayoub HH, Al Kanaani Z, Al Kuwari E, Jeremijenko A, Kaleeckal AH, Latif AN, Shaik RM, Abdul Rahim HF, Nasrallah GK, Al Kuwari MG, Al Romaihi HE, Butt AA, Al-Thani MH, Al Khal A, Bertollini R, Abu-Raddad LJ. BNT162b2 and mRNA-1273 COVID-19 vaccine effectiveness against the SARS-CoV-2 Delta variant in Qatar. Nat Med. 2021 Dec;27(12):2136-2143. doi: 10.1038/s41591-021-01583-4. Epub 2021 Nov 2. PMID: 34728831. |
| Tartof, 2021 | Tartof SY, Slezak JM, Fischer H, Hong V, Ackerson BK, Ranasinghe ON, Frankland TB, Ogun OA, Zamparo JM, Gray S, Valluri SR, Pan K, Angulo FJ, Jodar L, McLaughlin JM. Effectiveness of mRNA BNT162b2 COVID-19 vaccine up to 6 months in a large integrated health system in the USA: a retrospective cohort study. Lancet. 2021 Oct 16;398(10309):1407-1416. doi: 10.1016/S0140-6736(21)02183-8. Epub 2021 Oct 4. PMID: 34619098; PMCID: PMC8489881. |
| Tartof, 2022 | Tartof SY, Slezak JM, Puzniak L, Hong V, Frankland TB, Ackerson BK, Takhar HS, Ogun OA, Simmons SR, Zamparo JM, Gray S, Valluri SR, Pan K, Jodar L, McLaughlin JM. Effectiveness of a third dose of BNT162b2 mRNA COVID-19 vaccine in a large US health system: A retrospective cohort study. Lancet Reg Health Am. 2022 Feb 14:100198. doi: 10.1016/j.lana.2022.100198. Epub ahead of print. PMID: 35187521; PMCID: PMC8841530. |
| Tenforde, 2021 | Tenforde MW, Olson SM, Self WH, Talbot HK, Lindsell CJ, Steingrub JS, Shapiro NI, Ginde AA, Douin DJ, Prekker ME, Brown SM, Peltan ID, Gong MN, Mohamed A, Khan A, Exline MC, Files DC, Gibbs KW, Stubblefield WB, Casey JD, Rice TW, Grijalva CG, Hager DN, Shehu A, Qadir N, Chang SY, Wilson JG, Gaglani M, Murthy K, Calhoun N, Monto AS, Martin ET, Malani A, Zimmerman RK, Silveira FP, Middleton DB, Zhu Y, Wyatt D, Stephenson M, Baughman A, Womack KN, Hart KW, Kobayashi M, Verani JR, Patel MM; IVY Network; HAIVEN Investigators. Effectiveness of Pfizer-BioNTech and Moderna Vaccines Against COVID-19 Among Hospitalized Adults Aged ≥65 Years - United States, January-March 2021. MMWR Morb Mortal Wkly Rep. 2021 May 7;70(18):674-679. doi: 10.15585/mmwr.mm7018e1. PMID: 33956782. |
| Tenforde, 2021 | Tenforde MW, Self WH, Adams K, Gaglani M, Ginde AA, McNeal T, Ghamande S, Douin DJ, Talbot HK, Casey JD, Mohr NM, Zepeski A, Shapiro NI, Gibbs KW, Files DC, Hager DN, Shehu A, Prekker ME, Erickson HL, Exline MC, Gong MN, Mohamed A, Henning DJ, Steingrub JS, Peltan ID, Brown SM, Martin ET, Monto AS, Khan A, Hough CL, Busse LW, Ten Lohuis CC, Duggal A, Wilson JG, Gordon AJ, Qadir N, Chang SY, Mallow C, Rivas C, Babcock HM, Kwon JH, Halasa N, Chappell JD, Lauring AS, Grijalva CG, Rice TW, Jones ID, Stubblefield WB, Baughman A, Womack KN, Rhoads JP, Lindsell CJ, Hart KW, Zhu Y, Olson SM, Kobayashi M, Verani JR, Patel MM; Influenza and Other Viruses in the Acutely Ill (IVY) Network. Association Between mRNA Vaccination and COVID-19 Hospitalization and Disease Severity. JAMA. 2021 Nov 23;326(20):2043-2054. doi: 10.1001/jama.2021.19499. PMID: 34734975; PMCID: PMC8569602. |
| Tenforde, 2021 | Tenforde MW, Self WH, Naioti EA, Ginde AA, Douin DJ, Olson SM, Talbot HK, Casey JD, Mohr NM, Zepeski A, Gaglani M, McNeal T, Ghamande S, Shapiro NI, Gibbs KW, Files DC, Hager DN, Shehu A, Prekker ME, Erickson HL, Gong MN, Mohamed A, Henning DJ, Steingrub JS, Peltan ID, Brown SM, Martin ET, Monto AS, Khan A, Hough CL, Busse LW, Ten Lohuis CC, Duggal A, Wilson JG, Gordon AJ, Qadir N, Chang SY, Mallow C, Rivas C, Babcock HM, Kwon JH, Exline MC, Halasa N, Chappell JD, Lauring AS, Grijalva CG, Rice TW, Jones ID, Stubblefield WB, Baughman A, Womack KN, Lindsell CJ, Hart KW, Zhu Y, Stephenson M, Schrag SJ, Kobayashi M, Verani JR, Patel MM; IVY Network Investigators; IVY Network. Sustained Effectiveness of Pfizer-BioNTech and Moderna Vaccines Against COVID-19 Associated Hospitalizations Among Adults - United States, March-July 2021. MMWR Morb Mortal Wkly Rep. 2021 Aug 27;70(34):1156-1162. doi: 10.15585/mmwr.mm7034e2. PMID: 34437524; PMCID: PMC8389395. |
| Tenforde, 2021 | Mark W Tenforde, Manish M Patel, Adit A Ginde, David J Douin, H Keipp Talbot, Jonathan D Casey, Nicholas M Mohr, Anne Zepeski, Manjusha Gaglani, Tresa McNeal, Shekhar Ghamande, Nathan I Shapiro, Kevin W Gibbs, D Clark Files, David N Hager, Arber Shehu, Matthew E Prekker, Heidi L Erickson, Matthew C Exline, Michelle N Gong, Amira Mohamed, Daniel J Henning, Jay S Steingrub, Ithan D Peltan, Samuel M Brown, Emily T Martin, Arnold S Monto, Akram Khan, Catherine L Hough, Laurence W Busse, Caitlin C ten Lohuis, Abhijit Duggal, Jennifer G Wilson, Alexandra June Gordon, Nida Qadir, Steven Y Chang, Christopher Mallow, Hayley B Gershengorn, Hilary M Babcock, Jennie H Kwon, Natasha Halasa, James D Chappell, Adam S Lauring, Carlos G Grijalva, Todd W Rice, Ian D Jones, William B Stubblefield, Adrienne Baughman, Kelsey N Womack, Christopher J Lindsell, Kimberly W Hart, Yuwei Zhu, Samantha M Olson, Meagan Stephenson, Stephanie J Schrag, Miwako Kobayashi, Jennifer R Verani, Wesley H Self, Influenza and Other Viruses in the Acutely Ill (IVY) Network, Effectiveness of Severe Acute Respiratory Syndrome Coronavirus 2 Messenger RNA Vaccines for Preventing Coronavirus Disease 2019 Hospitalizations in the United States, Clinical Infectious Diseases, 2021, ciab687, https://doi.org/10.1093/cid/ciab687 |
| Tenforde, 2022 | Tenforde MW, Patel MM, Gaglani M, Ginde AA, Douin DJ, Talbot HK, Casey JD, Mohr NM, Zepeski A, McNeal T, Ghamande S, Gibbs KW, Files DC, Hager DN, Shehu A, Prekker ME, Erickson HL, Gong MN, Mohamed A, Johnson NJ, Srinivasan V, Steingrub JS, Peltan ID, Brown SM, Martin ET, Monto AS, Khan A, Hough CL, Busse LW, Duggal A, Wilson JG, Qadir N, Chang SY, Mallow C, Rivas C, Babcock HM, Kwon JH, Exline MC, Botros M, Lauring AS, Shapiro NI, Halasa N, Chappell JD, Grijalva CG, Rice TW, Jones ID, Stubblefield WB, Baughman A, Womack KN, Rhoads JP, Lindsell CJ, Hart KW, Zhu Y, Naioti EA, Adams K, Lewis NM, Surie D, McMorrow ML, Self WH; IVY Network. Effectiveness of a Third Dose of Pfizer-BioNTech and Moderna Vaccines in Preventing COVID-19 Hospitalization Among Immunocompetent and Immunocompromised Adults - United States, August-December 2021. MMWR Morb Mortal Wkly Rep. 2022 Jan 28;71(4):118-124. doi: 10.15585/mmwr.mm7104a2. PMID: 35085218. |
| Thakkar, 2022 | Thakkar PV, Zimmerman KO, Brookhart MA, Erickson TR, Benjamin DK Jr, Kalu IC. COVID-19 Incidence Among 6th-12th Grade Students by Vaccination Status. Pediatrics. 2022 Feb 22. doi: 10.1542/peds.2022-056230. Epub ahead of print. PMID: 35190835. |
| Thiruvengadam, 2021 | Thiruvengadam R, Awasthi A, Medigeshi G, Bhattacharya S, Mani S, Sivasubbu S, Shrivastava T, Samal S, Rathna Murugesan D, Koundinya Desiraju B, Kshetrapal P, Pandey R, Scaria V, Kumar Malik P, Taneja J, Binayke A, Vohra T, Zaheer A, Rathore D, Ahmad Khan N, Shaman H, Ahmed S, Kumar R, Deshpande S, Subramani C, Wadhwa N, Gupta N, Pandey AK, Bhattacharya J, Agrawal A, Vrati S, Bhatnagar S, Garg PK; Department of Biotechnology India Consortium for COVID-19 research. Effectiveness of ChAdOx1 nCoV-19 vaccine against SARS-CoV-2 infection during the delta (B.1.617.2) variant surge in India: a test-negative, case-control study and a mechanistic study of post-vaccination immune responses. Lancet Infect Dis. 2021 Nov 25:S1473-3099(21)00680-0. doi: 10.1016/S1473-3099(21)00680-0. Epub ahead of print. PMID: 34838183; PMCID: PMC8616567. |
| Thompson, 2021 | Thompson MG, Burgess JL, Naleway AL, Tyner HL, Yoon SK, Meece J, Olsho LEW, Caban-Martinez AJ, Fowlkes A, Lutrick K, Kuntz JL, Dunnigan K, Odean MJ, Hegmann KT, Stefanski E, Edwards LJ, Schaefer-Solle N, Grant L, Ellingson K, Groom HC, Zunie T, Thiese MS, Ivacic L, Wesley MG, Lamberte JM, Sun X, Smith ME, Phillips AL, Groover KD, Yoo YM, Gerald J, Brown RT, Herring MK, Joseph G, Beitel S, Morrill TC, Mak J, Rivers P, Harris KM, Hunt DR, Arvay ML, Kutty P, Fry AM, Gaglani M. Interim Estimates of Vaccine Effectiveness of BNT162b2 and mRNA-1273 COVID-19 Vaccines in Preventing SARS-CoV-2 Infection Among Health Care Personnel, First Responders, and Other Essential and Frontline Workers - Eight U.S. Locations, December 2020-March 2021. MMWR Morb Mortal Wkly Rep. 2021 Apr 2;70(13):495-500. doi: 10.15585/mmwr.mm7013e3. PMID: 33793460; PMCID: PMC8022879. |
| Thompson, 2021 | Thompson MG, Stenehjem E, Grannis S, Ball SW, Naleway AL, Ong TC, DeSilva MB, Natarajan K, Bozio CH, Lewis N, Dascomb K, Dixon BE, Birch RJ, Irving SA, Rao S, Kharbanda E, Han J, Reynolds S, Goddard K, Grisel N, Fadel WF, Levy ME, Ferdinands J, Fireman B, Arndorfer J, Valvi NR, Rowley EA, Patel P, Zerbo O, Griggs EP, Porter RM, Demarco M, Blanton L, Steffens A, Zhuang Y, Olson N, Barron M, Shifflett P, Schrag SJ, Verani JR, Fry A, Gaglani M, Azziz-Baumgartner E, Klein NP. Effectiveness of Covid-19 Vaccines in Ambulatory and Inpatient Care Settings. N Engl J Med. 2021 Oct 7;385(15):1355-1371. doi: 10.1056/NEJMoa2110362. Epub 2021 Sep 8. PMID: 34496194; PMCID: PMC8451184. |
| Thompson, 2021 | Thompson MG, Burgess JL, Naleway AL, Tyner H, Yoon SK, Meece J, Olsho LEW, Caban-Martinez AJ, Fowlkes AL, Lutrick K, Groom HC, Dunnigan K, Odean MJ, Hegmann K, Stefanski E, Edwards LJ, Schaefer-Solle N, Grant L, Ellingson K, Kuntz JL, Zunie T, Thiese MS, Ivacic L, Wesley MG, Mayo Lamberte J, Sun X, Smith ME, Phillips AL, Groover KD, Yoo YM, Gerald J, Brown RT, Herring MK, Joseph G, Beitel S, Morrill TC, Mak J, Rivers P, Poe BP, Lynch B, Zhou Y, Zhang J, Kelleher A, Li Y, Dickerson M, Hanson E, Guenther K, Tong S, Bateman A, Reisdorf E, Barnes J, Azziz-Baumgartner E, Hunt DR, Arvay ML, Kutty P, Fry AM, Gaglani M. Prevention and Attenuation of Covid-19 with the BNT162b2 and mRNA-1273 Vaccines. N Engl J Med. 2021 Jul 22;385(4):320-329. doi: 10.1056/NEJMoa2107058. Epub 2021 Jun 30. PMID: 34192428; PMCID: PMC8262622. |
| Thompson, 2022 | Thompson MG, Natarajan K, Irving SA, et al. Effectiveness of a Third Dose of mRNA Vaccines Against COVID-19–Associated Emergency Department and Urgent Care Encounters and Hospitalizations Among Adults During Periods of Delta and Omicron Variant Predominance — VISION Network, 10 States, August 2021–January 2022. MMWR Morb Mortal Wkly Rep. ePub: 21 January 2022. DOI: http://dx.doi.org/10.15585/mmwr.mm7104e3 |
| Thompson, 2022 | Thompson MG, Natarajan K, Irving SA, Rowley EA, Griggs EP, Gaglani M, Klein NP, Grannis SJ, DeSilva MB, Stenehjem E, Reese SE, Dickerson M, Naleway AL, Han J, Konatham D, McEvoy C, Rao S, Dixon BE, Dascomb K, Lewis N, Levy ME, Patel P, Liao IC, Kharbanda AB, Barron MA, Fadel WF, Grisel N, Goddard K, Yang DH, Wondimu MH, Murthy K, Valvi NR, Arndorfer J, Fireman B, Dunne MM, Embi P, Azziz-Baumgartner E, Zerbo O, Bozio CH, Reynolds S, Ferdinands J, Williams J, Link-Gelles R, Schrag SJ, Verani JR, Ball S, Ong TC. Effectiveness of a Third Dose of mRNA Vaccines Against COVID-19-Associated Emergency Department and Urgent Care Encounters and Hospitalizations Among Adults During Periods of Delta and Omicron Variant Predominance - VISION Network, 10 States, August 2021-January 2022. MMWR Morb Mortal Wkly Rep. 2022 Jan 21;71(4):139-145. doi: 10.15585/mmwr.mm7104e3. PMID: 35085224. |
| Tseng, 2022 | Tseng HF, Ackerson BK, Luo Y, Sy LS, Talarico CA, Tian Y, Bruxvoort KJ, Tubert JE, Florea A, Ku JH, Lee GS, Choi SK, Takhar HS, Aragones M, Qian L. Effectiveness of mRNA-1273 against SARS-CoV-2 Omicron and Delta variants. Nat Med. 2022 Feb 21. doi: 10.1038/s41591-022-01753-y. Epub ahead of print. PMID: 35189624. |
| Vasileiou, 2021 | Vasileiou E, Simpson CR, Shi T, Kerr S, Agrawal U, Akbari A, Bedston S, Beggs J, Bradley D, Chuter A, de Lusignan S, Docherty AB, Ford D, Hobbs FR, Joy M, Katikireddi SV, Marple J, McCowan C, McGagh D, McMenamin J, Moore E, Murray JL, Pan J, Ritchie L, Shah SA, Stock S, Torabi F, Tsang RS, Wood R, Woolhouse M, Robertson C, Sheikh A. Interim findings from first-dose mass COVID-19 vaccination roll-out and COVID-19 hospital admissions in Scotland: a national prospective cohort study. Lancet. 2021 May 1;397(10285):1646-1657. doi: 10.1016/S0140-6736(21)00677-2. Epub 2021 Apr 23. PMID: 33901420; PMCID: PMC8064669. |
| Veneti, 2022 | Veneti L, Bøås H, Bråthen Kristoffersen A, Stålcrantz J, Bragstad K, Hungnes O, Storm ML, Aasand N, Rø G, Starrfelt J, Seppälä E, Kvåle R, Vold L, Nygård K, Buanes EA, Whittaker R. Reduced risk of hospitalisation among reported COVID-19 cases infected with the SARS-CoV-2 Omicron BA.1 variant compared with the Delta variant, Norway, December 2021 to January 2022. Euro Surveill. 2022 Jan;27(4). doi: 10.2807/1560-7917.ES.2022.27.4.2200077. PMID: 35086614. |
| Victor, 2021 | Victor PJ, Mathews KP, Paul H, Mammen JJ, Murugesan M. Protective Effect of COVID-19 Vaccine Among Health Care Workers During the Second Wave of the Pandemic in India. Mayo Clin Proc. 2021 Sep;96(9):2493-2494. doi: 10.1016/j.mayocp.2021.06.003. Epub 2021 Jun 26. PMID: 34366135; PMCID: PMC8233962. |
| Villela, 2021 | Daniel A.M. Villela, Tatiana Guimarães de Noronha, Leonardo S. Bastos, Antonio G. Pacheco, Oswaldo G Cruz, Luiz Max Carvalho, Claudia Torres Codeço, Marcelo Ferreira da Costa Gomes, Flávio Codeço Coelho, Laís Picinini Freitas, Raquel Martins Lana, Victor Bertollo Gomes Porto, Luiz Antônio Bastos Camacho, Claudio José Struchiner. Effectiveness of Mass Vaccination in Brazil against Severe COVID-19 Cases. medRxiv 2021.09.10.21263084; doi: https://doi.org/10.1101/2021.09.10.21263084 |
| Vokó, 2021 | Vokó Z, Kiss Z, Surján G, Surján O, Barcza Z, Pályi B, Formanek-Balku E, Molnár GA, Herczeg R, Gyenesei A, Miseta A, Kollár L, Wittmann I, Müller C, Kásler M. Nationwide effectiveness of five SARS-CoV-2 vaccines in Hungary-the HUN-VE study. Clin Microbiol Infect. 2021 Nov 25:S1198-743X(21)00639-X. doi: 10.1016/j.cmi.2021.11.011. Epub ahead of print. PMID: 34838783; PMCID: PMC8612758. |
| Waldman, 2021 | Waldman SE, Adams JY, Albertson TE, Juárez MM, Myers SL, Atreja A, Batra S, Foster EE, Huynh CV, Liu AY, Lubarsky DA, Ngo VT, Sandrock CE, Taylor SL, Tompkins AM, Cohen SH. Real-world impact of vaccination on coronavirus disease 2019 (COVID-19) incidence in healthcare personnel at an academic medical center. Infect Control Hosp Epidemiol. 2021 Jul 21:1-7. doi: 10.1017/ice.2021.336. Epub ahead of print. PMID: 34287111; PMCID: PMC8353192. |
| Walsh, 2021 | Walsh J, Skally M, Traynor L, de Barra E, Dhuthaigh AN, Hayes B, Fitzpatrick F. Impact of first dose of BNT162b2 vaccine on COVID-19 infection among healthcare workers in an Irish hospital. Ir J Med Sci. 2021 May 27:1–2. doi: 10.1007/s11845-021-02658-4. Epub ahead of print. PMID: 34041693; PMCID: PMC8154332. |
| Whitaker, 2022 | Whitaker HJ, Tsang RS, Byford R, Andrews NJ, Sherlock J, Pillai PS, Williams J, Button E, Campbell H, Sinnathamby M, Victor W, Anand S, Linley E, Hewson J, DArchangelo S, Otter AD, Ellis J, Hobbs RF, Howsam G, Zambon M, Ramsay M, Brown KE, de Lusignan S, Amirthalingam G, Bernal JL. Pfizer-BioNTech and Oxford AstraZeneca COVID-19 vaccine effectiveness and immune response among individuals in clinical risk groups. J Infect. 2022 Jan 3:S0163-4453(21)00664-2. doi: 10.1016/j.jinf.2021.12.044. Epub ahead of print. PMID: 34990709; PMCID: PMC8720678. |
| Wickert, 2021 | Douglas P. Wickert, Erin A. Almand, Kevin J. Baldovich, Christopher A. Cullenbine, Odaro J. Huckstep, Joseph W. Rohrer, John C. Sitko, J. Jordan Steel, Steven C.M. Hasstedt. Estimates of Single Dose and Full Dose BNT162b2 Vaccine Effectiveness among USAF Academy cadets, 1 Mar - 1 May 2021. medRxiv 2021.07.28.21261138; doi: https://doi.org/10.1101/2021.07.28.21261138 |
| Williams, 2021 | Chantal Williams, Dana Al-Bargash, Celeste Macalintal, Rebecca Stuart, Anu Seth, Julienne Latham, Leah Gitterman, Stephanie Fedsin, Marion Godoy, Robert Kozak, Jennifer L Guthrie, Heidi Wood, Allison McGeer, Susy Hota, Elizabeth Rea, Coronavirus Disease 2019 (COVID-19) Outbreak Associated With Severe Acute Respiratory Syndrome Coronavirus 2 (SARS-CoV-2) P.1 Lineage in a Long-Term Care Home After Implementation of a Vaccination Program—Ontario, Canada, April–May 2021, Clinical Infectious Diseases, 2021; ciab617, https://doi.org/10.1093/cid/ciab617 |
| Wu, 2021 | Wu JT, La J, Branch-Elliman W, Huhmann LB, Han SS, Parmigiani G, Tuck DP, Brophy MT, Do NV, Lin AY, Munshi NC, Fillmore NR. Association of COVID-19 Vaccination With SARS-CoV-2 Infection in Patients With Cancer: A US Nationwide Veterans Affairs Study. JAMA Oncol. 2021 Dec 2:e215771. doi: 10.1001/jamaoncol.2021.5771. Epub ahead of print. PMID: 34854921; PMCID: PMC8640949. |
| Yassi, 2021 | Yassi A, Grant JM, Lockhart K, Barker S, Sprague S, Okpani AI, Wong T, Daly P, Henderson W, Lubin S, Kim Sing C. Infection control, occupational and public health measures including mRNA-based vaccination against SARS-CoV-2 infections to protect healthcare workers from variants of concern: A 14-month observational study using surveillance data. PLoS One. 2021 Jul 16;16(7):e0254920. doi: 10.1371/journal.pone.0254920. PMID: 34270608; PMCID: PMC8284646. |
| Young-Xu, 2021 | Young-Xu Y, Korves C, Roberts J, Powell EI, Zwain GM, Smith J, Izurieta HS. Coverage and Estimated Effectiveness of mRNA COVID-19 Vaccines Among US Veterans. JAMA Netw Open. 2021 Oct 1;4(10):e2128391. doi: 10.1001/jamanetworkopen.2021.28391. PMID: 34613401; PMCID: PMC8495523. |
| Young-Xu, 2021 | Young-Xu Y, Zwain GM, Powell EI, Smith J. Estimated Effectiveness of COVID-19 Messenger RNA Vaccination Against SARS-CoV-2 Infection Among Older Male Veterans Health Administration Enrollees, January to September 2021. JAMA Netw Open. 2021 Dec 1;4(12):e2138975. doi: 10.1001/jamanetworkopen.2021.38975. PMID: 34910155. |
| Young-Xu, 2022 | Yinong Young-Xu. Effectiveness of mRNA COVID-19 Vaccines against Omicron among Veterans. medRxiv 2022.01.15.22269360; doi: https://doi.org/10.1101/2022.01.15.22269360 |
| Zacay, 2021 | Zacay G, Shasha D, Bareket R, Kadim I, Hershkowitz Sikron F, Tsamir J, Mossinson D, Heymann AD. BNT162b2 Vaccine Effectiveness in Preventing Asymptomatic Infection With SARS-CoV-2 Virus: A Nationwide Historical Cohort Study. Open Forum Infect Dis. 2021 Jun 9;8(6):ofab262. doi: 10.1093/ofid/ofab262. PMID: 34189176; PMCID: PMC8231369. |
| Zambrano, 2022 | Zambrano LD, Newhams MM, Olson SM, Halasa NB, Price AM, Boom JA, Sahni LC, Kamidani S, Tarquinio KM, Maddux AB, Heidemann SM, Bhumbra SS, Bline KE, Nofziger RA, Hobbs CV, Bradford TT, Cvijanovich NZ, Irby K, Mack EH, Cullimore ML, Pannaraj PS, Kong M, Walker TC, Gertz SJ, Michelson KN, Cameron MA, Chiotos K, Maamari M, Schuster JE, Orzel AO, Patel MM, Campbell AP, Randolph AG; Overcoming COVID-19 Investigators. Effectiveness of BNT162b2 (Pfizer-BioNTech) mRNA Vaccination Against Multisystem Inflammatory Syndrome in Children Among Persons Aged 12-18 Years - United States, July-December 2021. MMWR Morb Mortal Wkly Rep. 2022 Jan 14;71(2):52-58. doi: 10.15585/mmwr.mm7102e1. PMID: 35025852. |
| Zaqout, 2021 | Zaqout A, Daghfal J, Alaqad I, Hussein SAN, Aldushain A, Almaslamani MA, Abukhattab M, Omrani AS. The initial impact of a national BNT162b2 mRNA COVID-19 vaccine rollout. Int J Infect Dis. 2021 Jul;108:116-118. doi: 10.1016/j.ijid.2021.05.021. Epub 2021 May 13. PMID: 33992763; PMCID: PMC8117665. |
