# Supplementary material for: Risk factors affecting COVID-19 vaccine effectiveness identified from 290 cross-country observational studies until February 2022: a meta-analysis and meta-regression
Source: BMC Med. 2022 Nov 25;20:461. doi: 10.1186/s12916-022-02663-z (PMC9701077; doi:10.1186/s12916-022-02663-z)
Supplement: Supplementary file 5 — Additional file 5. Descriptive review of eligible studies, including RoB (NOS stars) (Table S6). [file 12916_2022_2663_MOESM5_ESM.docx]

**Additional file 5**

**Table S6. Descriptive review of eligible studies, including RoB (NOS stars)**

| **Study (Name of the 1^st^ author, year of publication)** | **Country** | **Type of study** | **Start study date** | **End study date** | **Total participants** | **Men (%)** | **Minimum age (years)** | **Mean/Median age (SD/IQR)** | **Vaccinated** | **Commercial vaccines (proportion %)** | **COVID-19** | **Immunization** | **Confirmation of COVID-19** | **Adjustement/Matching** | **N°of VE records** | **NOS stars** |
| --- | --- | --- | --- | --- | --- | --- | --- | --- | --- | --- | --- | --- | --- | --- | --- | --- |
| Abhilash, 2022 | India | Cohort | 1/4/2021 | 31/5/2021 | 4 183 | 57 | 18 | 46 (16) | 949 | ChAdOx1 (79%), BBV152 (21%) | 3,4 | 1,2 | PCR+ | Age, other | 4 | 6 |
| Abu-Raddad, 2022 | Qatar | Case-control | 1/1/2021 | 5/12/2021 | 257 191 | 69 | 18 | 30 (n.r.) | 23 710 | mRNA-1273 (100%) | 1,2,3,4 | 1,2 | PCR+ | Sex, age, other | 70 | 9 |
| Abu-Raddad, 2021 | Qatar | Cohort | 23/2/2021 | 18/3/2021 | 71 958 | 71 | 18 | 33 (15) | 40 390 | BNT162b2 (100%) | 1,3,4 | 1,2 | PCR+ | Sex, age, other | 8 | 7 |
| Accorsi, 2022 | USA | Case-control | 10/12/2021 | 1/1/2022 | 70 155 | 40 | 18 | 40 (16) | 52 978 | BNT162b2 (61%), mRNA-1273 (36%), mRNA (3%) | 2,4 | 3 | PCR+ | Sex, age, other | 4 | 7 |
| Alali, 2021 | Kuwait | Cohort | 24/12/2020 | 15/6/2021 | 3 246 | 82 | 20 | 38 (11) | 2 666 | BNT162b2 (39%), ChAdOx1 (61%) | 2,4 | 1,2 | PCR+ | Sex, age, other | 3 | 8 |
| Alencar, 2021 | Brazil | Cohort | 17/1/2021 | 11/5/2021 | 354 269 | n.r. | 75 | n.r. | 313 328 | ChAdOx1 (44%), CoronaVac (56%) | 4 | 1,2 | n.r. | None | 4 | 4 |
| Amirthalinga, 2021 | UK | Case-control | 26/10/2020 | 18/6/2021 | 526 541 | n.r. | 50 | n.r. | 217 777 | BNT162b2 (35%), ChAdOx1 (65%) | 1,4 | 1,2 | PCR+ | Sex, age, other | 18 | 7 |
| Amit, 2021 | Israel | Cohort | 19/12/2020 | 24/1/2021 | 9 109 | n.r. | 18 | n.r. | 7 214 | BNT162b2 (100%) | 1,2,4 | 1 | PCR+ | Other | 2 | 8 |
| Andeweg, 2022 | Netherlands | Case-control | 22/11/2021 | 19/1/2022 | 528 488 | 47 | 18 | 44 (n.r.) | 342 940 | BNT162b2 (74%), mRNA-1273 (14%), ChAdOx1 (6%), Ad26.COV2.S (6%) | 1,4 | 2,3 | PCR+ | Sex, age, other | 28 | 7 |
| Andrejko, 2021 | USA | Case-control | 24/2/2021 | 29/4/2021 | 1 023 | 51 | 18 | 39 (n.r.) | 256 | BNT162b2, mRNA-1273 | 1,2,4 | 1,2 | PCR+ | Sex, age, other | 5 | 7 |
| Andrews, 2022 | UK | Case-control | 8/12/2020 | 1/10/2021 | 6 056 673 | 37 | 16 | n.r. | 5 200 930 | ChAdOx1 (47%), BNT162b2 (49%), mRNA-1273 (4%) | 2,3,4 | 2 | PCR+ | Sex, age, other | 109 | 9 |
| Andrews, 2022 | UK | Case-control | 13/9/2021 | 5/12/2021 | 893 845 | n.r. | 18 | n.r. | 394 277 | BNT162b2 (43%), mRNA-1273, ChAdOx1 (57%) | 2,3,4 | 2,3 | PCR+ | Sex, age, other | 49 | 8 |
| Andrews, 2022 | UK | Case-control | 27/11/2021 | 12/1/2022 | 2 663 549 | 41 | 18 | 41 (n.r.) | 2 418 833 | ChAdOx1, BNT162b2, mRNA-1273 | 2,4 | 1,2,3 | PCR+ | Sex, age, other | 84 | 8 |
| Angel, 2021 | Israel | Cohort | 20/12/2020 | 25/2/2021 | 6 710 | 33 | 18 | 44 (13) | 5 953 | BNT162b2 (100%) | 2,4 | 1,2 | PCR+ | Sex, age, other | 7 | 9 |
| Aslam, 2022 | USA | Cohort | 1/1/2021 | 12/8/2021 | 1 904 | 64 | 18 | 57 (14) | 1 362 | BNT162b2 (39%), mRNA-1273 (57%), Ad26.COV2.S (4%) | 2,4 | 2 | PCR+ | Sex, age, other | 1 | 7 |
| Aslam, 2021 | USA | Cohort | 1/1/2021 | 6/2/2021 | 2 151 | 65 | 18 | 57 (14) | 912 | BNT162b2 (41%), mRNA-1273 (69%), Ad26.COV2.S (2%) | 2,4 | 2 | PCR+ | None | 1 | 6 |
| Auvigne, 2022 | France | Cohort | 6/12/2021 | 7/1/2022 | 149 064 | 46 | 18 | 41 (n.r.) | 111 626 | BNT162b2 (84%), mRNA-1273 (9%), ChAdOx1 (5%), Ad26.COV2.S (1%) | 3,4 | 2,3 | PCR+ | Sex, age, other | 4 | 7 |
| Azamgarhi, 2021 | UK | Cohort | 5/1/2021 | 26/2/2021 | 2 235 | 32 | 16 | n.r. | 1 409 | BNT162b2 (100%) | 1,4 | 1 | PCR+ | Sex, age, other | 1 | 8 |
| Bajema, 2021 | USA | Case-control | 1/2/2021 | 30/9/2021 | 1 896 | 93 | 18 | 67 (16) | 799 | BNT162b2 (65%), mRNA-1273 (35%) | 3,4 | 2 | PCR+ | Sex, age, other | 14 | 8 |
| Bajema, 2021 | USA | Case-control | 1/2/2021 | 6/8/2021 | 1 175 | 93 | 18 | 68 (16) | 432 | BNT162b2 (66%), mRNA-1273 (34%) | 3,4 | 2 | PCR+ | Sex, age, other | 9 | 6 |
| Baltas, 2021 | UK | Cohort | 30/9/2020 | 15/3/2021 | 595 | 57 | 18 | 79 (20) | 119 | BNT162b2 (66%), ChAdOx1 (34%) | 4 | 1 | PCR+ | Other | 2 | 7 |
| Barchuk, 2021 | Russia | Case-control | 3/7/2021 | 9/8/2021 | 13 893 | 38 | 18 | 48 (16) | 1 964 | Gam-COVID- Vac (100%) | 3,4 | 1,2 | PCR+ | Sex, age, other | 8 | 5 |
| Barchuk, 2022 | Russia | Case-control | 6/10/2021 | 14/10/2021 | 3 945 | 43 | 18 | 47 (15) | 1 679 | Gam-COVID- Vac (88%), Covivac (2%), EpiVacCorona (7%), AdV+InV (3%) | 2,4 | 1,2 | PCR+ | Sex, age, other | 4 | 6 |
| Barlow, 2021 | USA | Case-control | 1/7/2021 | 31/7/2021 | 1 000 | 46 | 16 | 37 (24) | 576 | BNT162b2 (70%), mRNA-1273 (23%), Ad26.COV2.S (7%) | 1,4 | 2 | PCR+/Ag+ | Sex, age, other | 2 | 7 |
| Baum, 2021 | Finland | Cohort | 27/12/2020 | 24/5/2021 | 1 675 618 | 47 | 16 | n.r. | 1 336 395 | mRNA (83%), ChAdOx1 (17%) | 1,3,4 | 1,2 | PCR+/Ag+ | Sex, age, other | 27 | 7 |
| Bedston, 2022 | UK | Cohort | 7/12/2020 | 30/9/2021 | 82 959 | 22 | 16 | n.r. | 46 015 | BNT162b2 (100%) | 1,4 | 1,2 | PCR+ | Sex, age, other | 64 | 9 |
| Bell, 2022 | UK | Cohort | 1/1/2021 | 19/10/2021 | 5 387 | 61 | 18 | 60 (20) | 5 281 | ChAdOx1 (67%), mRNA (33%) | 1,3,4 | 2 | PCR+ | None | 4 | 5 |
| Berec, 2021 | Czechia | Cohort | 26/12/2020 | 20/11/2021 | 7 429 084 | n.r. | 12 | n.r. | 6 287 356 | BNT162b2 (80%), mRNA-1273 (7%), ChAdOx1 (7%), Ad26.COV2.S (6%) | 1,3,4 | 1,2,3 | PCR+ | Sex, age, other | 62 | 8 |
| Bermingham, 2021 | UK | Cohort | 8/12/2020 | 3/5/2021 | 3 422 644 | 46 | 80 | n.r. | 3 121 597 | BNT162b2 (59%), ChAdOx1 (41%) | 4 | 1 | n.r. | age, other | 1 | 5 |
| Bertollini, 2021 | Qatar | Cohort | 18/2/2021 | 26/4/2021 | 261 849 | 75 | 18 | 33 (14) | 311 90 | BNT162b2 (100%), mRNA-1273 | 1,4 | 2 | PCR+ | Sex, age, other | 1 | 7 |
| Bianchi, 2021 | Italy | Cohort | 27/12/2020 | 31/3/2021 | 2 034 | 42 | n.r. | 44 (13) | 1 607 | BNT162b2 (100%) | 1,4 | 1,2 | PCR+ | None | 2 | 6 |
| Bianchi, 2021 | Italy | Cohort | 27/12/2020 | 31/3/2021 | 6 136 | 40 | 18 | 45 (13) | 5 351 | BNT162b2 (100%) | 1,2,4 | 1,2 | PCR+ | None | 8 | 6 |
| Björk, 2021 | Sweden | Cohort | 27/12/2020 | 28/2/2021 | 805 741 | 47 | 18 | 40 (n.r.) | 26 587 | BNT162b2 (100%) | 1,4 | 1,2 | PCR+/Ag+ | Sex, age, other | 6 | 6 |
| Björk, 2021 | Sweden | Case-control | 27/12/2020 | 2/11/2021 | 506 528 | 50 | 0 | 36 (n.r.) | 49 099 | BNT162b2 (85%), mRNA-1273 (8%), ChAdOx1 (7%) | 1,3,4 | 2 | PCR+ | Sex, age, other | 12 | 7 |
| Bobdey, 2021 | India | Cohort | 1/2/2021 | 25/4/2021 | 3 196 | n.r. | 18 | n.r. | 3 102 | ChAdOx1 (100%) | 1,4 | 1,2 | PCR+ | None | 2 | 5 |
| Bouton, 2021 | USA | Cohort | 9/12/2020 | 23/2/2021 | 425 | 18 | 18 | 40 (13) | 96 | BNT162b2 (54%), mRNA-1273 (46%) | 1,4 | 1 | PCR+ | Sex, age, other | 1 | 6 |
| Braeye, 2021 | Belgium | Cohort | 25/1/2021 | 24/6/2021 | 131 283 | 48 | 18 | 33 (19) | 4503 | BNT162b2, mRNA-1273, Ad26.COV2.S, ChAdOx1 | 1,4 | 2 | PCR+ | None | 4 | 5 |
| Britton, 2021 | USA | Cohort | 21/12/2020 | 12/2/2021 | 463 | 37 | 65 | n.r. | 376 | BNT162b2 (100%) | 1,4 | 1 | PCR+ | None | 1 | 5 |
| Britton, 2022 | USA | Case-control | 13/3/2021 | 17/10/2021 | 1 814 383 | 41 | 12 | n.r. | 967 490 | BNT162b2 (58%), mRNA-1273 (34%), Ad26.COV2.S (8%) | 2,4 | 2 | PCR+ | Sex, age, other | 330 | 8 |
| Bruxvoort, 2021 | USA | Case-control | 1/3/2021 | 27/7/2021 | 12 162 | 44 | 18 | 40 (15) | 4 820 | mRNA-1273 (100%) | 1,4 | 1,2 | PCR+ | Sex, age, other | 21 | 7 |
| Bruxvoort, 2022 | USA | Cohort | 1/3/2021 | 30/6/2021 | 705 756 | 41 | 18 | 65 (28) | 352 878 | mRNA-1273 (100%) | 1,2,3,4 | 2 | PCR+ | Sex, age, other | 11 | 8 |
| Buchan, 2022 | Canada | Case-control | 6/12/2021 | 26/12/2021 | 134 435 | 42 | 18 | 41 (16) | 105 118 | BNT162b2 (15%), mRNA-1273 (3%), mRNA (81%) | 2,3,4 | 2,3 | PCR+ | Sex, age, other | 28 | 6 |
| Busic, 2022 | Croatia | Cohort | 1/1/2021 | 30/6/2021 | 218 | 54 | n.r. | 82 (13) | 109 | BNT162b2 (47%), mRNA-1273 (8%), ChAdOx1 (45%) | 4 | 1 | PCR+/Ag+ | Sex, age, other | 2 | 6 |
| Butt, 2021 | Qatar | Cohort | 23/12/2020 | 28/3/2021 | 912 | 61 | 18 | 45 (24) | 456 | BNT162b2 (100%) | 1,4 | 2 | PCR+ | Sex, age, other | 1 | 8 |
| Butt, 2021 | USA | Cohort | 15/12/2020 | 30/6/2021 | 3 456 | 93 | 18 | 70 (13) | 1 728 | BNT162b2, mRNA-1273 | 3,4 | 2 | PCR+ | Sex, age, other | 1 | 8 |
| Butt, 2021 | USA | Case-control | 15/12/2020 | 4/3/2021 | 108 720 | 84 | 18 | 61 (25) | 27 407 | BNT162b2 (48%), mRNA-1273 (52%) | 1,4 | 2 | PCR+ | Sex, age, other | 7 | 7 |
| Butt, 2021 | Qatar | Cohort | 20/12/2020 | 30/5/2021 | 814 | n.r. | 15 | 32 (7) | 407 | BNT162b2 (73%), mRNA-1273 (27%) | 1,4 | 1,2 | PCR+ | Sex, age, other | 4 | 7 |
| Butt, 2021 | Qatar | Cohort | 22/3/2021 | 7/7/2021 | 902 | 54 | 18 | 34 (27) | 191 | BNT162b2, mRNA-1273 | 1,3,4 | 2 | PCR+ | Sex, age, other | 8 | 7 |
| Butt, 2022 | USA | Case-control | 26/1/2021 | 31/8/2021 | 3 973 | 97 | 18 | 69 (11) | 2 700 | BNT162b2 (57%), mRNA-1273 (43%) | 1,4 | 2 | PCR+ | Sex, age, other | 2 | 8 |
| Cabezas, 2021 | Spain | Cohort | 27/12/2020 | 5/3/2021 | 116 693 | 11 | 18 | 54 (12) | 18 199 | BNT162b2 (100%) | 2,3,4 | 1,2 | PCR+ | Sex, age, other | 10 | 7 |
| Callaghan, 2022 | UK | Cohort | 1/9/2020 | 31/8/2021 | 4 147 | 60 | 16 | n.r. | 1 314 | BNT162b2 (38%), ChAdOx1 (62%) | 1,4 | 2 | PCR+ | Sex, age, other | 4 | 8 |
| Carazo, 2021 | Canada | Case-control | 17/1/2021 | 5/6/2021 | 58 476 | 83 | 18 | 41 (20) | 30 574 | BNT162b2 (91%), mRNA-1273 (6%), ChAdOx1 (3%) | 1,2,3,4 | 1,2 | PCR+ | Sex, age, other | 10 | 7 |
| Cavanaugh, 2021 | USA | Cohort | 10/1/2021 | 31/3/2021 | 199 | n.r. | 18 | n.r. | 136 | BNT162b2 (100%) | 1,2,3,4 | 2 | PCR+ | None | 6 | 4 |
| Cerqueira-Silva, 2021 | Brazil | Cohort | 18/1/2021 | 24/7/2021 | 96 193 523 | 45 | 18 | n.r. | 7 543 4880 | ChAdOx1 (66%), CoronaVac (34%) | 1,3,4 | 1,2 | PCR+/Ag+ | Sex, age, other | 72 | 7 |
| Cocchio, 2022 | Italy | Cohort | 27/12/2020 | 7/9/2021 | n.r. | n.r. | 12 | n.r. | 3432638 | BNT162b2 (71%), mRNA-1273 (12%), ChAdOx1 (14%), Ad26.COV2.S (2%) | 1,4 | 1,2 | PCR+ | Sex, age, other | 4 | 6 |
| Coggiola, 2021 | Italy | Cohort | 1/10/2020 | 30/4/2021 | 13 787 | n.r. | 16 | n.r. | n.r. | BNT162b2 | 1,4 | 2 | PCR+ | other | 1 | 3 |
| Cohn, 2021 | USA | Cohort | 1/2/2021 | 1/10/2021 | 780 225 | 86 | 18 | n.r. | 498 148 | BNT162b2 (47%), mRNA-1273 (46%), Ad26.COV2.S (7%) | 1,4 | 2 | PCR+ | Sex, age, other | 27 | 7 |
| Collie, 2021 | South Africa | Case-control | 1/9/2021 | 7/12/2021 | 206 533 | 48 | 18 | n.r. | 112 807 | BNT162b2 (100%) | 1,4 | 2 | PCR+ | Sex, age, other | 2 | 7 |
| Consonni, 2021 | Italy | Cohort | 27/12/2020 | 10/5/2021 | 3 485 | n.r. | 18 | n.r. | 3 152 | BNT162b2 (100%) | 1,4 | 1,2 | PCR+ | Sex, age, other | 2 | 8 |
| Consonni, 2022 | Italy | Cohort | 27/12/2020 | 27/9/2021 | 3 809 | n.r. | 18 | n.r. | 3 678 | BNT162b2 (100%) | 1,2,4 | 1,2 | n.r. | Sex, age, other | 3 | 6 |
| Corchado-Garcia, 2021 | USA | Cohort | 27/2/2021 | 22/7/2021 | 97 787 | 50 | 18 | 52 (17) | 8 889 | Ad26.COV2.S (100%) | 1,4 | 2 | PCR+ | Sex, age, other | 1 | 9 |
| Dagan, 2021 | Israel | Cohort | 20/12/2020 | 1/2/2021 | 1 193 236 | 50 | 16 | 45 (27) | 596 618 | BNT162b2 (100%) | 1,2,3,4 | 1,2 | PCR+ | Sex, age, other | 43 | 9 |
| Dagan, 2021 | Israel | Cohort | 20/12/2020 | 3/6/2021 | 21 722 | n.r. | 16 | n.r. | 10 861 | BNT162b2 (100%) | 1,2,3,4 | 1,2 | PCR+ | Sex, age, other | 5 | 9 |
| de Gier, 2021 | Netherlands | Cohort | 4/4/2021 | 29/8/2021 | 15 571 | n.r. | 15 | n.r. | 1 997 | BNT162b2, mRNA-1273, ChAdOx1, Ad26.COV2.S | 3,4 | 1,2 | PCR+ | Age, other | 27 | 6 |
| Del Cura-Bilbao, 2022 | Spain | Cohort | 28/12/2020 | 31/5/2021 | 925 915 | 52 | n.r. | n.r. | 372 156 | BNT162b2 (65%), mRNA-1273 (9%), ChAdOx1 (26%) | 1,4 | 1,2 | PCR+/Ag+ | Sex, age, other | 5 | 7 |
| Desai, 2021 | India | Cohort | 1/4/2021 | 30/6/2021 | 569 | 61 | 18 | 56 (16) | 137 | ChAdOx1 (100%) | 4 | 1 | PCR+ | Sex, age, other | 1 | 5 |
| Domi, 2021 | USA | Cohort | 18/12/2020 | 7/2/2021 | n.r. | n.r. | n.r. | n.r. | n.r. | BNT162b2 | 1,4 | 1 | PCR+/Ag+ | Other | 6 | 4 |
| Dorabawila, 2022 | USA | Cohort | 13/12/2021 | 30/1/2022 | 5 204 641 | n.r. | 5 | 13 (n.r.) | 1 217 886 | BNT162b2 (100%) | 1,3,4 | 2 | PCR+/Ag+ | None | 12 | 5 |
| Drawz, 2022 | USA | Cohort | 29/8/2021 | 27/11/2021 | 471 835 | 66 | 19 | n.r. | 220 342 | BNT162b2 (59%), mRNA-1273 (41%) | 1,3,4 | 2,3 | PCR+ | None | 87 | 5 |
| Embi, 2021 | USA | Cohort | 17/1/2021 | 5/9/2021 | 89 217 | 45 | 18 | 68 (25) | 40 020 | BNT162b2 (58%), mRNA-1273 (42%) | 3,4 | 2 | PCR+ | Sex, age, other | 18 | 7 |
| Emborg, 2021 | Denmark | Cohort | 27/12/2020 | 11/4/2021 | 864 096 | n.r. | 18 | 62 (18) | 293 191 | BNT162b2 (100%) | 1,3,4 | 1,2 | PCR+ | Sex, age, other | 18 | 8 |
| Fabiani, 2021 | Italy | Cohort | 27/12/2020 | 24/3/2021 | 6 423 | 22 | 18 | 47 (11) | 5 333 | BNT162b2 (100%) | 1,2,4 | 1,2 | PCR+ | Sex, age, other | 4 | 8 |
| Fabiani, 2022 | Italy | Cohort | 27/12/2020 | 7/11/2021 | 33 250 344 | 49 | 16 | n.r. | 3 325 0344 | BNT162b2 (85%), mRNA-1273 (15%) | 1,3,4 | 1,2 | PCR+/Ag+ | Sex, age, other | 77 | 6 |
| Farah, 2022 | Lebanon | Case-control | 1/4/2021 | 31/5/2021 | 1 159 | 45 | 75 | 83 (6) | 103 | BNT162b2 (100%) | 3,4 | 1,2 | PCR+ | Sex, other | 2 | 4 |
| Fisman, 2022 | Canada | Case-control | 30/12/2020 | 30/10/2021 | 264 321 | 52 | n.r. | 48 (n.r.) | 213 195 | mRNA (92%), mRNA+AdV (8%) | 1,4 | 1,2 | PCR+ | Sex, age, other | 14 | 6 |
| Flacco, 2021 | Italy | Cohort | 2/1/2021 | 21/5/2021 | 245 226 | 48 | 18 | 53 (20) | 69 539 | BNT162b2 (69%), mRNA-1273 (7%), ChAdOx1 (24%) | 1,2,4 | 1,2 | PCR+ | Sex, age, other | 6 | 9 |
| Florea, 2021 | USA | Cohort | 18/12/2020 | 30/9/2021 | 1 854 008 | 45 | 18 | 52 (28) | 927 004 | mRNA-1273 (100%) | 1,3,4 | 2 | PCR+ | Sex, age, other | 34 | 8 |
| Fournier, 2022 | France | Cohort | 18/1/2021 | 13/8/2021 | 14 960 | 48 | n.r. | 43 (19) | 1 156 | BNT162b2 (67%), mRNA-1273 (5%), Ad26.COV2.S (2%), ChAdOx1 (9%), mRNA+AdV(16%) | 3,4 | 1,2 | PCR+ | Sex, age, other | 4 | 6 |
| Fowlkes, 2021 | USA | Cohort | 14/12/2020 | 14/8/2021 | 4 217 | n.r. | 18 | n.r. | 3 483 | BNT162b2 (65%), mRNA-1273 (33%), Ad26.COV2.S (2%) | 1,4 | 2 | PCR+ | Other | 6 | 6 |
| Gaio, 2022 | Portugal | Cohort | 27/12/2020 | 30/11/2021 | 2 213 | 19 | 18 | 44 (n.r.) | 2 199 | BNT162b2 (80%), ChAdOx1 (20%) | 2,4 | 1,2 | PCR+ | Sex, age, other | 2 | 7 |
| Garvey, 2021 | UK | Cohort | 28/1/2021 | 21/3/2021 | 33 460 | n.r. | n.r. | n.r. | 12 701 | BNT162b2 (100%) | 1,4 | 1 | PCR+ | None | 1 | 4 |
| Ge, 2022 | USA | Cohort | 14/1/2022 | 15/1/2022 | 78 289 | 55 | 18 | 59 (17) | 10 241 | BNT162b2 (67%), mRNA-1273 (27%), Ad26.COV2.S (5%) | 4 | 1,2 | PCR+ | Sex, age, other | 2 | 7 |
| Ghosh, 2021 | India | Cohort | 16/1/2021 | 30/5/2021 | 1 595 630 | 99 | 18 | 28 (6) | 1 523 347 | ChAdOx1 (100%) | 1,4 | 1,2 | PCR+/Ag+ | None | 2 | 4 |
| Giansante, 2021 | Italy | Cohort | 27/12/2020 | 3/4/2021 | 9 839 | 30 | 18 | n.r. | 8 193 | BNT162b2 (96%), mRNA+AdV (4%) | 1,2,4 | 1,2 | PCR+ | Sex, age, other | 4 | 8 |
| Glampson, 2021 | UK | Cohort | 8/12/2020 | 24/2/2021 | 2 183 939 | 51 | 16 | n.r. | 386 653 | BNT162b2 (58%), ChAdOx1 (42%) | 1,4 | 1 | PCR+ | Sex, age, other | 4 | 7 |
| Glatman-Freedman, 2021 | Israel | Cohort | 21/12/2020 | 6/2/2021 | 2 850 871 | n.r. | 16 | n.r. | 304 027 | BNT162b2 (100%) | 1,2,3,4 | 1,2 | PCR+ | Sex, age, other | 56 | 8 |
| Glatman-Freedman, 2021 | Israel | Cohort | 1/7/2021 | 26/8/2021 | n.r. | n.r. | 12 | n.r. | n.r. | BNT162b2 | 1,4 | 2 | PCR+ | Sex, other | 1 | 6 |
| Goldberg, 2021 | Israel | Cohort | 20/12/2020 | 20/3/2021 | 6 352 000 | 49 | 16 | 44 (n.r.) | n.r. | BNT162b2 | 1,3,4 | 1,2 | PCR+ | Sex, age, other | 36 | 8 |
| Goldin, 2022 | Israel | Cohort | 1/12/2020 | 30/5/2021 | 43 561 | n.r. | 65 | 83 (9) | 39 447 | BNT162b2 (100%) | 1,4 | 1,2 | PCR+ | Age, other | 4 | 8 |
| Goldshtein, 2021 | Israel | Cohort | 19/12/2020 | 11/4/2021 | 15 060 | n.r. | 18 | 31 (5) | 7 530 | BNT162b2 (100%) | 1,4 | 1 | PCR+ | Sex, age, other | 2 | 9 |
| Gomes, 2021 | Germany | Cohort | 9/1/2021 | 11/4/2021 | 11 228 | n.r. | 80 | n.r. | 1 146 | BNT162b2 (100%) | 1,3,4 | 2 | PCR+ | Sex, other | 9 | 7 |
| González, 2021 | Argentina | Cohort | 20/12/2020 | 1/5/2021 | 79 365 | 45 | 60 | 71 (5) | 40 539 | Gam-COVID- Vac (100%) | 1,3,4 | 2 | PCR+/Ag+ | Age, other | 30 | 6 |
| Gram, 2021 | Denmark | Cohort | 9/2/2021 | 23/6/2021 | 5 542 079 | 51 | 18 | 45 (22) | 144 360 | ChAdOx1 (100%) | 1,3,4 | 1 | PCR+ | Sex, age, other | 16 | 8 |
| Grannis, 2021 | USA | Cohort | 1/6/2021 | 31/8/2021 | 14 636 | n.r. | 18 | 65 (29) | 7 676 | BNT162b2 (55%), mRNA-1273 (39%), Ad26.COV2.S (6%) | 3,4 | 2 | PCR+ | Age, other | 3 | 6 |
| Grant, 2021 | France | Case-control | 23/5/2021 | 13/8/2021 | 14 204 | 31 | 18 | 38 (n.r.) | n.r. | BNT162b2, mRNA-1273, ChAdOx1 | 2,4 | 1,2 | PCR+ | Sex, age, other | 5 | 6 |
| Gras-Valentí, 2021 | Spain | Case-control | 27/1/2021 | 7/2/2021 | 268 | 22 | 22 | n.r. | 177 | BNT162b2 (100%) | 1,4 | 1 | PCR+ | Sex, age, other | 5 | 7 |
| Gray, 2021 | South Africa | Case-control | 15/11/2021 | 20/12/2021 | 52 468 | 39 | 18 | n.r. | 5 136 | Ad26.COV2.S (100%) | 3,4 | 3 | PCR+ | Sex, age, other | 2 | 8 |
| Grgič, 2022 | Slovenia | Cohort | 1/1/2021 | 30/10/2021 | n.r. | n.r. | 18 | n.r. | n.r. | BNT162b2, mRNA-1273, ChAdOx1, Ad26.COV2.S | 3,4 | 2 | PCR+/Ag+ | None | 14 | 4 |
| Guijarro, 2021 | Spain | Cohort | 21/12/2020 | 24/4/2021 | 2 590 | n.r. | n.r. | n.r. | 2 116 | BNT162b2 (100%) | 1,4 | 1,2 | PCR+ | Other | 2 | 4 |
| Gupta, 2021 | USA | Cohort | 22/12/2020 | 2/2/2021 | 4 028 | 39 | 18 | 48 (12) | 3 367 | mRNA-1273 (100%) | 1,4 | 1 | PCR+ | None | 1 | 4 |
| Haas, 2021 | Israel | Cohort | 24/1/2021 | 3/4/2021 | 6 538 911 | 49 | 16 | 37 (n.r.) | 4 714 932 | BNT162b2 (100%) | 1,2,3,4 | 1,2 | PCR+ | Sex, age, other | 27 | 8 |
| Hall, 2021 | UK | Cohort | 7/12/2020 | 5/2/2021 | 23 324 | 16 | 18 | 46 (18) | 20 641 | BNT162b2 (100%) | 1,4 | 1,2 | PCR+ | Sex, age, other | 7 | 8 |
| Hall, 2022 | UK | Cohort | 7/12/2020 | 21/9/2021 | 35 768 | 16 | 18 | n.r. | 34 877 | BNT162b2 (89%), ChAdOx1 (11%) | 1,4 | 1,2 | PCR+ | Sex, age, other | 24 | 9 |
| Hansen, 2021 | Denmark | Cohort | 20/11/2021 | 12/12/2021 | n.r. | n.r. | 12 | 28 (n.r.) | n.r. | BNT162b2, mRNA-1273 | 1,4 | 2,3 | PCR+ | Sex, age, other | 19 | 7 |
| Heftdal, 2022 | Denmark | Cohort | 27/12/2020 | 14/9/2021 | 1 549 488 | 49 | 16 | 42 (23) | 1 119 574 | BNT162b2 (100%) | 1,4 | 2 | PCR+ | None | 1 | 6 |
| Hitchings, 2021 | Brazil | Case-control | 1/2/2021 | 9/7/2021 | 138 673 | 46 | 60 | 68 (7) | 28 806 | ChAdOx1 (100%) | 2,3,4 | 1,2 | PCR+ | Age, other | 9 | 7 |
| Hyams, 2021 | UK | Case-control | 18/12/2020 | 26/2/2021 | 466 | 50 | 80 | 87 (7) | 170 | BNT162b2 (64%), ChAdOx1 (36%) | 2,4 | 1 | PCR+ | Sex, other | 2 | 7 |
| Chadeau-Hyam, 2021 | UK | Cross-sectional | 19/10/2021 | 5/11/2021 | 4 830 | n.r. | 12 | n.r. | 1 827 | BNT162b2 (100%) | 1,2,4 | 1 | PCR+ | Sex, age, other | 4 | 6 |
| Chadeau-Hyam, 2022 | UK | Cross-sectional | 24/6/2021 | 27/9/2021 | 81 256 | n.r. | 18 | n.r. | 77 266 | ChAdOx1 (73%), BNT162b2 (25%), mRNA-1273 (2%) | 1,2,4 | 2 | PCR+ | Sex, age, other | 5 | 6 |
| Charmet, 2021 | France | Case-control | 17/2/2021 | 3/5/2021 | 44 795 | 32 | 18 | n.r. | 421 | BNT162b2, mRNA-1273 | 1,4 | 2 | PCR+ | Sex, age, other | 6 | 5 |
| Chemaitelly, 2021 | Qatar | Case-control | 1/1/2021 | 5/9/2021 | 231 826 | 69 | 16 | 31 (17) | 115 913 | BNT162b2 (100%) | 1,2,3,4 | 1,2 | PCR+ | Sex, age, other | 74 | 9 |
| Chemaitelly, 2021 | Qatar | Cohort | 1/2/2021 | 10/5/2021 | 138 872 | 70 | 18 | 32 (15) | 2 282 | mRNA-1273 (100%) | 1,3,4 | 1,2 | PCR+ | Sex, age, other | 6 | 7 |
| Chemaitelly, 2021 | Qatar | Cohort | 1/2/2021 | 21/7/2021 | 782 | 80 | 18 | 50 (45) | 601 | BNT162b2 (93%), mRNA-1273 (7%) | 1,3,4 | 2 | PCR+ | Sex, age, other | 6 | 8 |
| Chemaitelly, 2022 | Qatar | Case-control | 23/12/2021 | 2/2/2022 | 84 884 | 47 | n.r. | 14 (27) | 77 681 | BNT162b2 (74%), mRNA-1273 (26%) | 2,3,4 | 1,2,3 | PCR+ | Sex, age, other | 35 | 9 |
| Chia, 2021 | Singapore | Cohort | 1/4/2021 | 14/6/2021 | 201 | 47 | 18 | 45 (27) | 71 | BNT162b2, mRNA-1273 | 3,4 | 2 | PCR+ | Other | 1 | 3 |
| Chico-Sánchez, 2021 | Spain | Case-control | 27/1/2021 | 6/6/2021 | 624 | 24 | 18 | n.r. | 518 | BNT162b2 (100%) | 2,4 | 1,2 | PCR+ | Other | 9 | 4 |
| Chin, 2021 | USA | Cohort | 16/7/2021 | 15/8/2021 | 827 | 100 | 18 | 37 (10) | 468 | mRNA-1273 (100%) | 1,2,4 | 2 | PCR+/Ag+ | Sex, age, other | 4 | 7 |
| Chin, 2022 | USA | Cohort | 22/12/2020 | 1/3/2021 | 60 707 | 96 | 18 | n.r. | 29 947 | BNT162b2, mRNA-1273 | 1,4 | 1,2 | PCR+/Ag+ | Sex, age, other | 14 | 7 |
| Chodick, 2021 | Israel | Cohort | 19/12/2020 | 17/1/2021 | 503 897 | 48 | 16 | 60 (15) | 351 897 | BNT162b2 (100%) | 1,2,4 | 1 | PCR+ | None | 22 | 5 |
| Chodick, 2021 | Israel | Cohort | 19/12/2020 | 3/3/2021 | 1 178 597 | 48 | 16 | 48 (18) | 872 454 | BNT162b2 (100%) | 1,4 | 2 | PCR+ | Sex, age, other | 8 | 7 |
| Chung, 2021 | Canada | Case-control | 14/12/2020 | 19/4/2021 | 324 033 | 43 | 16 | 43 (18) | 21 272 | BNT162b2, mRNA-1273 | 2,3,4 | 1,2 | PCR+ | Sex, age, other | 48 | 8 |
| Chung, 2022 | USA | Case-control | 1/2/2021 | 30/9/2021 | 2 229 | 35 | 12 | n.r. | 1 288 | BNT162b2 (59%), mRNA-1273 (34%), AdV (7%) | 2,4 | 1,2 | PCR+ | Age, other | 7 | 5 |
| Iliaki, 2021 | USA | Cohort | 16/12/2020 | 31/3/2021 | 4 317 | n.r. | 18 | 45 (13) | 3 249 | BNT162b2, mRNA-1273, Ad26.COV2.S | 1,4 | 1 | PCR+ | Sex, age, other | 2 | 7 |
| Ioannou, 2021 | USA | Cohort | 11/12/2020 | 30/6/2021 | 4 199 742 | 93 | 18 | 69 (13) | 2 099 871 | BNT162b2 (44%), mRNA-1273 (56%) | 1,4 | 1,2 | PCR+ | Sex, age, other | 16 | 8 |
| Iskander, 2021 | USA | Cohort | 1/5/2021 | 31/8/2021 | 57 104 | n.r. | 17 | n.r. | 42 008 | BNT162b2 (66%), mRNA-1273 (28%), Ad26.COV2.S (6%) | 1,4 | 2 | n.r. | None | 4 | 2 |
| Jalali, 2022 | Norway | Cohort | 14/12/2021 | 14/1/2022 | 1 526 | 47 | 16 | 42 (n.r.) | 1 367 | BNT162b2, mRNA-1273 | 1,4 | 2,3 | PCR+ | None | 4 | 6 |
| Jameson, 2021 | USA | Cohort | 17/12/2020 | 24/3/2021 | 4 318 | n.r. | 18 | n.r. | 3 023 | BNT162b2 (100%) | 1,4 | 2 | n.r. | None | 1 | 3 |
| John, 2021 | USA | Cohort | 18/12/2020 | 17/3/2021 | 40 074 | 97 | 18 | 69 (16) | 20 037 | BNT162b2 (49%), mRNA-1273 (51%) | 1,3,4 | 1,2 | PCR+ | Sex, age, other | 6 | 7 |
| John, 2022 | USA | Cohort | 1/3/2021 | 1/6/2021 | 762 | 96 | 18 | 64 (n.r.) | 254 | BNT162b2 (50%), mRNA-1273 (48%), mRNA (3%) | 4 | 1,2 | PCR+ | Sex, age, other | 4 | 7 |
| Johnson, 2022 | USA | Cohort | 4/4/2021 | 25/12/2021 | 1 987 683 | n.r. | 18 | n.r. | 925 999 | BNT162b2 (59%), mRNA-1273 (31%), Ad26.COV2.S (9%) | 1,4 | 2,3 | PCR+/Ag+ | None | 30 | 4 |
| June Choe, 2022 | Korea | Cohort | 19/7/2021 | 5/9/2021 | 1 307 663 | n.r. | 16 | n.r. | 444 313 | BNT162b2 (100%) | 1,4 | 1,2 | PCR+ | None | 2 | 5 |
| Kale, 2021 | India | Cohort | 16/1/2021 | 31/5/2021 | 1 858 | n.r. | n.r. | n.r. | 1639 | ChAdOx1 (100%) | 1,3,4 | 1,2 | PCR+ | None | 4 | 4 |
| Katikireddi, 2021 | UK | Cohort | 18/1/2021 | 25/10/2021 | 58 548 165 | 47 | 18 | 49 (21) | 4 503 0653 | ChAdOx1 (100%) | 2,3,4 | 1,2 | PCR+/Ag+ | Sex, age, other | 42 | 7 |
| Katz, 2021 | Israel | Cohort | 27/12/2020 | 15/5/2021 | 1 250 | 20 | 18 | 45 (19) | 998 | BNT162b2 (100%) | 1,2,4 | 2 | PCR+ | Sex, age, other | 2 | 8 |
| Keegan, 2021 | USA | Cohort | 16/1/2021 | 15/10/2021 | 3 205 958 | n.r. | 16 | n.r. | 1 726 946 | BNT162b2 (57%), mRNA-1273 (34%), Ad26.COV2.S (9%) | 1,4 | 2 | PCR+ | None | 4 | 3 |
| Khan, 2021 | USA | Cohort | 18/12/2020 | 20/4/2021 | 14 697 | 92 | 18 | 68 (20) | 7 321 | BNT162b2 (45%), mRNA-1273 (55%) | 1,3,4 | 1,2 | PCR+ | Sex, age, other | 6 | 8 |
| Khan, 2022 | Qatar | Cohort | 1/1/2021 | 31/3/2021 | 952 | n.r. | n.r. | n.r. | 476 | BNT162b2 (100%) | 1,2,4 | 1,2 | n.r. | Age, other | 4 | 4 |
| Kim, 2021 | USA | Case-control | 1/2/2021 | 28/5/2021 | 812 | 32 | 16 | n.r. | 364 | BNT162b2 (62%), mRNA-1273 (38%) | 1,4 | 1,2 | PCR+ | age, other | 2 | 7 |
| Kissling, 2021 | EU | Case-control | 10/12/2020 | 31/5/2021 | 4 964 | 26 | 65 | n.r. | 1 871 | BNT162b2 (74%), ChAdOx1 (26%) | 2,4 | 1,2 | PCR+/Ag+ | Sex, age, other | 3 | 6 |
| Lan, 2021 | USA | Cohort | 16/12/2020 | 30/9/2021 | 4 615 | n.r. | 18 | 45 (13) | 4 418 | BNT162b2 (39%), mRNA-1273 (58%), Ad26.COV2.S (2%) | 1,4 | 1,2 | PCR+ | Sex, age, other | 3 | 8 |
| Larese Filon, 2022 | Italy | Cohort | 1/3/2021 | 31/5/2021 | 4 251 | 31 | 18 | 47 (11) | 3 060 | BNT162b2 (100%) | 1,4 | 2 | PCR+ | Age, other | 1 | 7 |
| Lauring, 2022 | USA | Case-control | 11/3/2021 | 14/1/2022 | 11 690 | 51 | 18 | 62 (22) | 5 365 | BNT162b2 (61%), mRNA-1273 (39%) | 3,4 | 2,3 | PCR+ | Sex, age, other | 20 | 6 |
| Lefèvre, 2021 | France | Cohort | 15/1/2021 | 19/5/2021 | 376 | 24 | 55 | n.r. | 336 | BNT162b2 | 1,3,4 | 1,2 | PCR+ | Sex, age, other | 4 | 7 |
| Lewis, 2021 | USA | Case-control | 11/3/2021 | 15/8/2021 | 3 619 | 52 | 18 | 58 (25) | 1 240 | BNT162b2, mRNA-1273 | 3,4 | 2 | PCR+ | Sex, age, other | 7 | 5 |
| Lin, 2022 | USA | Cohort | 11/12/2020 | 8/9/2021 | 10 600 823 | 51 | 12 | n.r. | 6 007 467 | BNT162b2 (56%), mRNA-1273 (38%), Ad26.COV2.S (6%) | 2,3,4 | 2 | n.r. | Sex, age, other | 270 | 6 |
| Liu, 2021 | USA | Cohort | 18/6/2021 | 21/9/2021 | 114 156 | 38 | 18 | 53 (20) | 14 362 | BNT162b2 (67%), mRNA-1273 (33%) | 3,4 | 2 | PCR+/Ag+ | Age, other | 2 | 6 |
| Lopez Bernal, 2021 | UK | Case-control | 5/4/2021 | 23/5/2021 | 19 109 | 49 | 16 | n.r. | n.r. | BNT162b2, ChAdOx1 | 2,4 | 1,2 | PCR+ | Sex, age, other | 8 | 8 |
| Lopez Bernal, 2021 | UK | Case-control | 8/12/2020 | 21/2/2021 | 156 930 | 45 | 70 | n.r. | 138 869 | BNT162b2 (45%), ChAdOx1 (55%) | 1,4 | 1,2 | PCR+ | Sex, age, other | 9 | 7 |
| Lopez Bernal, 2021 | UK | Cohort | 8/12/2020 | 17/4/2021 | 48 096 | n.r. | 70 | n.r. | 6 596 | ChAdOx1 (41%), BNT162b2 (59%) | 4 | 1,2 | PCR+ | Sex, age, other | 3 | 6 |
| Lumley, 2021 | UK | Cohort | 1/12/2020 | 28/2/2021 | 13 109 | 26 | 18 | 39 (20) | 11 023 | BNT162b2 (75%), ChAdOx1 (25%) | 1,2,4 | 1,2 | PCR+ | Sex, age, other | 3 | 8 |
| Luong, 2021 | France | Case-control | 23/12/2020 | 26/6/2021 | 853 | 55 | 18 | 71 (23) | 170 | BNT162b2 (75%), ChAdOx1 (14%), mRNA+AdV (11%) | 3,4 | 1,2 | PCR+ | Age, other | 2 | 5 |
| Lutrick, 2021 | USA | Cohort | 25/7/2021 | 4/12/2021 | 243 | 51 | 12 | n.r. | 194 | BNT162b2 (100%) | 1,4 | 2 | PCR+ | Sex, age, other | 1 | 8 |
| Lytras, 2022 | Greece | Cohort | 11/1/2021 | 8/12/2021 | 9 200 000 | n.r. | 15 | n.r. | 1 467 6605 | BNT162b2 (78%), mRNA-1273 (8%), ChAdOx1 (10%), Ad26.COV2.S (4%) | 4 | 2,3 | PCR+/Ag+ | Age, other | 29 | 5 |
| Macchia, 2021 | Argentina | Cohort | 29/12/2020 | 15/5/2021 | 663 602 | 50 | 60 | 74 (9) | 540 792 | Gam-COVID- Vac (63%), ChAdOx1 (25%), BBIBP-CorV (12%) | 1,4 | 1,2 | PCR+ | Sex, age, other | 12 | 7 |
| Maeda, 2022 | Japan | Case-control | 1/7/2021 | 30/9/2021 | 1 936 | 53 | 16 | 49 (42) | 816 | BNT162b2 (83%), mRNA-1273 (17%) | 2,4 | 1,2 | PCR+ | Sex, age, other | 8 | 5 |
| Machado, 2021 | Portugal | Cohort | 2/2/2021 | 16/9/2021 | 1 884 934 | n.r. | n.r. | 74 (7) | 1 746 452 | mRNA (68%), ChAdOx1 (29%), Ad26.COV2.S (2%) | 2,3,4 | 2 | PCR+ | Sex, age, other | 37 | 7 |
| Mallow, 2022 | USA | Cohort | 1/1/2021 | 25/8/2021 | 13 203 | 49 | 18 | 61 (26) | 3 242 | BNT162b2 (64%), mRNA-1273 (36%) | 2,4 | 2 | PCR+ | Sex, age, other | 2 | 6 |
| Manley, 2021 | France | Cohort | 1/2/2021 | 2/10/2021 | 11 191 | 81 | 18 | 63 (15) | 11 191 | BNT162b2 (40%), mRNA-1273 (55%), Ad26.COV2.S (5%) | 2,3,4 | 1,2 | PCR+ | Sex, age, other | 16 | 7 |
| Martínez-Baz, 2021 | Spain | Cohort | 1/1/2021 | 30/4/2021 | 20 961 | 51 | 18 | n.r. | 1 325 | BNT162b2 (60%), ChAdOx1 (40%) | 1,2,3,4 | 1,2 | PCR+ | Sex, age, other | 15 | 9 |
| Martínez-Baz, 2021 | Spain | Cohort | 1/4/2021 | 30/8/2021 | 30 240 | 48 | 18 | n.r. | 15 892 | BNT162b2 (64%), mRNA-1273 (10%), ChAdOx1 (20%), Ad26.COV2.S (6%) | 1,2,3,4 | 1,2 | PCR+ | Sex, age, other | 38 | 7 |
| Mason, 2021 | UK | Case-control | 21/12/2020 | 3/2/2021 | 1 663 392 | 48 | 80 | n.r. | 170 226 | BNT162b2 (100%) | 1,3,4 | 1 | PCR+ | Sex, other | 8 | 7 |
| Mateo-Urdiales, 2021 | Italy | Cohort | 27/12/2020 | 18/4/2021 | 14 365 241 | n.r. | 16 | n.r. | 1 372 1506 | BNT162b2 (61%), ChAdOx1 (31%), mRNA-1273 (7%), Ad26.COV2.S (1%) | 1,4 | 2 | PCR+/Ag+ | Other | 5 | 5 |
| Matsuo, 2022 | Japan | Cohort | 1/7/2021 | 14/10/2021 | 2 692 | 56 | 20 | n.r. | 440 | BNT162b2, mRNA-1273 | 3,4 | 1,2 | PCR+/Ag+ | Sex, age, other | 7 | 7 |
| Mattiuzzi, 2022 | Italy | Cohort | 27/12/2020 | 30/12/2021 | 54 000 000 | n.r. | n.r. | n.r. | 45 600 000 | BNT162b2 (72%), mRNA-1273 (13%), ChAdOx1 (13%), Ad26.COV2.S (2%) | 1,3,4 | 2,3 | PCR+ | None | 9 | 5 |
| Mattiuzzi, 2022 | Italy | Cohort | 7/12/2020 | 17/12/2021 | 4 487 526 | n.r. | 80 | n.r. | 4 271 102 | BNT162b2 (70%), mRNA-1273 (17%), ChAdOx1 (12%), Ad26.COV2.S (1%) | 1,3,4 | 2,3 | PCR+ | None | 9 | 6 |
| Mazagatos, 2021 | Spain | Cohort | 27/12/2020 | 4/4/2021 | 338 145 | n.r. | 65 | n.r. | 279 646 | BNT162b2, mRNA-1273 | 1,3,4 | 1,2 | PCR+/Ag+ | other | 6 | 4 |
| McEvoy, 2022 | Canada | Cohort | 7/6/2021 | 19/7/2021 | 1 793 | 64 | 18 | 60 (18) | 1 540 | BNT162b2 (77%), mRNA-1273 (18%), ChAdOx1 (5%) | 3,4 | 1,2 | n.r. | Sex, age, other | 2 | 5 |
| McKeigue, 2021 | UK | Case-control | 1/12/2020 | 19/8/2021 | 223 742 | n.r. | 18 | n.r. | 79 956 | BNT162b2 (35%), mRNA-1273 (3%), ChAdOx1 (62%) | 3,4 | 1,2 | PCR+ | Sex, age, other | 4 | 7 |
| McKeigue, 2021 | UK | Case-control | 1/12/2020 | 8/9/2021 | 55 730 | n.r. | 18 | n.r. | 8 866 | mRNA (31%), ChAdOx1 (69%) | 3,4 | 1,2 | PCR+ | Sex, age, other | 8 | 6 |
| McLean, 2022 | USA | Cohort | 21/6/2021 | 7/12/2021 | 1 266 | 41 | 12 | n.r. | 937 | BNT162b2 (65%), mRNA-1273 (35%) | 1,2,4 | 2 | PCR+ | None | 6 | 5 |
| Mendola, 2021 | Italy | Cohort | 28/12/2020 | 15/5/2021 | 2 478 | 29 | 18 | 45 (12) | 2 037 | BNT162b2 (100%) | 1,4 | 2 | n.r. | Sex, age, other | 3 | 5 |
| Menni, 2021 | UK | Cohort | 4/1/2021 | 10/3/2021 | 587 978 | 37 | 16 | 51 (15) | 103 622 | ChAdOx1 (35%), BNT162b2 (65%) | 1,4 | 1 | PCR+ | Sex, age, other | 5 | 6 |
| Mirahmadizadeh, 2022 | Iran | Cohort | 9/2/2021 | 22/10/2021 | 1 882 148 | 51 | 18 | 47 (18) | 881 638 | BBIBP-CorV (75%), ChAdOx1 (15%), Gam-COVID- Vac (2%), BIV1-Coviran (8%) | 1,3,4 | 2,3 | PCR+ | Other | 42 | 6 |
| Moline, 2021 | USA | Cohort | 1/2/2021 | 30/4/2021 | 6 712 | n.r. | 65 | n.r. | 1 829 | BNT162b2 (65%), mRNA-1273 (33%), Ad26.COV2.S (2%) | 3,4 | 1,2 | PCR+ | Sex, age, other | 10 | 6 |
| Monge, 2021 | Spain | Cohort | 27/12/2020 | 10/3/2021 | 299 209 | 29 | 65 | 86 (8) | 296 093 | BNT162b2 (100%), mRNA-1273 | 1,4 | 1,2 | PCR+/Ag+ | other | 2 | 6 |
| Mor, 2021 | Israel | Cohort | 1/3/2021 | 31/5/2021 | 3 050 | 45 | 16 | 41 (17) | 717 | BNT162b2 (100%) | 1,4 | 2 | PCR+ | Age, other | 1 | 6 |
| Morgan, 2022 | USA | Cohort | 15/6/2021 | 20/8/2021 | 10 092 | n.r. | 18 | 28 (5) | 1 332 | BNT162b2 (66%), mRNA-1273 (29%), Ad26.COV2.S (5%) | 1,3,4 | 2 | n.r. | Sex, age, other | 2 | 5 |
| Moustsen-Helms, 2021 | Denmark | Cohort | 27/12/2020 | 18/2/2021 | 370 079 | 20 | 18 | 51 (20) | 129 037 | BNT162b2 (100%) | 1,4 | 1,2 | PCR+ | Age, other | 4 | 7 |
| Muhsen, 2021 | Israel | Cohort | 1/1/2021 | 11/4/2021 | 9 162 | 20 | 16 | 46 (12) | 6 960 | BNT162b2 (100%) | 1,4 | 2 | PCR+ | Sex, age, other | 1 | 7 |
| Muthukrishnan, 2021 | India | Cross-sectional | 1/3/2021 | 17/5/2021 | 1 168 | 67 | 18 | 55 (18) | 450 | ChAdOx1 (100%) | 4 | 1,2 | PCR+ | Sex, age, other | 2 | 7 |
| Nanduri, 2021 | USA | Cohort | 1/3/2021 | 1/8/2021 | 10 428 783 | n.r. | 65 | n.r. | 8 897 337 | BNT162b2 (58%), mRNA-1273 (30%), Others (12%) | 1,4 | 2 | PCR+ | Other | 6 | 6 |
| Nasreen, 2022 | Canada | Case-control | 11/1/2021 | 3/8/2021 | 682 071 | 45 | 18 | 43 (17) | 168 484 | BNT162b2 (73%), mRNA-1273 (18%), ChAdOx1 (8%) | 2,3,4 | 1,2 | PCR+ | Sex, age, other | 129 | 6 |
| Ng, 2021 | Singapore | Cohort | 1/9/2020 | 31/5/2021 | 1 001 | 56 | 18 | 36 (24) | 230 | BNT162b2 (83%), mRNA-1273 (17%) | 1,2,4 | 1 | PCR+ | Sex, age, other | 2 | 7 |
| Nguyen, 2022 | Portugal | Cohort | 6/12/2021 | 26/12/2021 | 13 143 | 50 | 12 | 38 (n.r.) | 11 940 | BNT162b2 (60%), ChAdOx1 (8%), mRNA+AdV (31%) | 1,4 | 2,3 | PCR+ | Sex, age, other | 8 | 6 |
| Niessen, 2021 | Netherlands | Case-control | 1/5/2021 | 28/6/2021 | 634 | 57 | 18 | 68 (21) | 155 | BNT162b2 (65%), mRNA-1273 (12%), ChAdOx1 (18%), Ad26.COV2.S (6%) | 3,4 | 1,2 | PCR+ | Sex, age, other | 26 | 7 |
| Nordström, 2021 | Sweden | Cohort | 1/1/2021 | 23/8/2021 | 541 071 | n.r. | 16 | n.r. | 360 355 | ChAdOx1, BNT162b2, mRNA-1273 | 2,4 | 2 | n.r. | Sex, age, other | 5 | 7 |
| Nordström, 2022 | Sweden | Cohort | 28/12/2020 | 4/10/2021 | 1 685 948 | 41 | n.r. | 53 (31) | 842 974 | BNT162b2 (76%), mRNA-1273 (9%), ChAdOx1 (9%), mRNA+AdV (6%) | 1,3,4 | 2 | PCR+ | Sex, age, other | 60 | 8 |
| North, 2021 | USA | Cohort | 30/12/2020 | 2/4/2021 | 2 247 | 22 | n.r. | 37 (20) | 2 242 | BNT162b2 (36%), mRNA-1273 (64%) | 1,4 | 1,2 | PCR+ | None | 2 | 4 |
| Nunes, 2021 | Portugal | Cohort | 2/2/2021 | 13/8/2021 | 1 339 309 | 43 | 65 | n.r. | 1 187 029 | BNT162b2 (86%), mRNA-1273 (14%) | 3,4 | 1,2 | PCR+ | Sex, age, other | 16 | 8 |
| Nunes, 2022 | South Africa | Case-control | 24/11/2021 | 31/12/2021 | 433 | 18 | 18 | 38 (10) | 345 | Ad26.COV2.S (92%), BNT162b2 (8%) | 2,4 | 2,3 | PCR+ | Other | 3 | 5 |
| Oliveira, 2022 | USA | Case-control | 1/6/2021 | 15/8/2021 | 542 | 52 | 12 | 14 (3) | 154 | BNT162b2 (100%) | 1,2,4 | 1,2 | PCR+ | Age, other | 7 | 7 |
| Olson, 2021 | USA | Case-control | 1/6/2021 | 30/9/2021 | 464 | 55 | 12 | 15 (3) | 99 | BNT162b2 (100%) | 3,4 | 2 | PCR+/Ag+ | Sex, age, other | 3 | 6 |
| Olson, 2022 | USA | Case-control | 1/7/2021 | 25/10/2021 | 1 222 | 50 | 12 | 15 (3) | 299 | BNT162b2 (100%) | 3,4 | 1,2 | PCR+/Ag+ | Sex, age, other | 4 | 8 |
| Ostropolets, 2021 | USA | Cohort | 1/12/2020 | 30/6/2021 | 313 314 | 37 | 10 | n.r. | 156 657 | BNT162b2 (65%), mRNA-1273 (32%), Ad26.COV2.S (3%) | 1,3,4 | 2 | PCR+ | Sex, age, other | 6 | 8 |
| Paris, 2021 | France | Cohort | 4/1/2021 | 17/5/2021 | 8 165 | n.r. | 18 | 40 (52) | 4 592 | BNT162b2 (36%), mRNA-1273 (29%), ChAdOx1 (35%) | 1,4 | 1,2 | PCR+ | Age, other | 4 | 7 |
| Pascucci, 2021 | Italy | Cohort | 28/12/2020 | 31/3/2021 | 6 570 | 39 | 20 | 39 (56) | 5 152 | BNT162b2 (100%) | 1,4 | 2 | PCR+ | None | 1 | 6 |
| Pawlowski, 2021 | USA | Cohort | 1/12/2020 | 20/4/2021 | 136 532 | 42 | 18 | 56 (18) | 68 266 | BNT162b2 (76%), mRNA-1273 (24%) | 1,3,4 | 1,2 | PCR+ | Sex, age, other | 6 | 8 |
| Petráš, 2021 | Czechia | Cohort | 27/12/2020 | 31/8/2021 | 11 443 | n.r. | 18 | 42 (14) | 6 369 | BNT162b2 (100%) | 1,4 | 1,2 | PCR+ | Sex, age, other | 14 | 9 |
| Pilishvili, 2021 | USA | Case-control | 1/1/2021 | 19/5/2021 | 4 931 | 17 | 18 | 37 (57) | 2 242 | BNT162b2 (83%), mRNA-1273 (17%) | 2,4 | 1,2 | PCR+/Ag+ | Age, other | 17 | 6 |
| Pilishvili, 2021 | USA | Case-control | 1/1/2021 | 31/3/2021 | 1 843 | 17 | 19 | 37 (55) | 1 194 | BNT162b2 (78%), mRNA-1273 (22%) | 1,4 | 1,2 | PCR+/Ag+ | Age, other | 2 | 6 |
| Polinski, 2021 | USA | Cohort | 1/3/2021 | 31/7/2021 | 1 914 670 | 44 | 18 | 55 (17) | 390 517 | Ad26.COV2.S (100%) | 1,3,4 | 2 | PCR+ | Sex, age, other | 29 | 7 |
| Porru, 2022 | Italy | Cohort | 28/2/2021 | 3/5/2021 | 9 811 | 31 | 18 | 36 (23) | 7 592 | BNT162b2 (100%) | 1,4 | 2 | PCR+ | Sex, age, other | 1 | 7 |
| Poukka, 2022 | Finland | Cohort | 27/12/2020 | 26/10/2021 | 427 905 | 14 | 17 | n.r. | 384 456 | BNT162b2, mRNA-1273, ChAdOx1 | 1,3,4 | 1,2 | PCR+ | Sex, age, other | 32 | 8 |
| Pouwels, 2021 | UK | Case-control | 1/12/2020 | 2/8/2021 | 3 391 645 | 46 | 18 | 56 (27) | n.r. | BNT162b2, ChAdOx1 | 1,2,4 | 1,2 | PCR+ | None | 40 | 5 |
| Powell, 2022 | UK | Case-control | 29/11/2021 | 12/1/2022 | 842 969 | 49 | 12 | 14 (n.r.) | 280 009 | BNT162b2 (100%) | 1,4 | 1,2 | PCR+ | Sex, age, other | 44 | 8 |
| Pramod, 2022 | India | Case-control | 1/3/2021 | 30/5/2021 | 720 | 50 | 18 | 34 (15) | 515 | ChAdOx1 (100%) | 1,2,4 | 1,2 | PCR+ | Sex, age, other | 4 | 8 |
| Pritchard, 2021 | UK | Cohort | 1/12/2020 | 8/5/2021 | 383 812 | 46 | 18 | 55 (28) | 324 694 | ChAdOx1 (57%), BNT162b2 (43%) | 1,2,4 | 1,2 | PCR+ | Sex, age, other | 12 | 8 |
| Prunas, 2022 | Israel | Case-control | 15/6/2021 | 8/12/2021 | 238 023 | 48 | 12 | 14 (1.4) | n.r. | BNT162b2 | 1,2,4 | 1,2 | PCR+ | Sex, age, other | 8 | 8 |
| Puranik, 2022 | USA | Case-control | 1/12/2020 | 22/9/2021 | 21 863 | 39 | 18 | 53 (18) | 21 863 | BNT162b2 (69%), mRNA-1273 (31%) | 2,4 | 2 | PCR+ | Sex, age, other | 6 | 8 |
| Rane, 2022 | USA | Case-control | 1/4/2021 | 25/10/2021 | 1 058 493 | 43 | 12 | 33 (24) | 592 041 | BNT162b2, mRNA-1273, Ad26.COV2.S | 1,2,4 | 1,2 | PCR+/Ag+ | Sex, age, other | 45 | 6 |
| Ranzani, 2021 | Brazil | Case-control | 25/6/2021 | 30/9/2021 | 11 817 | 48 | 18 | 37 (17) | 1 162 | Ad26.COV2.S (100%) | 2,3,4 | 2 | PCR+ | Sex, age, other | 6 | 7 |
| Ranzani, 2022 | Brazil | Case-control | 17/1/2021 | 27/11/2021 | 10 077 | 37 | 18 | 40 (14) | 2 393 | ChAdOx1 (100%) | 1,2,4 | 1,2 | PCR+ | Sex, age, other | 20 | 8 |
| Regev-Yochay, 2021 | Israel | Cohort | 19/12/2020 | 14/3/2021 | 4 373 | 27 | 18 | n.r. | 2 932 | BNT162b2 (100%) | 1,2,4 | 1,2 | PCR+ | Other | 4 | 8 |
| Reis, 2021 | Israel | Cohort | 8/6/2021 | 14/9/2021 | 188 708 | 51 | 12 | n.r. | 94 354 | BNT162b2 (100%) | 1,2,4 | 1,2 | PCR+ | Sex, age, other | 4 | 8 |
| Reynolds, 2022 | USA | Case-control | 1/3/2021 | 16/9/2021 | 2 481 | 15 | 18 | 46 (15) | 1 565 | BNT162b2 (52%), mRNA-1273 (41%), Ad26.COV2.S (7%) | 1,2,4 | 1,2 | PCR+ | Sex, age, other | 4 | 6 |
| Risk, 2022 | USA | Cohort | 1/4/2021 | 20/10/2021 | 159 055 | 42 | 18 | 49 (30) | 113 936 | BNT162b2 (65%), mRNA-1273 (30%), Ad26.COV2.S (5%) | 1,3,4 | 2 | PCR+ | Sex, age, other | 20 | 9 |
| Roberts, 2022 | USA | Cohort | 1/1/2021 | 31/12/2021 | 170 487 | n.r. | 18 | 51 (19) | 74 060 | BNT162b2, mRNA-1273, Ad26.COV2.S | 1,4 | 2 | PCR+ | Sex, age, other | 14 | 8 |
| Robilotti, 2021 | USA | Cohort | 29/1/2021 | 1/9/2021 | 13 658 | n.r. | 18 | n.r. | n.r. | BNT162b2, mRNA-1273 | 2,4 | 1,2 | PCR+ | None | 4 | 4 |
| Robles-Fontán, 2022 | USA | Cohort | 15/12/2020 | 15/10/2021 | 3 285 874 | n.r. | 18 | n.r. | 2 276 966 | mRNA-1273 (38%), BNT162b2 (56%), Ad26.COV2.S (6%) | 1,3,4 | 2 | PCR+ | Sex, age, other | 18 | 5 |
| Rosenberg, 2021 | USA | Cohort | 1/5/2021 | 3/9/2021 | 8 690 825 | n.r. | 18 | n.r. | 5 638 142 | BNT162b2 (48%), mRNA-1273 (42%), Ad26.COV2.S (10%) | 1,3,4 | 2 | PCR+ | Age, other | 63 | 5 |
| Rovida, 2021 | Italy | Cohort | 18/1/2021 | 19/4/2021 | 4 066 | n.r. | 16 | n.r. | 3 720 | BNT162b2 (100%) | 1,4 | 2 | PCR+ | None | 1 | 5 |
| Rudolph, 2021 | USA | Cohort | 20/12/2020 | 14/2/2021 | 5 074 | n.r. | 18 | 41 (48) | 4 364 | BNT162b2 (100%) | 1,4 | 1 | n.r. | None | 1 | 3 |
| Saciuk, 2021 | Israel | Cohort | 18/1/2021 | 25/4/2021 | 1 650 885 | 48 | 16 | n.r. | 1 347 976 | BNT162b2 (100%) | 1,3,4 | 2 | PCR+ | Sex, age, other | 27 | 9 |
| Sagiraju, 2021 | India | Cohort | 1/4/2021 | 30/6/2021 | 1 748 | 66 | 18 | 50 (n.r.) | 318 | BBV152 (50%), ChAdOx1 (50%) | 4 | 1,2 | n.r. | Sex, age, other | 2 | 5 |
| Satwik, 2021 | India | Cohort | 1/3/2021 | 31/5/2021 | 4 296 | n.r. | 18 | n.r. | 3 339 | ChAdOx1 (100%) | 2,3,4 | 1,2 | PCR+ | Sex, age, other | 6 | 8 |
| Self, 2021 | USA | Case-control | 11/3/2021 | 15/8/2021 | 3 689 | 52 | 18 | 58 (25) | 1 327 | BNT162b2 (56%), mRNA-1273 (36%), Ad26.COV2.S (9%) | 3,4 | 2 | PCR+/Ag+ | Sex, age, other | 5 | 7 |
| Seppälä, 2021 | Norway | Cohort | 15/4/2021 | 15/8/2021 | 4 204 859 | 50 | 18 | n.r. | 3 295 684 | BNT162b2 (81%), mRNA-1273 (12%), ChAdOx1 (1%), mRNA+AdV (6%) | 1,4 | 1,2 | PCR+ | Sex, age, other | 16 | 8 |
| Shah, 2021 | UK | Cohort | 8/12/2020 | 3/3/2021 | 144 525 | 21 | 18 | 44 (11) | 113 253 | BNT162b2 (96%), ChAdOx1 (4%) | 1,3,4 | 1,2 | PCR+ | Sex, age, other | 3 | 9 |
| Sharma, 2021 | USA | Cohort | 1/9/2021 | 25/11/2021 | 258 260 | 94 | 18 | 72 (10) | 258 260 | BNT162b2 (57%), mRNA-1273 (43%) | 1,3,4 | 3 | PCR+ | Sex, age, other | 16 | 8 |
| Sheikh, 2021 | UK | Cohort | 1/4/2021 | 27/9/2021 | 102 479 | 52 | 18 | n.r. | 62 246 | BNT162b2 (50%), ChAdOx1 (50%) | 4 | 1,2 | PCR+ | Sex, age, other | 7 | 7 |
| Sheikh, 2021 | UK | Cohort | 1/4/2021 | 6/6/2021 | n.r. | n.r. | 18 | n.r. | n.r. | BNT162b2, ChAdOx1 | 1,2,4 | 1,2 | PCR+ | Sex, age, other | 16 | 7 |
| Shen, 2022 | USA | Cohort | 1/1/2021 | 7/12/2021 | 5 536 | 37 | n.r. | 57 (25) | 4 283 | BNT162b2 (48%), mRNA-1273 (48%), Ad26.COV2.S (4%) | 1,4 | 2 | PCR+ | Sex, age, other | 3 | 9 |
| Shrestha, 2021 | USA | Cohort | 16/12/2020 | 15/5/2021 | 46 866 | n.r. | 18 | 42 (13) | 28 223 | BNT162b2 (37%), mRNA-1273 (63%) | 1,4 | 2 | PCR+ | Age, other | 1 | 7 |
| Shrestha, 2022 | USA | Cohort | 16/12/2020 | 27/12/2021 | 52 238 | 22 | 18 | 42 (13) | 36 922 | BNT162b2, mRNA-1273 | 1,2,4 | 2 | PCR+ | Sex, age, other | 8 | 8 |
| Shrotri, 2021 | UK | Cohort | 8/12/2020 | 15/3/2021 | 10 412 | 30 | 65 | 86 (11) | 9 160 | ChAdOx1 (67%), BNT162b2 (33%) | 1,4 | 1 | PCR+ | Sex, age, other | 10 | 7 |
| Schiavetti, 2022 | Italy | Cohort | 1/3/2021 | 24/12/2021 | 19 641 | n.r. | n.r. | n.r. | 19 641 | BNT162b2, mRNA-1273 | 3,4 | 2 | PCR+ | Other | 2 | 4 |
| Sibbel, 2021 | USA | Cohort | 1/1/2021 | 25/2/2021 | 143 826 | 57 | 18 | 69 (n.r.) | 35 206 | BNT162b2 (35%), mRNA-1273 (65%) | 1,2,4 | 1,2 | PCR+ | Sex, age, other | 8 | 7 |
| Singer, 2021 | Israel | Cohort | 11/12/2020 | 25/3/2021 | 343 | 55 | 16 | 40 (n.r.) | 63 | BNT162b2 (100%) | 1,2,3,4 | 2 | PCR+ | None | 3 | 6 |
| Singh, 2021 | India | Case-control | 1/4/2021 | 30/6/2021 | 1 731 | 67 | 45 | 55 (13) | 812 | ChAdOx1 (79%), BBV152 (21%) | 1,4 | 1,2 | PCR+/Ag+ | Sex, age, other | 4 | 3 |
| Skowronski, 2021 | Canada | Case-control | 4/4/2021 | 1/5/2021 | 16 993 | 49 | 70 | n.r. | 12 451 | BNT162b2 (85%), mRNA-1273 (15%) | 1,4 | 1 | PCR+ | Sex, age, other | 30 | 7 |
| Skowronski, 2021 | Canada | Case-control | 30/5/2021 | 2/10/2021 | 2 154 033 | 41 | 18 | 45 (30) | 1 832 226 | BNT162b2 (69%), mRNA-1273 (19%), ChAdOx1 (3%), mRNA+AdV (9%) | 1,3,4 | 2 | PCR+ | Sex, age, other | 345 | 8 |
| Skowronski, 2022 | Canada | Case-control | 4/4/2021 | 2/10/2021 | 111 405 | 48 | 50 | 58 (9) | 52 517 | ChAdOx1 (19%), BNT162b2 (63%), mRNA-1273 (18%) | 1,3,4 | 1 | PCR+ | Sex, age, other | 52 | 7 |
| Spensley, 2022 | UK | Cohort | 1/12/2021 | 16/1/2022 | 1 110 | 61 | n.r. | 65 (21) | 1 040 | BNT162b2 (53%), ChAdOx1 (47%) | 1,4 | 2,3 | PCR+ | Other | 4 | 6 |
| Sritipsukho, 2022 | Thailand | Case-control | 25/7/2021 | 23/10/2021 | 3 353 | 38 | 18 | n.r. | 2 379 | CoronaVac (49%), ChAdOx1 (51%) | 1,4 | 2 | PCR+ | Sex, age, other | 5 | 7 |
| Starrfelt, 2021 | Norway | Cohort | 1/1/2021 | 27/9/2021 | 4 293 544 | 50 | 18 | 49 (n.r.) | 3 843 420 | BNT162b2 (65%), mRNA-1273 (11%), mRNA (16%), ChAdOx1 (4%)mRNA+AdV(;4% | 1,3,4 | 1,2 | PCR+ | Sex, age, other | 54 | 8 |
| Starrfelt, 2021 | Norway | Cohort | 1/11/2020 | 15/6/2021 | 31 489 | 31 | n.r. | 87 (11) | 27 038 | BNT162b2 (100%) | 1,4 | 1,2 | PCR+ | Sex, age, other | 4 | 5 |
| Suah, 2021 | Malaysia | Cohort | 1/4/2021 | 15/9/2021 | 1 286 881 | 57 | 18 | n.r. | 498 417 | BNT162b2 (35%), ChAdOx1 (10%), CoronaVac (54%) | 4 | 1,2 | PCR+/Ag+ | Age, other | 10 | 6 |
| Suah, 2022 | Malaysia | Cohort | 1/9/2021 | 30/9/2021 | 9 927 350 | 49 | 15 | 45 (n.r.) | 9 927 350 | BNT162b2 (44%), CoronaVac (56%) | 1,4 | 2 | PCR+ | Sex, age, other | 32 | 7 |
| Sultan, 2022 | Jordan | Cross-sectional | 15/1/2021 | 30/9/2021 | 3 089 | n.r. | 18 | 34 (10) | 2 855 | BNT162b2 (60%), BBIBP-CorV (24%), ChAdOx1 (16%) | 1,4 | 2 | PCR+ | Other | 1 | 5 |
| Swift, 2021 | USA | Cohort | 1/1/2021 | 31/3/2021 | 71 152 | 30 | 18 | 43 (13) | 47 221 | BNT162b2 (91%), mRNA-1273 (9%) | 1,4 | 1,2 | PCR+ | Sex, age, other | 4 | 8 |
| Syed, 2022 | Qatar | Cohort | 16/12/2020 | 31/10/2021 | 1 241 501 | 52 | 12 | 36 (n.r.) | 1 004 766 | BNT162b2 (70%), mRNA-1273 (30%) | 1,4 | 1,2 | PCR+ | Sex, age, other | 126 | 8 |
| Šmíd, 2022 | Czechia | Cohort | 7/12/2021 | 13/2/2022 | 8 282 080 | n.r. | 0 | 46 (n.r.) | 6 774 951 | BNT162b2 (80%), mRNA-1273 (7%), ChAdOx1 (6%), Ad26.COV2.S (6%) | 3,4 | 2,3 | PCR+ | Sex, age, other | 8 | 8 |
| Tabak, 2021 | USA | Case-control | 1/5/2021 | 7/8/2021 | 1 237 097 | 41 | 18 | 37 (23) | 591 493 | BNT162b2 (57%), mRNA-1273 (35%), Ad26.COV2.S (8%) | 1,4 | 2 | PCR+ | Age, other | 21 | 5 |
| Tande, 2021 | USA | Cohort | 17/12/2020 | 8/2/2021 | 48 333 | 48 | 18 | 55 (18) | n.r. | BNT162b2, mRNA-1273 | 1,4 | 1,2 | PCR+ | Sex, age, other | 2 | 6 |
| Tande, 2021 | USA | Cohort | 1/1/2021 | 15/8/2021 | 56 917 | 47 | 18 | 60 (17) | 27 376 | BNT162b2 (56%), mRNA-1273 (33%), mRNA (11%) | 1,4 | 1,2 | PCR+/Ag+ | Sex, age, other | 7 | 7 |
| Tang, 2021 | USA | Cohort | 17/12/2020 | 20/3/2021 | 5 217 | 43 | 18 | n.r. | 3 052 | BNT162b2 (100%) | 1,2,4 | 1,2 | n.r. | None | 3 | 4 |
| Tang, 2021 | Qatar | Case-control | 21/12/2020 | 7/9/2021 | 99 521 | 55 | 18 | 27 (24) | 62 973 | BNT162b2 (54%), mRNA-1273 (46%) | 1,2,3,4 | 1,2 | PCR+ | Sex, age, other | 31 | 8 |
| Tartof, 2021 | USA | Cohort | 14/12/2020 | 8/8/2021 | 3 436 957 | 48 | 12 | 45 (32) | 1 146 768 | BNT162b2 (100%) | 1,3,4 | 2 | PCR+ | Sex, age, other | 51 | 9 |
| Tartof, 2022 | USA | Cohort | 14/12/2020 | 5/12/2021 | 3 133 075 | 53 | 18 | 45 (29) | 1 173 804 | BNT162b2 (100%) | 1,3,4 | 1,2,3 | PCR+ | Sex, age, other | 80 | 9 |
| Tenforde, 2021 | USA | Case-control | 1/1/2021 | 26/3/2021 | 417 | 52 | 65 | 73 (n.r.) | 119 | BNT162b2 (53%), mRNA-1273 (47%) | 2,4 | 1,2 | PCR+ | Sex, age, other | 2 | 7 |
| Tenforde, 2021 | USA | Case-control | 11/3/2021 | 15/8/2021 | 4 513 | 51 | n.r. | 59 (24) | 1 700 | BNT162b2 (61%), mRNA-1273 (39%) | 3,4 | 2 | PCR+/Ag+ | Sex, age, other | 16 | 7 |
| Tenforde, 2021 | USA | Case-control | 11/3/2021 | 14/7/2021 | 3 089 | 51 | 18 | 59 (23) | 1 129 | BNT162b2 (59%), mRNA-1273 (41%) | 1,3,4 | 2 | PCR+/Ag+ | Sex, age, other | 12 | 7 |
| Tenforde, 2021 | USA | Case-control | 11/3/2021 | 5/5/2021 | 1 212 | 51 | 18 | 59 (22) | 483 | BNT162b2 (41%), mRNA-1273 (59%) | 3,4 | 1,2 | PCR+ | Sex, age, other | 13 | 7 |
| Tenforde, 2022 | USA | Case-control | 19/8/2021 | 15/12/2021 | 2 952 | 51 | 18 | 62 (n.r.) | 1 563 | BNT162b2 (60%), mRNA-1273 (40%), mRNA (1%) | 3,4 | 2,3 | PCR+/Ag+ | Age, other | 4 | 5 |
| Thakkar, 2022 | USA | Cohort | 1/8/2021 | 12/11/2021 | 1 128 | 50 | 11 | n.r. | 829 | BNT162b2 (100%) | 1,2,4 | 2 | PCR+/Ag+ | None | 2 | 5 |
| Thiruvengadam, 2021 | India | Case-control | 1/4/2021 | 31/5/2021 | 5 143 | 67 | n.r. | 34 (17) | 4 445 | ChAdOx1 (100%) | 1,2,4 | 1,2 | PCR+ | Sex, age, other | 4 | 7 |
| Thompson, 2021 | USA | Cohort | 14/12/2020 | 13/3/2021 | 3 950 | 38 | 18 | 44 (n.r.) | 2 961 | BNT162b2 (63%), mRNA-1273 (30%) | 1,4 | 1,2 | PCR+ | Other | 2 | 6 |
| Thompson, 2021 | USA | Case-control | 1/1/2021 | 22/6/2021 | 41 552 | 53 | 50 | 74 (11) | 16 491 | BNT162b2 (48%), mRNA-1273 (49%), Ad26.COV2.S (3%) | 3,4 | 1,2 | PCR+ | Age, other | 33 | 6 |
| Thompson, 2021 | USA | Cohort | 12/12/2020 | 10/4/2021 | 3 975 | 38 | 18 | n.r. | 3 179 | BNT162b2 (67%), mRNA-1273 (33%) | 1,4 | 1,2 | PCR+ | other | 8 | 6 |
| Thompson, 2022 | UK | Case-control | 26/8/2021 | 5/1/2022 | 222 772 | 41 | 18 | n.r. | 117 689 | BNT162b2 (61%), mRNA-1273 (38%), mRNA (1%) | 3,4 | 2,3 | PCR+ | Age, other | 6 | 6 |
| Thompson, 2022 | USA | Case-control | 26/8/2021 | 22/1/2022 | 241 204 | 41 | 18 | 50 (n.r.) | 130 331 | BNT162b2 (61%), mRNA-1273 (39%) | 2,3,4 | 2,3 | PCR+ | Sex, age, other | 44 | 6 |
| Tseng, 2022 | USA | Case-control | 6/12/2021 | 31/12/2021 | 148 917 | 44 | 18 | 38 (21) | 89 837 | mRNA-1273 (100%) | 1,3,4 | 1,2,3 | PCR+ | Sex, age, other | 29 | 9 |
| Vasileiou, 2021 | UK | Cohort | 8/12/2020 | 22/2/2021 | 4 409 588 | 48 | 18 | 65 (16) | 1 331 993 | BNT162b2 (53%), ChAdOx1 (47%) | 3,4 | 1 | PCR+ | Sex, age, other | 33 | 8 |
| Veneti, 2022 | Norway | Cohort | 6/12/2021 | 9/1/2022 | 91 005 | 50 | 0 | 29 (30) | 45 907 | BNT162b2 (78%), mRNA-1273 (22%) | 3,4 | 1,2,3 | PCR+ | Sex, age, other | 40 | 7 |
| Victor, 2021 | India | Cohort | 21/1/2021 | 19/5/2021 | 10 600 | n.r. | 18 | n.r. | 8 991 | ChAdOx1 (93%), BBV152 (7%) | 2,3,4 | 1,2 | PCR+ | None | 4 | 3 |
| Villela, 2021 | Brazil | Cohort | 17/1/2021 | 17/7/2021 | 65 210 769 | 43 | 18 | n.r. | 6 467 8797 | CoronaVac (33%), ChAdOx1 (57%), BNT162b2 (11%) | 3,4 | 1,2 | PCR+ | age, other | 40 | 6 |
| Vokó, 2021 | Hungary | Cohort | 22/1/2021 | 10/6/2021 | 7 774 642 | 48 | 16 | n.r. | 3 740 066 | BNT162b2 (40%), mRNA-1273 (6%), Gam-COVID- Vac (22%), ChAdOx1 (8%)BBIBP-CorV(;24% | 1,4 | 2 | PCR+/Ag+ | Sex, age, other | 66 | 5 |
| Waldman, 2021 | USA | Cohort | 15/12/2020 | 1/3/2021 | 16 156 | 37 | 18 | n.r. | 10 849 | BNT162b2 (63%), mRNA-1273 (37%) | 1,4 | 1,2 | PCR+ | None | 2 | 5 |
| Walsh, 2021 | Ireland | Cohort | 29/12/2020 | 22/2/2021 | 4 458 | n.r. | 18 | n.r. | 3 805 | BNT162b2 (100%) | 1,4 | 1 | PCR+ | None | 1 | 4 |
| Whitaker, 2022 | UK | Cohort | 7/12/2020 | 16/5/2021 | 5 264 362 | n.r. | 16 | n.r. | 3 099 242 | BNT162b2 (30%), ChAdOx1 (70%) | 2,3,4 | 1,2 | PCR+ | Sex, age, other | 106 | 8 |
| Wickert, 2021 | USA | Cohort | 1/3/2021 | 1/5/2021 | 4 188 | n.r. | 17 | n.r. | 3 560 | BNT162b2 (100%) | 1,4 | 1,2 | Ag+ | None | 3 | 3 |
| Williams, 2021 | Canada | Cohort | 11/4/2021 | 10/5/2021 | 286 | n.r. | n.r. | n.r. | 256 | BNT162b2 (58%), mRNA-1273 (42%) | 1,2,3,4 | 2 | PCR+ | None | 5 | 4 |
| Wu, 2021 | USA | Cohort | 15/12/2020 | 4/5/2021 | 58 304 | 94 | 18 | 74 (9) | 29 152 | BNT162b2 (54%), mRNA-1273 (46%) | 1,4 | 1,2 | PCR+/Ag+ | age, other | 2 | 7 |
| Yassi, 2021 | Canada | Cohort | 15/12/2020 | 13/5/2021 | 25 558 | n.r. | 18 | n.r. | 22 118 | BNT162b2 (93%), mRNA-1273 (7%) | 1,4 | 1,2 | PCR+ | Age, other | 2 | 8 |
| Young-Xu, 2021 | USA | Case-control | 14/12/2020 | 7/3/2021 | 75 546 | 90 | 18 | n.r. | 7 672 | BNT162b2 (41%), mRNA-1273 (59%) | 1,3,4 | 1,2 | PCR+/Ag+ | Sex, age, other | 27 | 7 |
| Young-Xu, 2021 | USA | Case-control | 1/1/2021 | 30/9/2021 | 14 238 | 100 | 65 | n.r. | n.r. | BNT162b2, mRNA-1273 | 1,4 | 2 | PCR+ | Sex, age, other | 16 | 5 |
| Young-Xu, 2022 | USA | Case-control | 1/12/2021 | 31/12/2021 | 69 215 | n.r. | n.r. | n.r. | 48 451 | BNT162b2, mRNA-1273 | 1,3,4 | 2,3 | PCR+ | Age, other | 7 | 6 |
| Zacay, 2021 | Israel | Cohort | 1/1/2021 | 11/2/2021 | 6 286 | 41 | 16 | 45 (19) | 4 386 | BNT162b2 (100%) | 1,4 | 1,2 | PCR+ | None | 2 | 6 |
| Zambrano, 2022 | USA | Case-control | 1/7/2021 | 9/12/2021 | 283 | 53 | 12 | 15 (3) | 70 | BNT162b2 (100%) | 3,4 | 2 | PCR+/Ag+ | Sex, age, other | 1 | 8 |
| Zaqout, 2021 | Qatar | Cohort | 1/1/2021 | 30/3/2021 | 199 219 | 58 | 16 | 42 (22) | 131 326 | BNT162b2 (100%) | 1,3,4 | 1,2 | PCR+ | None | 4 | 6 |

n.r. … not reported; COVID-19: 1 ... SARS-CoV-2 infection; 2 ... COVID-19 of any severity; 3 ... hospitalization; 4 ... death; Immunization: 1 … partial; 2 … full; 3 … booster
