# Supplementary material for: Risk factors affecting COVID-19 vaccine effectiveness identified from 290 cross-country observational studies until February 2022: a meta-analysis and meta-regression
Source: BMC Med. 2022 Nov 25;20:461. doi: 10.1186/s12916-022-02663-z (PMC9701077; doi:10.1186/s12916-022-02663-z)
Supplement: Supplementary file 7 — Additional file 7. Pooled and adjusted vaccine effectiveness against severe COVID-19 after partial mRNA and AdV immunization (Figure S2). [file 12916_2022_2663_MOESM7_ESM.docx]

**Additional file 7**

**Figure S2.** Pooled and adjusted vaccine effectiveness against severe COVID-19 for investigated predictors with VE density displayed in truncated violin plots after partial mRNA and AdV immunization, including a minimum 50% VE threshold


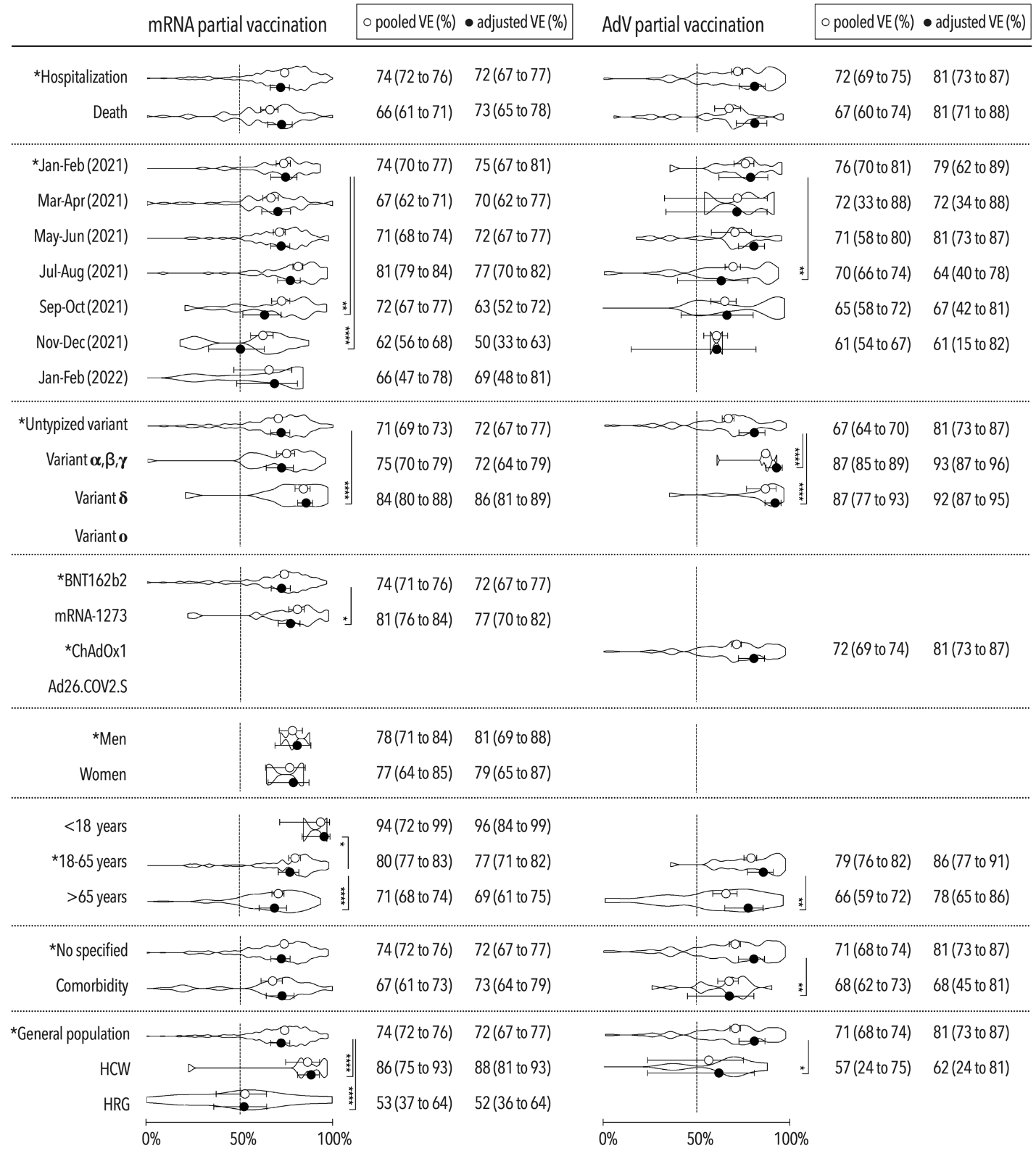


AdV … adenoviral vector vaccines; VE … vaccine effectiveness; HCW … healthcare workers; HRG … high-risk group (individuals in long-term care and residential care homes); * … p<0·05; ** … p<0·01; *** … p<0·001; **** … p<0·0001
