# Supplementary material for: Risk factors affecting COVID-19 vaccine effectiveness identified from 290 cross-country observational studies until February 2022: a meta-analysis and meta-regression
Source: BMC Med. 2022 Nov 25;20:461. doi: 10.1186/s12916-022-02663-z (PMC9701077; doi:10.1186/s12916-022-02663-z)
Supplement: Supplementary file 8 — Additional file 8. The quality of evidence for factor-stratified outcomes (Table S7). [file 12916_2022_2663_MOESM8_ESM.docx]

**Additional file 8**

**Table S7. The quality of evidence for factor-stratified outcomes**

|  | **mRNA** |  |  |  |  |  | **AdV** |  |  |  |
| --- | --- | --- | --- | --- | --- | --- | --- | --- | --- | --- |
|  | **Any COVID** |  |  | **Severe COVID** |  |  | **Any COVID** |  | **Severe COVID** |  |
| **Factor** | **1 dose** | **2 doses** | **3 doses** | **1 dose** | **2 doses** | **3 doses** | **1 dose** | **2 doses** | **1 dose** | **2 doses** |
| **Infection** | Acceptable | Acceptable | Acceptable |  |  |  | Acceptable | Acceptable |  |  |
| **Disease** | Acceptable | Acceptable | Unclear |  |  |  | Unclear | Acceptable |  |  |
| **Hospitalization** |  |  |  | Acceptable | Acceptable | Acceptable |  |  | Acceptable | Acceptable |
| **Death** |  |  |  | Acceptable | Acceptable | Acceptable |  |  | Unclear | Acceptable |
|  |  |  |  |  |  |  |  |  |  |  |
| **Jan-Feb 2021** | Acceptable | Acceptable |  | Acceptable | Acceptable |  | Unclear | Acceptable | Acceptable |  |
| **Mar-Apr 2021** | Acceptable | Acceptable |  | Acceptable | Acceptable |  | Acceptable | Acceptable | Unclear | Acceptable |
| **May-Jun 2021** | Acceptable | Acceptable |  | Acceptable | Acceptable |  | Acceptable | Acceptable | Unclear | Acceptable |
| **Jul-Aug 2021** | Acceptable | Unclear |  | Acceptable | Acceptable |  | Acceptable | Acceptable | Acceptable | Acceptable |
| **Sep-Oct 2021** | Unclear | Acceptable |  | Acceptable | Unclear |  | Unclear | Acceptable | Acceptable | Acceptable |
| **Nov-Dec 2021** | Acceptable | Acceptable | Acceptable | Acceptable | Acceptable | Acceptable | Acceptable | Acceptable | Unclear | Acceptable |
| **Jan-Feb 2021** | Acceptable | Acceptable | Unclear | Acceptable | Unclear | Acceptable | Unclear | Unclear |  |  |
|  |  |  |  |  |  |  |  |  |  |  |
| **BNT162b2** | Acceptable | Acceptable | Unclear | Acceptable | Acceptable | Acceptable |  |  |  |  |
| **mRNA-1273** | Unclear | Acceptable | Acceptable | Acceptable | Acceptable | Unclear |  |  |  |  |
| **ChAdOx1** |  |  |  |  |  |  | Acceptable | Acceptable | Unclear | Acceptable |
| **Ad26.COV2.S** |  |  |  |  |  |  |  | Acceptable |  | Acceptable |
|  |  |  |  |  |  |  |  |  |  |  |
| **Men** | Unclear | Acceptable | Unclear | Unclear | Unclear | Unclear | Unclear | Unclear | Unclear | Unclear |
| **Women** | Acceptable | Acceptable | Unclear | Unclear | Unclear | Unclear | Unclear | Unclear | Unclear | Unclear |
|  |  |  |  |  |  |  |  |  |  |  |
| **<18 years** | Acceptable | Unclear |  | Acceptable | Unclear | Unclear |  | Acceptable |  |  |
| **18-65 years** | Acceptable | Acceptable | Acceptable | Acceptable | Acceptable | Unclear | Acceptable | Acceptable | Acceptable | Acceptable |
| **>65 years** | Acceptable | Acceptable | Unclear | Acceptable | Acceptable | Unclear | Acceptable | Unclear | Unclear | Acceptable |
|  |  |  |  |  |  |  |  |  |  |  |
| **No specified comorbidity** | Acceptable | Acceptable | Acceptable | Acceptable | Acceptable | Acceptable | Acceptable | Acceptable | Acceptable | Acceptable |
| **Comorbidity** | Acceptable | Acceptable | Acceptable | Unclear | Acceptable | Unclear | Acceptable | Unclear | Acceptable | Unclear |
|  |  |  |  |  |  |  |  |  |  |  |
| **Untyped variants** | Unclear | Acceptable | Acceptable | Acceptable | Acceptable | Acceptable | Acceptable | Acceptable | Acceptable | Acceptable |
| **𝛂,β,𝛄 variants** | Acceptable | Acceptable |  | Acceptable | Acceptable |  | Acceptable | Acceptable | Unclear | Acceptable |
| **𝛅 variant** | Acceptable | Acceptable | Acceptable | Acceptable | Acceptable | Acceptable | Acceptable | Acceptable | Unclear | Acceptable |
| **𝛐 variant** | Acceptable | Acceptable | Acceptable | Unclear | Unclear | Acceptable | Unclear | Unclear |  |  |
|  |  |  |  |  |  |  |  |  |  |  |
| **General population** | Unclear | Acceptable | Acceptable | Acceptable | Acceptable | Acceptable | Acceptable | Acceptable | Acceptable | Acceptable |
| **HCW** | Unclear | Unclear |  | Unclear | Unclear |  | Unclear | Unclear | Unclear | Acceptable |
| **HRG** | Acceptable | Acceptable | Unclear | Acceptable | Acceptable | Unclear | Unclear | Unclear |  | Unclear |

HCW … healthcare workers; HRG … high-risk group (individuals living in long-term care or residential homes)
